# Rice *EMF3* Alleles Adjust Flower Opening Time to Enhance the Seed Setting Rate Under High Temperature Stress

**DOI:** 10.1111/pbi.70653

**Published:** 2026-04-09

**Authors:** Takuma Ishizaki, Yoichi Hashida, Hideyuki Hirabayashi, Kazuhiro Sasaki, Hiroki Tokunaga, Eliza Vie M. Simon‐Ada, Masataka Wakayama, Toshiyuki Takai, Hiroki Saito, Atsushi J. Nagano, Hitoshi Sakakibara, Mikiko Kojima, Yumiko Takebayashi, Sung‐Ryul Kim, Ryo Matsushima, Michael J. Thomson, Kazuhiko Sugimoto, Ken‐Ichiro Hibara, Tsutomu Ishimaru

**Affiliations:** ^1^ Tropical Agriculture Research Front Japan International Research Center for Agricultural Sciences (JIRCAS) Ishigaki Japan; ^2^ Faculty of Agriculture Takasaki University of Health and Welfare Takasaki Japan; ^3^ Institute of Crop Science, National Agriculture and Food Research Organization (NARO) Tsukuba Japan; ^4^ Biological Resources and Post‐Harvest Division Japan International Research Center for Agricultural Sciences (JIRCAS) Tsukuba Japan; ^5^ Graduate School of Agricultural and Life Sciences The University of Tokyo Nishitokyo Japan; ^6^ Plant Breeding, Genetics, and Biotechnology Division International Rice Research Institute (IRRI) Metro Manila Philippines; ^7^ Fit for Future Genetic Resources Unit International Rice Research Institute (IRRI) Metro Manila Philippines; ^8^ Institute for Advanced Biosciences, Keio University Tsuruoka Japan; ^9^ Integrated Medical and Agricultural School of Public Health Ehime University Matsuyama Japan; ^10^ Crop, Livestock and Environment Division Japan International Research Center for Agricultural Sciences (JIRCAS) Tsukuba Japan; ^11^ Faculty of Agriculture Ryukoku University Otsu Japan; ^12^ Bioscience and Biotechnology Center Nagoya University Nagoya Japan; ^13^ Graduate School of Bioagricultural Sciences Nagoya University Nagoya Japan; ^14^ RIKEN Center for Sustainable Resource Science Tsurumi Japan; ^15^ Rice Breeding Innovations Department International Rice Research Institute (IRRI) Metro Manila Philippines; ^16^ Institute of Plant Science and Resources Okayama University Kurashiki Japan; ^17^ Department of Soil and Crop Sciences Texas A&M University College Station Texas USA; ^18^ Graduate School of Agricultural Regional Vitalization Kibi International University Minamiawaji Japan; ^19^ Central Region Agricultural Research Center National Agriculture and Food Research Organization (NARO) Joetsu Japan

**Keywords:** *EARLY‐MORNING FLOWERING 3*, flower opening time, heat stress, rice, seed setting rate

## Abstract

To safeguard global food security against rapid population growth and a warming world, the effective genetic improvement of cereals is imperative. Flower opening time (FOT) critically affects the seed setting rate. In this study, we identified a gene, *EARLY‐MORNING FLOWERING 3* (*EMF3*), in which single‐nucleotide substitutions strongly modulate FOT in rice in a semi‐dominant manner, resulting in wide variation in FOT from earlier to later FOT than the wild‐type. *EMF3* knock‐out mutants showed significantly reduced FOT synchrony and disrupted anther dehiscence, leading to fertilisation failure. *EMF3* encodes a plasma membrane‐localised polypeptide of 723 amino acids with an armadillo repeat fold and four transmembrane segments. Furthermore, *EMF3* is specifically expressed in the anthers starting from nighttime on the day of flowering, with substantial impacts on the transcriptomes of both anther and lodicule, which suggested an exclusive role of *EMF3* in flowering events. Modifying *EMF3* alleles of *O. sativa* enabled the adjustment of FOT among *Oryza* species and subspecies, potentially facilitating cross‐fertilisation by overcoming one of the major challenges of inter‐specific hybridisation to exploit heterosis. Introducing the *EMF3* alleles with the earlier FOT into popular rice cultivars resulted in flowering at an earlier time of day when the temperature was cooler, efficiently increasing seed setting rate under heat stress. This discovery unveils the novel mechanism of anther control of flower opening time through the *EMF3* gene, while also enabling the use of *EMF3* alleles in breeding strategies for efficient fertilisation for increasing hybrid rice seed production and mitigating future heat‐stress damage at flowering.

## 1 Introduction

Global surface temperatures have increased because of climate change (AR5 Climate Change 2013). The year 2024 was the hottest ever recorded. It was 1.60°C warmer than pre‐industrial levels, making it the first calendar year to exceed 1.5°C above that level (Global Climate Highlights 2024). Extreme weather events, such as heat waves, are likely to occur more frequently (Climate Change 2021). This trend challenges global agriculture, as major crop yields decrease because of heat stress (Zhao et al. 2017). Rice is a staple food for over half the global population. Owing to the rapid population increase in Asian and African countries, rice demand is projected to increase by 12% by 2033 compared to that in 2021–2023 (OECD and Food and Agriculture Organization of the United Nations 2024). The need for effective heat adaptation strategies to safeguard food security for the growing global population has been emphasised (Zhao et al. 2017).

Heat stress is particularly detrimental to crops during the flowering stage, with consequences including sterility (fertilisation failure), severely reducing crop yields (Prasad et al. 2017). In rice ( *Oryza sativa* L.), 35°C at flowering is the general threshold for reduced seed setting rate (Prasad et al. 2017). The primary causes of spikelet sterility include abnormal anther dehiscence and reduced pollen grain release from the stigma (Matsui et al. 2001; Matsui and Omasa 2002; Satake and Yoshida 1978). Increased sterility was observed in popular rice cultivars in Japan (Hasegawa et al. 2009), Laos, and southern India (Ishimaru, Xaiyalath, et al. 2016) when the flowering stage coincided with heat stress. One proposed adaptation strategy to mitigate yield loss in rice involves shifting the flower opening time (FOT) from its normal window (10:00–12:00) to the early morning when the temperature is relatively low (Satake and Yoshida 1978). In our previous studies, a QTL for early‐morning flowering (EMF) was detected on chromosome 3 (*qEMF3*) (Hirabayashi et al. 2015) using a wild rice, *O. officinalis*, as a donor genetic resource for early‐morning flowering (Ishimaru et al. 2010). The near‐isogenic line (NIL) carrying *qEMF3* had comparable grain yield to its recurrent *indica* parent (IR64) under normal temperature under the multiple field environments, whereas the NIL enhanced seed setting rate by 17% compared to its recurrent *indica* parent, IR64, under hot field conditions where flowering temperatures reached approximately 38°C, significantly mitigating yield loss (Ishimaru et al. 2022). *qEMF3* enhances seed setting under high temperature primarily through a heat‐escape strategy, rather than through increased heat tolerance (Hirabayashi et al. 2015).

Hybrid rice takes advantage of heterosis (hybrid vigour) via cross‐fertilisation between different parental lines and is expected to lead to substantial genetic gains in grain yield (Qian et al. 2016). The yield potential of inter‐subspecific (i.e., *japonica*/*indica*) hybrid rice is 30% higher than that of intra‐subspecific hybrid rice (i.e., *japonica*/*japonica*, *indica*/*indica*) (Qian et al. 2016; Zhang 2022). Nonetheless, major challenges remain in inter‐subspecific hybrid rice seed production. Recent progress in genetic studies on hybrid sterility is overcoming the incompatibility of wide crosses between *Oryza* species (Koide et al. 2018; Myint et al. 2024) and subspecies (Zhang 2022). Notably, however, different FOTs between parental lines can limit the chance of cross‐fertilisation, as the flowering of each rice spikelet is fleeting, occurring within a 1‐h window from the start of opening to complete closure (Hoshikawa 1993). Increasing seed production through the precise adjustment of FOT between parental lines will be a breakthrough technology in disseminating high‐yield inter‐subspecific hybrid rice cultivars (Wang et al. 2024).

Flower opening occurs concurrently with rapid lodicule expansion, which physically pushes the lemma and palea apart, unlocking them (Hoshikawa 1993). Therefore, previous genetic studies on FOT focused on the function of genes expressed in the lodicule (Wang et al. 2023) and genes such as *Diurnal FOT 1* (*DFOT1*)/*Early‐Morning Flowering 1* (*EMF1*) (Wang et al. 2022; Xu et al. 2022) and *OsMYB8* (Gou et al. 2024) were identified. Furthermore, research has highlighted the importance of jasmonic acid (JA) in FOT regulation (Ding et al. 2024; Li et al. 2018; Liu et al. 2017; Wang et al. 2024; Zhu et al. 2024). However, none of these previously identified genes were shown to alleviate the spikelet sterility caused by high temperatures. To support the adaptation of rice to global warming, it is essential to identify the gene responsible for the early morning flowering trait governed by *qEMF3*. The manipulation of genes related to the jasmonate pathway facilitated seed production in *indica*–*japonica* hybrid rice by shifting the FOT of a *japonica* parental line earlier, to that of an *indica* parental line (Wang et al. 2024). However, alleles in a single gene that can potentially provide a breakthrough in heat escape at flowering and hybrid rice seed production by creating wide variation in FOT have not yet been discovered. In this study, we report the identification of *EARLY‐MORNING FLOWERING 3* (*EMF3*), a causal gene for *qEMF3*. The identification of this causal gene could aid in elucidating the novel mechanisms underlying flower opening time, launch effective rice breeding to mitigate yield losses by escaping heat stress at flowering and facilitate inter‐subspecific hybrid rice seed production.

## 2 Results

### 2.1 Identification of *EMF3* Controlling FOT in Rice

**FIGURE 1:**
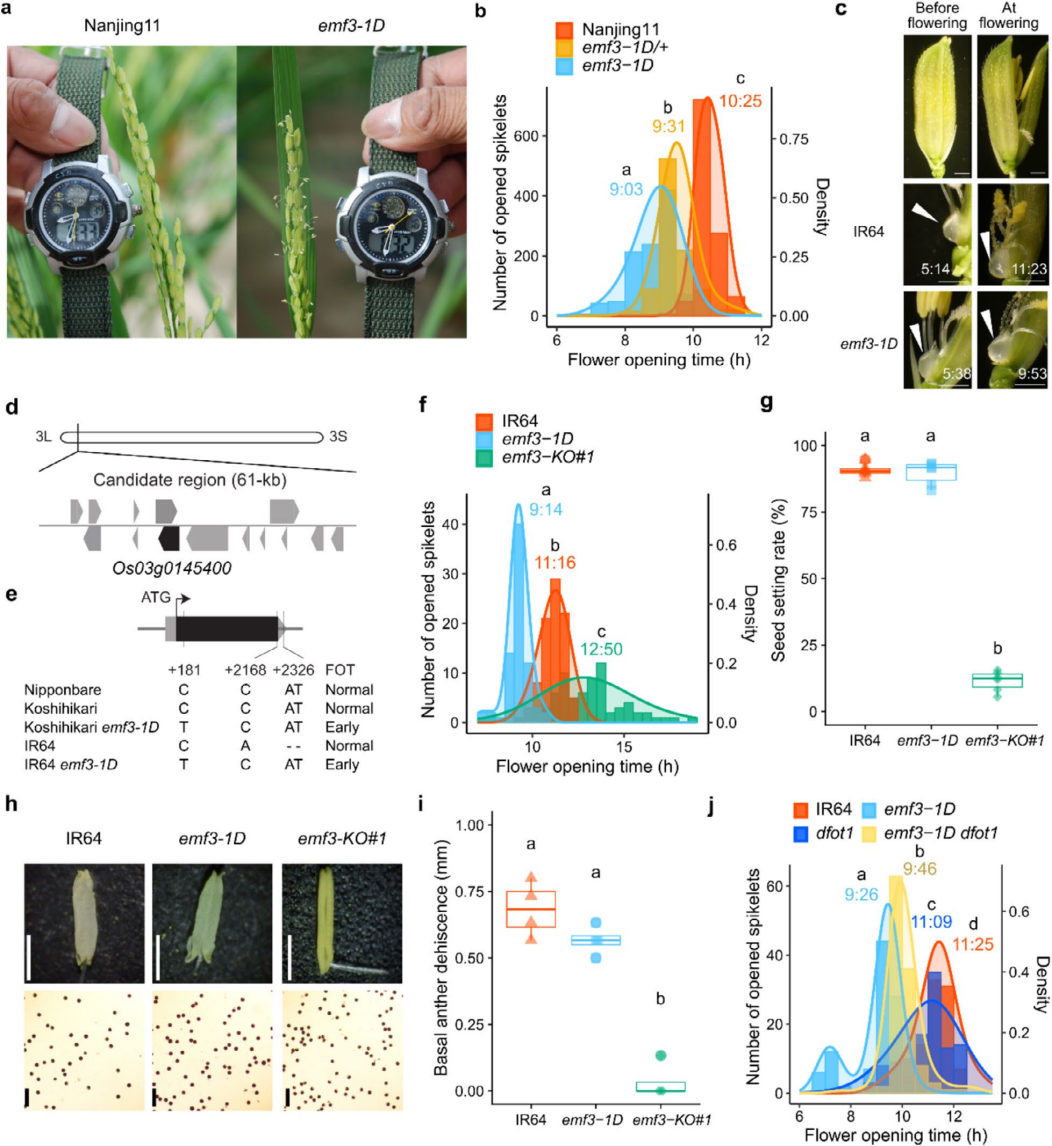
*EMF3* alleles confer genetic variation in FOT. (a) Panicles of Nanjing 11 and the *emf3‐1D* allele (Nanjing 11 background) around 08:30 on the same day. (b) Observation of FOT for the genetic background of Nanjing 11 (wild type), and its heterozygous and homozygous *emf3‐1D* alleles. (c) Images of lodicule before flowering (around 5:00 under dark condition) and at flowering (11:23 for IR64 and 9:53 for *emf3‐1D* allele with IR64 background both under light condition). Bars in panels = 400 μm (upper two panels) and 200 μm (middle and lower four panels). (d) Predicted genes in the candidate region through high resolution mapping. (e) Polymorphisms in the transcription of *Os03g0145400* among Nipponbare, Koshihikari, IR64 and the *emf3‐1D* allele (Koshihikari and IR64 background). (f) Observation of FOT and (g) Seed setting rate of the *emf3‐KO#1* mutant. Observation of FOT was conducted on the same day among genotypes. Each boxplot consists of six biological replicates, whereas each small symbol represents the observed raw value in each replicate. Different letters indicate significant differences at *p* < 0.01 according to Tukey–Kramer's method. (h) anthers (upper) and Iodine‐stained pollen (lower) of IR64, *emf3‐1D* and *emf3‐KO#1*. Bars in upper panels = 1 mm; bars in lower panels = 100 μm. (i) Basal anther dehiscence length of IR64, *emf3‐1D* and *emf3‐KO#1*. Different letters indicate significant at the 1% level according to Tukey's method. Each boxplot consists of 4 biological replicates (spikelets), whereas each small symbol represents the observed raw value in each replicate. (j) Observation of FOT for the *dfot1* mutants, *emf3‐1D* allele, *dfot1* and *emf3‐1D* allele in the IR64 background. In b, f and j, time to reach peak FOT is indicated in each genotype, and the histograms represent the raw data of number of opened spikelets at each time point. Number of opened spikelets is based on observations from 14 to 16 panicles (b) and four panicles per genotype (f, j). The shaded areas indicate the estimated probability density. The times indicated in each panel represent the peak FOTs, estimated from the probability density. Different letters indicate statistically significant differences in peak FOT between genotypes, on the basis of Bonferroni‐adjusted 95% confidence intervals calculated with the bootstrap method.

We attempted to clone *EMF3*, the causal gene for *qEMF3* that advances the FOT in rice (Hirabayashi et al. 2015) (Figure 1a) via a semi‐dominant effect (Figure 1b and Tables S1 and S2). Although FOT fluctuates with the environmental conditions, lodicule expansion occurred consistently earlier in the day in the early‐morning flowering (EMF) line than in wild‐type (Figure 1c), concomitantly with a rapid increase in fresh weight at flower opening (Figure S1). We designate an allele of this *EMF3* as ‘*emf3‐1D*’ and conducted high‐resolution mapping using more than 7000 F_2_ plants segregating near *EMF3* in two populations, followed by an analysis of the recombinant F_3_ and F_4_ lines. This approach narrowed the candidate region to a 61‐kb interval between markers RM14380 and HID3010 (Figure 1d and Figure S2a). We compared the DNA sequences at the *qEMF3* locus of *emf3‐1D* (as per whole genome sequencing), IR64 (from the TASUKE database; Kumagai et al. 2019), Koshihikari and Nipponbare (from RAP‐DB; Sakai et al. 2013). Although several polymorphisms were identified between the IR64 and Koshihikari backgrounds with the *emf3‐1D* allele and the IR64, Koshihikari and Nipponbare cultivars, we found that the polymorphism at the 181st position in the coding region, which is predicted by RAP‐DB within the candidate region, consistently associated with the early morning phenotype—a single nucleotide substitution from C to T (C in the cultivars and T in *emf3‐1D*) (Figure 1e). Two loci were annotated at this position: *Os03g0145300*, described as a glycosyl transferase and *Os03g0145400*, which had no described functional domain (Figure S2b). Reverse transcription‐PCR targeting potential transcripts near the polymorphism revealed that *Os03g0145400* and a homologue of *BT065989* (neighbouring *Os03g0145400*) were expressed in spikelets (Figure S2c). No transcripts for *Os03g0145300* were detected (Figure S2c). Additionally, PCR amplicon sizes using a primer pair designed to span the predicted intron of *Os03g0145400* were identical when using both genomic DNA and cDNA as templates, confirming that *Os03g0145400* does not contain the predicted intron (Figure S2c,d). Furthermore, RACE sequence analysis of *Os03g0145400* confirmed the absence of an intron in the transcript (Figure S3). This indicates that *Os03g0145400* is a transcriptional unit expressed in spikelets, and the C‐to‐T nucleotide substitution at the 181st position (Figure 1e) leads to the Leucine (L)‐to‐Phenylalanine (F) amino acid substitution at the 61st position in the *emf3‐1D* allele (Figures S4 and S5). Two observations concerning the C‐to‐T nucleotide substitution at the 181st position were particularly noteworthy. First, the *EMF3* allele of IRGC100947 (*O. officinalis*, CC genome), a donor for the early morning flowering phenotype (Ishimaru et al. 2010), was the same as that of rice cultivars without the C‐to‐T nucleotide substitution (Figures S4 and S5). Second, the *emf3‐1D* allele was unique, which popular *indica* and *japonica* cultivars/varieties, including parental varieties for the three‐line hybrid rice system developed at IRRI, IR58025B and IR68897B (Virmani et al. 1998), do not carry (Figures S4 and S5).

*EMF3* knock‐out mutant lines generated using CRISPR/Cas9 against an IR64 background with the *emf3‐1D* allele (*emf3*‐*KO#1, #2*), IR64 (*indica* subspecies, *emf3*‐*KO#3*), NERICA1 (tropical *japonica* subspecies, *emf3*‐*KO#4*) and Nipponbare (temperate *japonica* subspecies, *emf3*‐*KO#5*) exhibited scattered FOT, whereas their wild type exhibited a clear peak of FOT at the fixed window (we defined the wild type's pattern of FOT as ‘synchrony’ in this study) (Figure 1f, Figure S6b,d,f,g, Tables S1–S3), suggesting that *EMF3* alleles play a role in regulating FOT synchrony across both *indica* and *japonica* rice subspecies. At maturity, the *emf3*‐*KO#1* mutant showed only 10% seed setting rate, whereas the seed setting rate was around 90% both in IR64 and IR64 background with the *emf3‐1D* allele (Figure 1g). Compared to IR64 and IR64 background with the *emf3‐1D* allele, clear differences in plant appearance were observed, including a stay‐green phenotype and erect panicle in the *emf3*‐*KO#1* mutant (Figure S6h), possibly attributed to the very low seed setting rate. Close observation of flowering spikelets revealed that their pollen grains matured, as evidenced by starch staining with iodine solution (Figure 1h); however, their basal anther dehiscence was impaired (Figure 1h,i). This suggests that an absence of pollen release from the anther at flowering caused the very low seed setting rate. Overall, observation of the flowering‐related characteristics of *emf3*‐*KO* mutants indicated that *EMF3* contributes to FOT synchrony and is required for efficient fertilisation, consistent with its role in anther dehiscence.

Furthermore, FOT of *emf3‐1D* was compared with *dfot1* (Wang et al. 2022), a gene that controls FOT being highly expressed in the lodicules on the day of flowering (Figure S7). *dfot1* exhibited earlier flower opening than IR64; however, *dfot1* showed less FOT synchrony than the wild‐type or *emf3‐1D* (Figure 1j and Tables S1–S3). No additive effects were observed between *emf3‐1D* and *dfot1*, as the FOT distribution in *emf3‐1D dfot1* resembled that in *emf3‐1D* (Figure 1j). These results indicate that *emf3‐1D* is epistatic to *dfot1* in FOT regulation.

### 2.2 Amino Acid Substitutions of *EMF3* Lead to Genetic Variation in FOT

**FIGURE 2:**
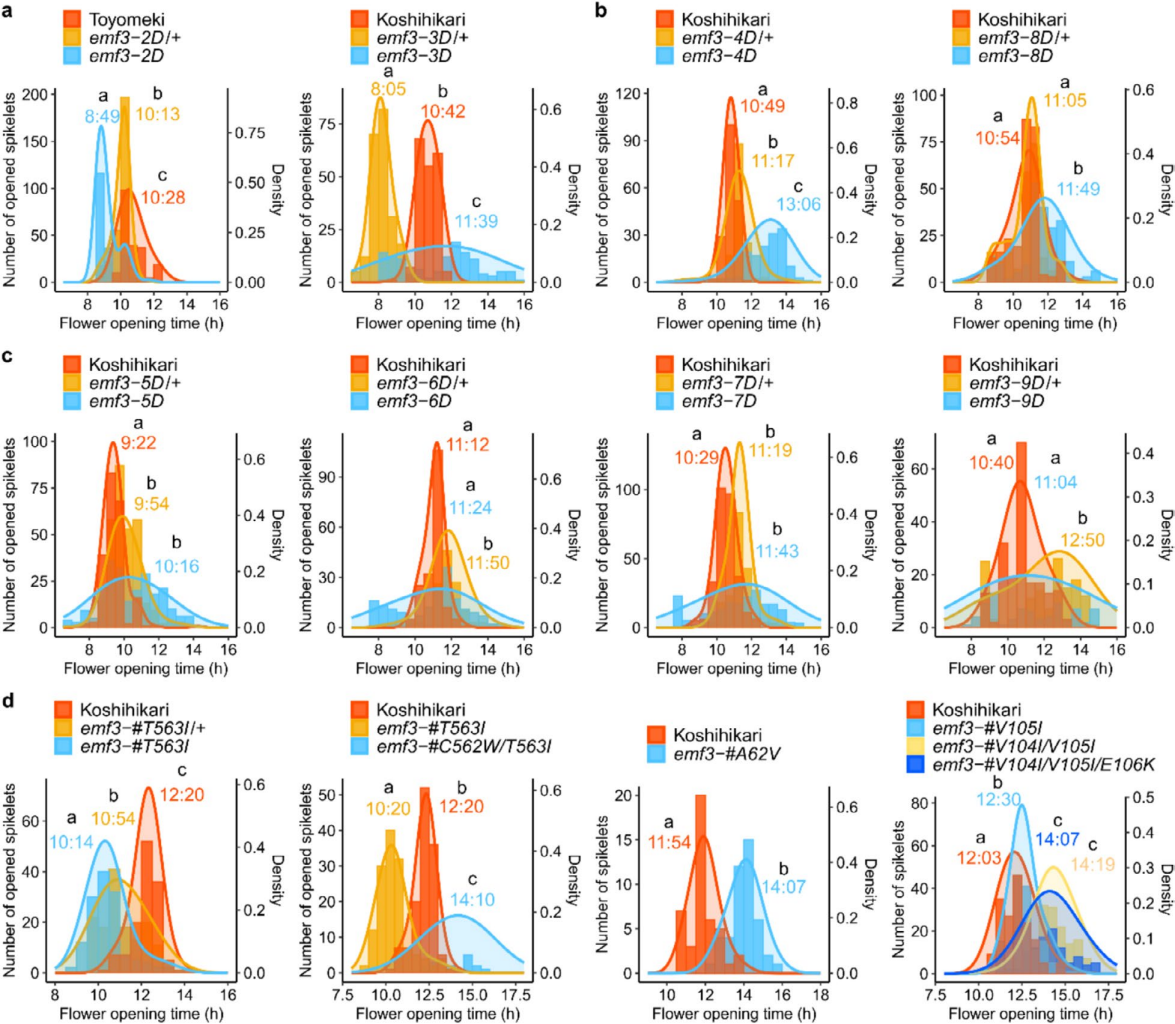
Observation of FOT in TILLING mutant lines (a–c) and genome‐edited (base editing) mutant lines (d). (a) FOT of mutants which belong to group (i) exhibiting shift to earlier FOT with synchrony. (b) FOT of mutants which belong to group (ii) exhibiting shift to later FOT with synchrony. (c) FOT of mutants which belong to group (iii) exhibiting low FOT synchrony. (d) FOT of genome‐edited mutants with the single, double and triple amino acid substitution(s). The histograms represent the raw data of number of opened spikelets at each time point on the basis of observations from four to six panicles per genotype. The shaded areas indicate the estimated probability density. The times indicated in each panel represent the peak FOT, estimated from the probability density. Different letters indicate statistically significant differences in peak FOT between genotypes, on the basis of Bonferroni‐adjusted 95% confidence intervals calculated using the bootstrap method.

To examine the effect of allelic variants on FOT, single‐nucleotide substitutions in *EMF3* causing single amino acid substitutions were searched in TILLING mutant panels. In total, 28 and seven mutant lines in *EMF3* were selected, with Koshihikari and Toyomeki (*japonica* cultivars) genetic backgrounds, respectively (Table S4). Among them, eight alleles that significantly changed FOT were identified via the development of specific markers for each allele (Table S5). They were designated as ‘*emf3‐2D*’ to ‘*emf3‐9D*’ (Tables S1, S2 and S4). The FOT patterns of the *EMF3* mutant lines were categorised into three groups: (i) shift to earlier FOT with synchrony, (ii) shift to later FOT with synchrony and (iii) low FOT synchrony. In addition to the *emf3‐1D*, homozygous *emf3‐2D* and heterozygous *emf3‐3D* alleles were categorised into group (i) (Figure 2a and Tables S1, S2 and S4), whereas the homozygous *emf3‐4D* and *emf3‐8D* alleles were categorised into group (ii) (Figure 2b and Tables S1, S2 and S4). In groups (i) and (ii), the peak FOT was 2.5 h earlier, 2.0 h earlier, 1.6 h earlier, 1.0 h later and 2.3 h later with the heterozygous *emf3‐3D*, homozygous *emf3‐1D*, homozygous *emf3‐2D*, homozygous *emf3‐8D* and homozygous *emf3‐4D* alleles, respectively, compared to the wild‐type (Figures 1f, 2a,b and Tables S1, S2 and S4). The homozygous *emf3‐3D*, *emf3‐5D*, *emf3‐6D*, *emf3‐7D* and *emf3‐9D* alleles were categorised into the group (iii) (Figure 2a,c and Tables S1, S2 and S4). Most of the heterozygous alleles had an intermediate FOT between the wild‐type and their homozygous alleles (Figure 2a–c). The *EMF3* homozygous mutant lines showed two different levels of seed setting rate; that of the *emf3‐1D*, *emf3‐2D* and *emf3‐8D* alleles was as high as their recurrent parent (around 80%–90%) (Figures 1g and S8a,b), whereas that of the other alleles was very low (around 20%–40%) (Figure S8a–c). Every heterozygous *EMF3* allele showed a high seed setting rate (Figure S8a–c).

Using base editing technology, we further explored whether amino acid substitutions that were not found in *EMF3* TILLING mutant panels change FOT. Genetic manipulation of the wild‐type allele into the *emf3‐2D* allele in *EMF3* (*emf3‐#T563I*) resulted in an earlier FOT (Figure 2d and Tables S1, S2 and S3), demonstrating the reproducibility of the earlier FOT observed in the *emf3‐2D* TILLING mutant line (Figure 2a). A combination of amino acid substitutions at the 562nd and 563rd positions (*emf3‐#C562W/T563I*) resulted in FOT group (iii), with low FOT synchrony (Figure 2d and Tables S1, S2 and S3). An amino acid substitution at the 62nd position (*emf3‐#A62V*) resulted in the FOT group (ii), a shift to later FOT with synchrony (Figure 2d and Tables S1, S2 and S3). Single and double amino acid substitutions at the 104th and 105th positions (*emf3‐#V105I* and *emf3‐#V104I/V105I*) resulted in FOT group (ii), whereas triple amino acid substitutions at the 104th, 105th and 106th positions (*emf3‐#V104I/V105I/E106K*) resulted in the FOT group (iii) (Figure 2d and Tables S1, S2 and S3). These results indicate that genetic manipulation of amino acids in *EMF3* corresponding to *Os03g0145400* leads to wide variation in flower opening time.

### 2.3 *EMF3* Specifically Expressed in Anthers on the Day of Flower Opening

**FIGURE 3:**
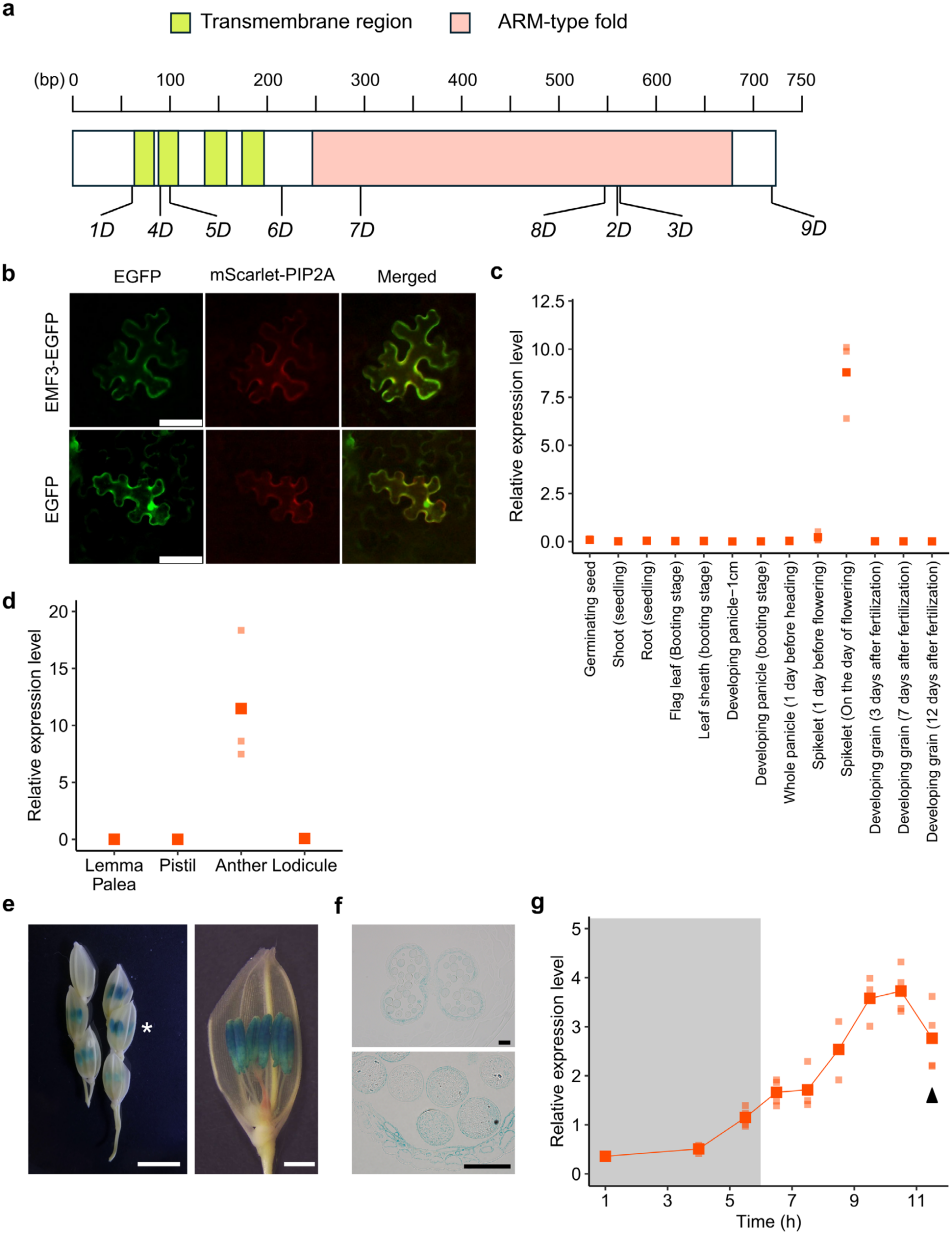
*EMF3* encodes a membrane protein and is specifically expressed in the anther on the day of flowering. (a) Schematic diagram of the primary structure in EMF3 protein. The positions of the single amino acid substitutions of *EMF3* in TILLING mutant lines are shown. (b) Subcellular localisation of the EMF3 protein in *N. benthamiana* leaf epidermal cells. EMF3–EGFP (green) and the plasma membrane marker mScarlet–PIP2A (red) were transiently co‐expressed. Merged images are shown. Scale bars = 40 μm. (c) Quantification of *EMF3* expression levels in various organs of IR64 (wild type) throughout the growth stages. (d) Quantification of *EMF3* expression in spikelet organs of IR64 (wild type) on the day of flowering. (e, f) Histochemical GUS assay of (e) spikelets and (f) transverse section of an anther from transgenic plants expressing the GUS gene driven by the *EMF3* promoter. In e, the spikelet marked with an asterisk in the left panel indicates the spikelet in the right panel. In e, scale bar = 1 cm (left panel) and = 1 mm (right panel); in f, scale bar = 40 μm. (g) Quantification of *EMF3* expression levels in the whole spikelet of genotypes on the day of flowering. In c, d and g, each large symbol represents the mean value from three biological replicates, whereas each small symbol represents the observed raw value in each replicate (*n* = 3 biological replicates). In g, grey and white regions indicate dark and light periods, respectively. Black arrowheads represent the start of flower opening in IR64 at 11:30 under the given condition.

The *EMF3* gene is predicted to have a single exon encoding a polypeptide of 723 amino acids with an armadillo repeat fold and four transmembrane segments (Figure 3a and Table S6). Transient expression of EMF3::EGFP in tobacco cells demonstrated EMF3 protein localisation in the plasma membrane (Figure 3b). BLAST searches identified 30 *EMF3*‐like proteins across 10 species (Table S6). Although grasses and *Marchantia polymorpha* had four or more *EMF3*‐like genes, dicotyledonous plants had fewer (Figure S9a and Table S6). Amino acid substitutions that significantly impacted FOT were not necessarily found at highly conserved residues (Figure S9b).

Throughout wild‐type (IR64) plant development (i.e., from germination to developing grain), *EMF3* was specifically expressed in the anther on the day of flowering (Figure 3c,d). A histochemical GUS assay using rice spikelets on the day of flowering confirmed the specific localisation of EMF3 protein in pollen and the anther‐wall (Figure 3e,f). Ectopic *EMF3* expression in whole‐plant tissues resulted in aberrant shoot and root development from the initial seedling stage, and only a small number of individuals survived until maturity (Figure S10a–f), suggesting the exclusive role of *EMF3* in the anther during flowering events. The FOT in over‐expressed plants that survived was similar to that in wild‐type plants (Figure S10g). We then examined the time‐course of *EMF3* expression in wild‐type (IR64) spikelets on the day of flowering, from 01:00 until flower opening (Figure S11). Expression was incremental during 01:00–04:00 in the dark period (night) but began to increase rapidly after 04:00 (Figure 3g). Expression peaked approximately 2 h earlier than the peak FOT (Figure 3g). Overall, the results suggest the specific localisation of *EMF3* in the anthers on the day of flowering.

### 2.4 *EMF3* Governs Flowering Events in Anthers

**FIGURE 4:**
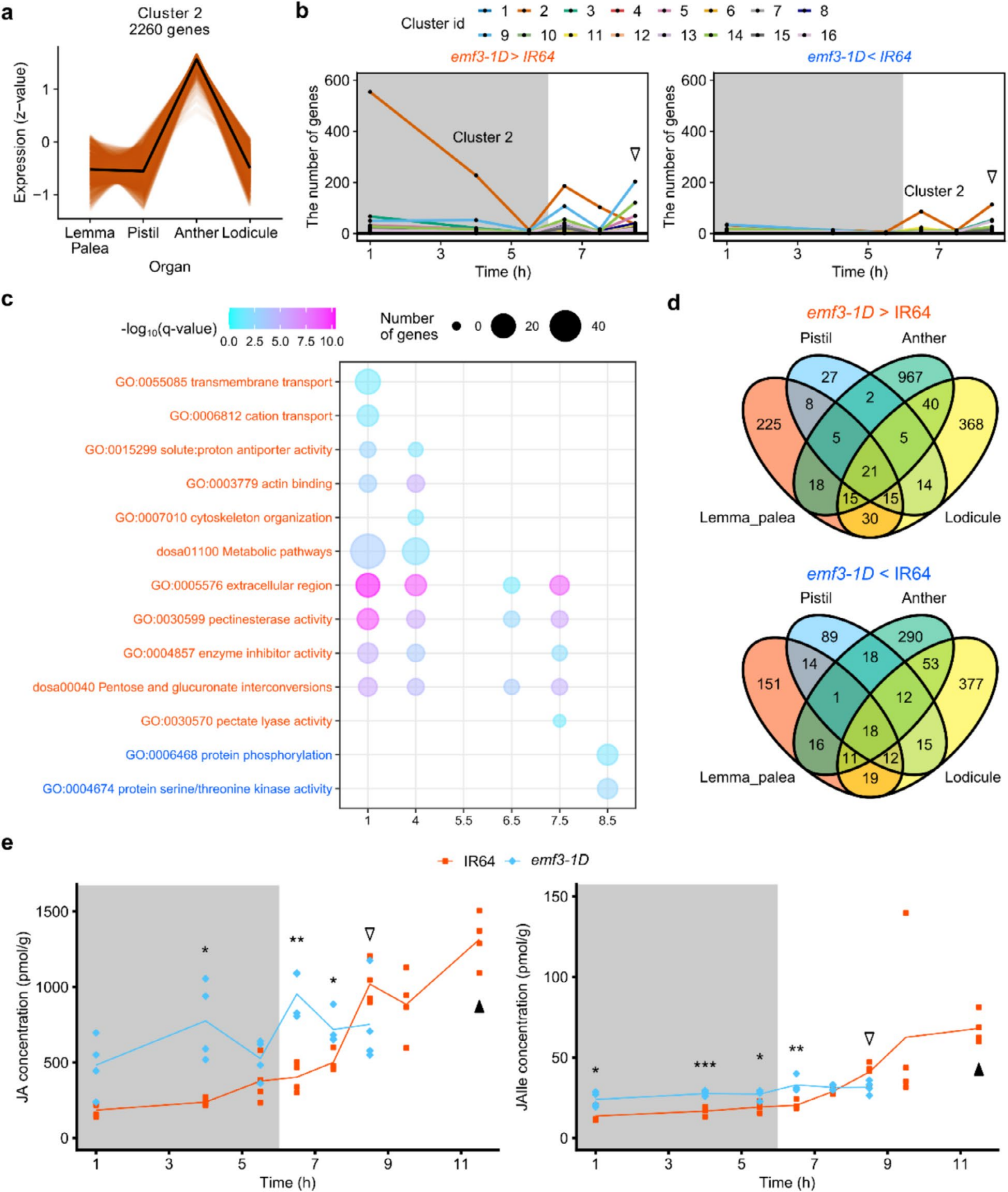
*EMF3* impacts flowering events in the spikelets. (a) Normalised expression levels of 2260 genes classified into Cluster 2, a group of anther‐specific expression genes, in spikelet organs of IR64 on the day of flowering (at 07:30). Orange lines represent the expression of each gene in Cluster 2, whereas the black line represents the mean expression level of the 2260 genes. (b) Number of differentially expressed genes (DEGs) between genotypes from 01:00 to 08:30 in each cluster. DEGs upregulated and downregulated in the *emf3‐1D* allele are shown separately. (c) Gene ontology (GO) and Kyoto Encyclopedia of Genes and Genomes (KEGG) pathways significantly enriched in DEGs between genotypes from 01:00 to 08:30 in Cluster 2. Red and blue text indicate GO terms and KEGG pathways enriched in DEGs that were more highly expressed in *emf3‐1D* and IR64, respectively. The size of the plots represents the number of DEGs, and the colour represents the *q*‐value. (d) Venn diagram showing DEGs in each spikelet organ between genotypes at 07:30 on the day of flowering. DEGs upregulated and downregulated in the IR64 background with the *emf3‐1D* allele are shown separately. (e) Concentration of jasmonic acid (JA) and jasmonic acid‐isoleucine (JA‐Ile) in the spikelets of genotypes. In e, *p < 0.05; **p < 0.01; ***p < 0.001 (Welch's *t*‐test, *n* = 4 biological replicates). In b and e, black and white arrowheads indicate the start of FOT in wild type (IR64) and the *emf3‐1D* allele, respectively, under the given condition.

To investigate the gene expression network associated with early FOT in *emf3‐1D*, we compared spikelet transcriptomes. First, the genes expressed in the spikelets were classified into 16 groups on the basis of their expression in wild‐type (IR64) spikelet organs (the lemma and palea, pistil, anther and lodicule) (Figure S12). Gene ontology (GO) terms and Kyoto Encyclopedia of Genes and Genomes (KEGG) pathway analysis revealed significant enrichment in these clusters, indicating specific gene functions in each cluster (Table S7). *EMF3* was in Cluster 2, which included genes specifically expressed in the anther (Figure 4a). Notably, none of the previously identified genes for FOT (*DFOT1* (Wang et al. 2022), *OsMYB8* (Gou et al. 2024), *OsMYC2* (Zhu et al. 2024), *OsOPR7*, *OsHAN1*, *OsJAZ7* and *OsJAZ9* (Wang et al. 2024)) belonged to Cluster 2 (Table S8). Next, we compared the time courses of the transcriptomes of whole wild‐type and *emf3‐1D* spikelets. Differentially expressed genes (DEGs) between the wild‐type and *emf3‐1D* were extracted and categorised into 16 groups (Figure S12). The highest number of DEGs was observed at 01:00, with most belonging to Cluster 2 (Figure 4b). DEG numbers in Cluster 2 decreased from 01:00 to 05:30, but increased at 06:30. GO and KEGG enrichment analysis of the DEGs in Cluster 2 revealed that genes related to cell osmolality (e.g., solute antiporter activity, GO:0015299) and cell‐wall remodelling (e.g., pectinesterase, GO:0030599; extracellular region, GO:0005576) were significantly upregulated in *emf3‐1D* (Figure 4c). The upregulated genes in *emf3‐1D* showed a consistent pattern (Figure S13). These findings suggest that genes related to cell osmolality and cell‐wall remodelling in the anther were upregulated during the late‐night and early‐morning hours in *emf3‐1D*, contributing to the early FOT.

Lastly, we compared gene expression between wild‐type and *emf3‐1D* spikelet organs at 07:30 to highlight organ‐specific transcriptomic differences (Figures 4d and S12). The number of DEGs was the highest in the anther and was also notably high in the lodicule (Figure 4d). In the anther, genes related to cell osmolality and cell‐wall remodelling were significantly upregulated in *emf3‐1D* (Figure S14 and Table S9), consistent with the results of the whole‐spikelet analysis (Figure 4c). In the lodicule, genes related to water transport (GO:0006833) were upregulated in *emf3‐1D*. Additionally, genes related to translation (GO:0006412) were upregulated in *emf3‐1D*. Collectively, these findings suggest that the transcriptomes of both the anther and lodicule were impacted by the single nucleotide substitution in *EMF3*, aligning with the earlier FOT observed in *emf3‐1D*.

Previous studies on FOT in rice have shown that JA and JA‐Ile are associated with changes in FOT (Ding et al. 2024; Liu et al. 2017; Wang et al. 2024). To investigate this further, we compared JA and JA‐Ile concentrations in wild‐type (IR64) and *emf3‐1D* spikelets (Figure 4e). JA and JA‐Ile levels were consistently higher in *emf3‐1D* than in the wild‐type, particularly during the night. In the wild‐type, an increase in JA and JA‐Ile levels was observed from 04:00 until flowering, whereas this was not clearly observed in *emf3‐1D*. We compared the expression of genes related to JA synthesis and metabolism, which are involved in FOT regulation (Gou et al. 2024; Wang et al. 2024; Zhu et al. 2024) and exhibit anther‐specific expression in Cluster 2 (Figures S15 and S16; Table S8). Among 17 JA‐related genes, 11 exhibited significant expression differences between the wild‐type and *emf3‐1D* (Figures S15 and S16), suggesting the involvement of *EMF3* in JA regulation. Nevertheless, the JA‐related gene expression did not fully explain the higher JA and JA‐Ile content in *emf3‐1D* than in the wild‐type.

### 2.5 Application of *EMF3* Alleles for Rice Breeding

**FIGURE 5:**
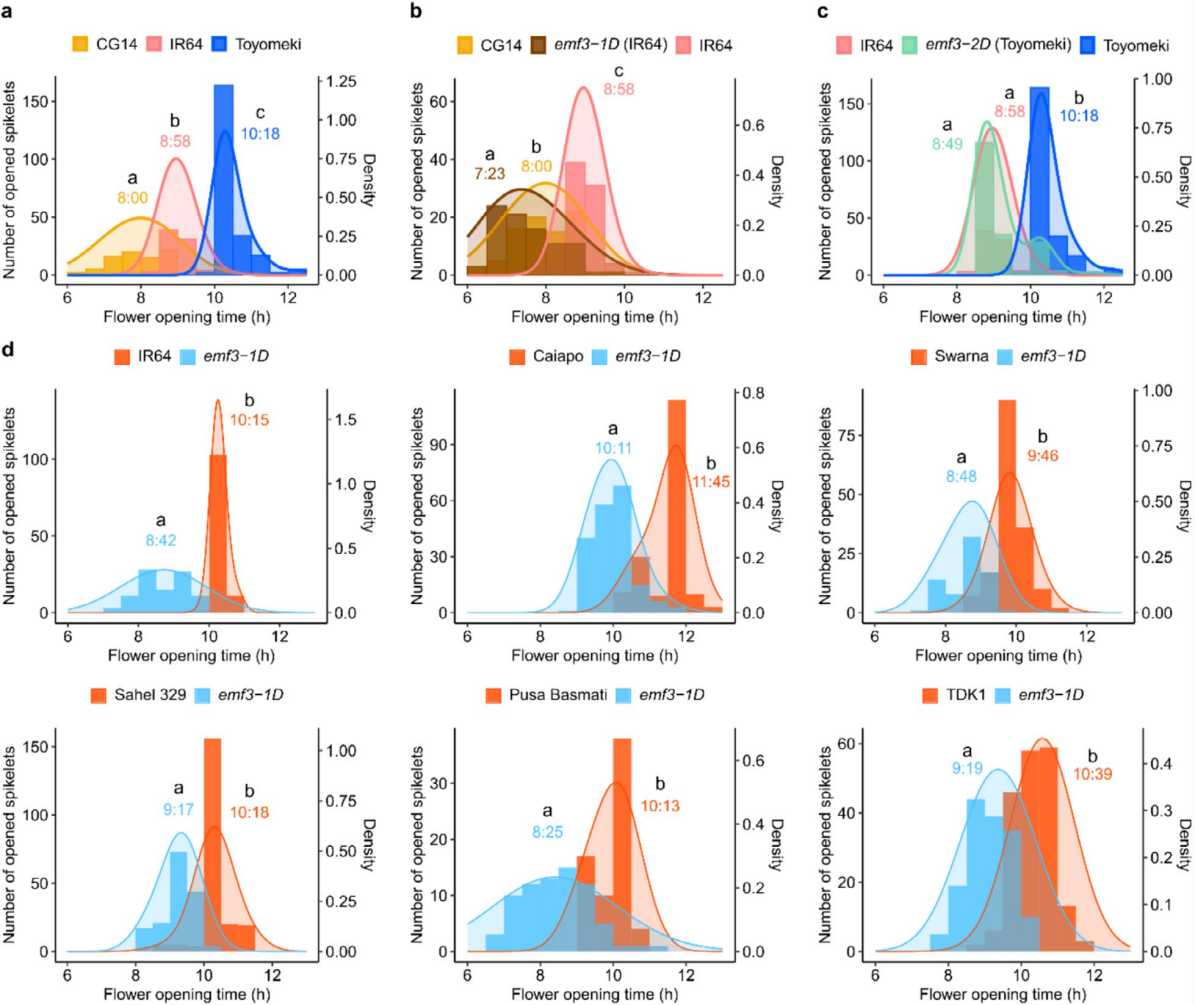
*EMF3* allele(s) facilitate FOT adjustment among rice species and subspecies (a–c) and advances FOT across diverse genetic backgrounds (d). (a) Observation of the FOT of *Oryza sativa* subsp. *japonica* (Toyomeki) and *indica* (IR64), and *Oryza glaberrima* (CG14). (b) Adjustment of FOT of *indica* (IR64) with CG14 using the *emf3‐1D* allele in the IR64 background. (c) Adjustment of FOT of *japonica* (Toyomeki) with *indica* (IR64) using the *emf3‐2D* allele in the Toyomeki background. Note that FOT observation in a–c was conducted on the same day. (d) Observation of FOT by transferring the *emf3‐1D* allele to the genetic background of widely grown tropical cultivars. Different letters indicate statistically significant differences in peak FOT between genotypes, on the basis of Bonferroni‐adjusted 95% confidence intervals calculated using the bootstrap method. Note that the FOT observation in d was conducted on the same day in each genotype.

Previous studies reported that there is little variation in FOT among different rice cultivars (Bheemanahalli et al. 2017; Hirabayashi et al. 2015; Jagadish et al. 2008). The FOT of *Oryza glaberrima* was the earliest, followed by the *indica* cultivar of *O. sativa* . The FOT of the *japonica* cultivar of *O. sativa* was the latest. Thus, the FOTs of these three cultivars (CG14 as representative of *O. glaberrima* African rice cultivar, IR64 as representative of *indica* cultivar and Toyomeki as representative of *japonica* cultivar) were separated with peaks of FOT at 8:00, 8:58 and 10:18, respectively (Figure 5a). Interestingly, the flowering pattern of the IR64 background with the *emf3‐1D* allele shifted closer to that of CG14, although the peak FOT was 37 min earlier in the IR64 background with the *emf3‐1D* allele (7:23) than in CG14 (8:00) (Figure 5b and Tables S1 and S2). In addition, the flowering pattern of the Toyomeki background with the *emf3‐2D* allele shifted to overlap with that of IR64 (Figure 5c and Tables S1 and S2). The *emf3‐1D* and *emf3‐2D* alleles enabled FOT adjustment among *Oryza* species and subspecies. Plant and panicle phenotypes of EMF lines having *emf3‐1D* and *emf3‐2D* alleles were comparable to their wild types, without significant changes in grain yield, yield components and other agronomic traits under normal temperate field conditions (Figure S17). These results demonstrate their suitability for breeding applications to produce the desired changes in FOT.

The *emf3‐1D* allele, which conferred the earliest FOT, was transferred to develop NILs against the diverse genetic backgrounds of several widely grown tropical rice cultivars, including IR64, TDK1 (Laos), Swarna (India), Sahel 329 (West Africa), Caiapo (Brazil) and Pusa Basmati (India), through marker‐assisted selection (Figure S18). Under normal tropical field conditions, all NILs carrying the *emf3‐1D* allele demonstrated significantly earlier FOTs than their recurrent parents (Figure 5d and Tables S1 and S2), demonstrating the availability of *EMF3* for diverse genetic backgrounds.

### 2.6 Heat Escape Test With Early FOT Alleles, *emf3‐1D* and *emf3‐2D*

**FIGURE 6:**
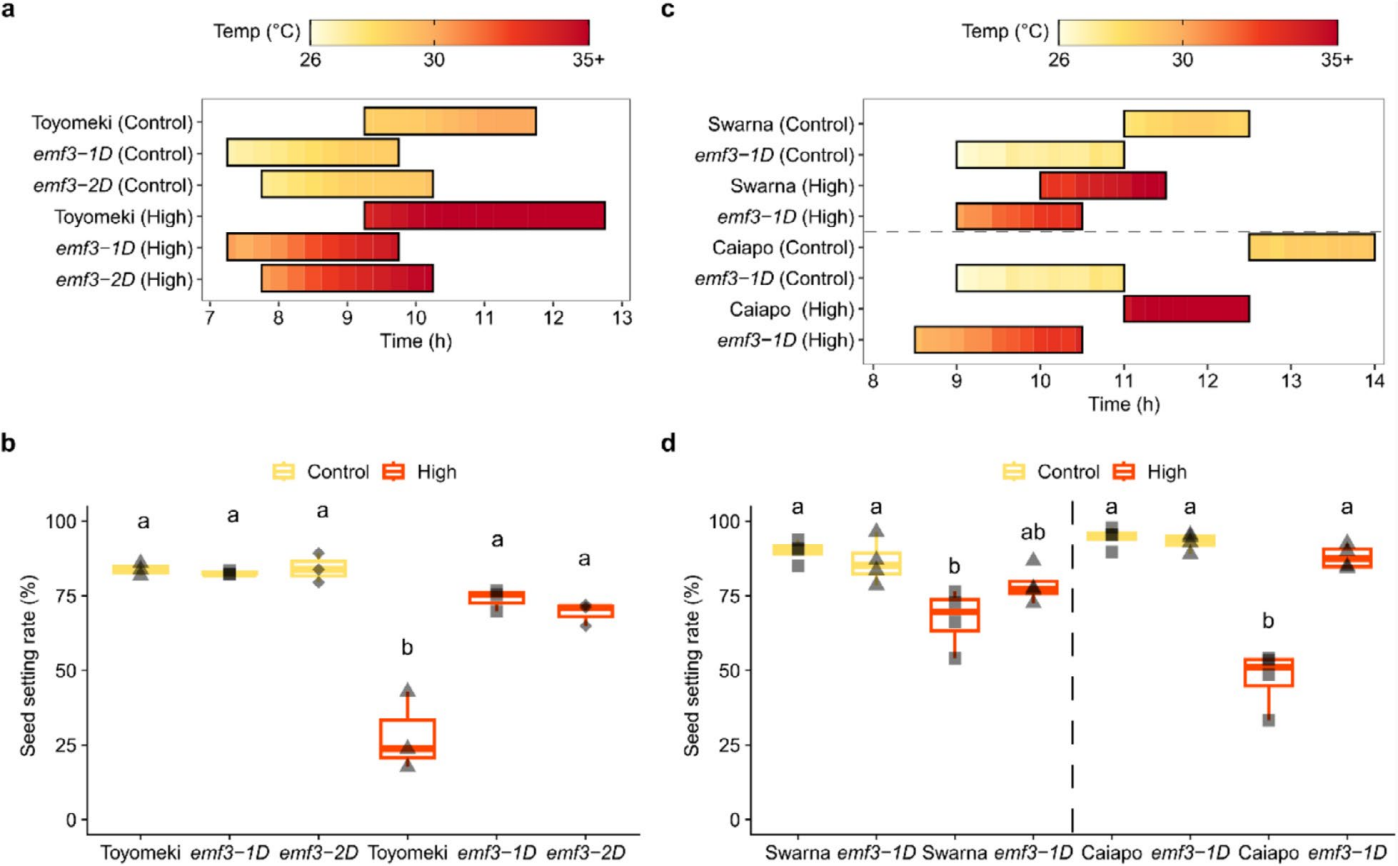
*EMF3* allele(s) mitigate heat‐stress damage in rice genotypes. (a) FOT of the NILs carrying *emf3‐1D* and *emf3‐2D*, and their genetic background (Toyomeki) under outside (control) and temperature‐elevated chamber (high) conditions. (b) Seed setting rate (%) of the NILs carrying *emf3‐1D* and *emf3‐2D*, and their genetic background (Toyomeki) under outside (control) and temperature‐elevated chamber (high) conditions. (c) FOT of Swarna, Caiapo and their NILs carrying *emf3‐1D* under control and temperature‐elevated chamber (high) conditions. (d) Seed setting rate (%) of Swarna, Caiapo, and their NILs carrying *emf3‐1D* under control and temperature‐elevated chamber (high) conditions. In b and d, different letters indicate significant differences at *p* < 0.05 according to Tukey–Kramer's method in each genotype.

Seed setting rate was assessed in temperature‐elevated chamber experiments (up to 38°C) for the *emf3‐1D* and *emf3‐2D* alleles in Toyomeki background (Figure S19). Under both control (normal outdoor temperature) and elevated (high temperature chamber) conditions, the *emf3‐1D* and *emf3‐2D* allele NILs finished flowering at least two hours earlier than wild‐type (Toyomeki). The FOT of the NILs occurred when the temperature in the temperature‐elevated chamber was lower than 35°C (Figure 6a), whereas the spikelets of wild type Toyomeki flowered when the temperature in the temperature‐elevated chamber was higher than 35°C (Figure 6a). Consequently, the seed setting rate of Toyomeki was significantly decreased, whereas seed setting rate of the *emf3‐1D* and *emf3‐2D* allele NILs was retained at a high level in the high temperature condition (Figure 6b).

We further assessed seed setting rate using Swarna, Caiapo and their NILs carrying the *emf3‐1D* allele in temperature‐elevated chamber experiments (up to 37°C). Temperature was linearly elevated starting from 25°C at 6:00 and up to 30°C (control) and 37°C (high temperature) at 12:00 (Figure S19). Under both control (25°C–30°C) and elevated temperature (25°C–37°C) conditions, Caiapo started flower opening after Swarna completed flower opening, whereas FOT of their NILs carrying the *emf3‐1D* allele at least partially overlapped (Figure 6c). Under high temperature, the NILs of Swarna and Caiapo carrying the *emf3‐1D* allele finished flowering when the temperature in the temperature‐elevated chamber was lower than 35°C (Figure 6c, Table S10). On the other hand, the temperature in the temperature‐elevated chamber was around 35°C when spikelets of Swarna finished flowering and was higher than 35°C almost the entire time that spikelets of Caiapo were flowering (Figure 6c, Table S10). Consequently, the seed setting rate was higher in the NILs carrying the *emf3‐1D* allele than in their recurrent parents: *emf3‐1D* exhibited 88.0% seed setting rate compared to 47.4% in Caiapo (*p* < 0.01), whereas *emf3‐1D* showed 78.4% seed setting rate compared to 67.5% in Swarna—not a significant difference (*p* = 0.18) (Figure 6d). These findings suggest that *emf3‐1D* and *emf3‐2D* alleles are promising alleles that can enhance spikelet fertilisation under heat stress conditions, but the magnitude of the gain in seed setting rate for heat escape can vary across different cultivars.

## 3 Discussion

To safeguard global food security, the development of climate‐smart and high‐yielding rice is imperative. One solution that can provide adaptation to heat stress at flowering and a breakthrough technology to facilitate inter‐specific hybrid rice is the modification of flower opening time (FOT) (Hirabayashi et al. 2015; Qian et al. 2016). However, the low genetic variation in FOT among rice cultivars was a bottleneck in mitigating heat‐induced sterility and improving hybrid rice seed productivity. Here, we identified *EMF3*, the causal gene of *qEMF3* (Hirabayashi et al. 2015). Although we aligned the DNA sequences in the TASUKE database (Kumagai et al. 2019), the C‐to‐T substitution at the 181st nucleotide of *Os03g0145400* causing the early morning flowering phenotype (Figure 1d,e) could not be found among popular *O. sativa* cultivars, including parental varieties for a three‐line hybrid rice system (Figures S4 and S5), strongly suggesting that the single amino acid substitution L61F is the allele to critically modify FOT of diverse rice cultivars. The C‐to‐T nucleotide substitution is either from a spontaneous point mutation that occurred during the development of the early morning flowering line or from abnormal recombination because of wide hybridisation between the AA genome (cultivar) and CC genome (*O. officinalis*).

The *EMF3* mutant lines exhibited semi‐dominant inheritance (Figure 1b), leading to diverse phenotypes in FOT and its synchrony (Figures 1f, 2 and S6b,d,f,g). *EMF3* missense mutations were distributed across various regions of the protein, including the transmembrane and armadillo‐repeat domains (Figures 3a and S9). Overexpression of *EMF3* did not affect FOT (Figure S10g), suggesting that the semi‐dominant phenotypes are not caused by gain‐of‐function effects but rather reflect varying degrees of functional impairment of the EMF3 protein. Indeed, when focusing on the homozygous phenotypes, *emf3‐1D*, which maintains a clear synchrony peak with an earlier FOT, carries a missense substitution from leucine to phenylalanine (Table S4)—both hydrophobic amino acids—implying only a modest change in amino acid properties. Similarly, *emf3‐4D*, which maintains a clear synchrony peak with a later FOT, involves a substitution from threonine to serine (Table S4)—both polar, uncharged amino acids—indicating a conservative substitution with only a minor change in side‐chain properties. By contrast, *emf3‐5D*, which loses synchrony and lacks a clear FOT peak, carries a missense substitution from leucine to arginine (Table S4)—replacing a hydrophobic residue with a positively charged one—and is likely to exert a substantial impact on protein structure. Taken together, these observations suggest that alleles causing mild or moderate reductions in EMF3 activity (‘weak’ or ‘intermediate’ alleles) lead to earlier or later FOT synchrony, respectively, while maintaining high seed setting rate, whereas alleles that cause a severe loss of function (‘strong’ alleles) disrupt FOT synchrony and result in reduced seed setting rate (Figures 1f,g, 2a–c and S8).

Interestingly, *EMF3* exhibited semi‐dominant inheritance across all analysed alleles, with heterozygotes showing intermediate phenotypes between the homozygous mutants and the wild type (Figures 1b and 2a–c). In one case, however, *emf3‐3D* showed distinct characteristics, in which homozygotes displayed a later FOT, whereas heterozygotes showed an earlier FOT, as observed in weak alleles. One possible explanation for these observations in heterozygous *emf3* mutants may be that the EMF3 protein forms multimeric complexes, potentially incorporating multiple copies of itself. When functionally impaired EMF3 proteins are incorporated into such complexes, the overall activity of the complex may be reduced, thereby causing alterations in FOT and its synchrony even in heterozygotes. In this study, EMF3 was confirmed to be a membrane‐localised protein (Figure 3b); however, its protein–protein interactions remain unresolved. Future elucidation of EMF3 multimerisation and identification of its interacting protein factors will further facilitate the precise genetic control of FOT in rice.

*EMF3* is specifically expressed in the anthers, categorised into Cluster 2 (Figure 4a), promoting osmotic adjustment and cell‐wall remodelling in the anther (Figure 4b,c). Given its anther‐specific expression and the involvement of water influx in anther dehiscence and lodicule expansion (Hoshikawa 1993; Matsui and Omasa 1999), it is also possible that *EMF3* contributes to the regulation of physiological processes in the anther that are linked to water influx, thereby influencing the timing and synchrony of lodicule expansion at the whole‐spikelet level, without directly functioning in lodicule tissues. Impaired basal anther dehiscence and low FOT synchrony in the *emf3‐KO* mutants generated by CRISPR/Cas9 (Figures 1f,g and S6b,d,f,g) support the specific function of *EMF3* in the anther and its association with coordinated flower opening events. Flower opening in rice is primarily driven by lodicule swelling through water uptake (Hoshikawa 1993), and this biomechanical role of the lodicule is indispensable for spikelet opening; therefore, it remains important to clarify how anthers influence the timing and synchrony of this process. An example of a stamen (anther and filament)‐specifically expressed gene that regulates the timing of flower opening is *DAD1* in *Arabidopsis thaliana* (Ishiguro et al. 2001). *DAD1* is expressed in the filament, and its mutation leads to impaired stamen development and pollen maturation, resulting in delayed flower opening (Ishiguro et al. 2001). These findings indicate that stamens are not merely organs for pollination and fertilisation, but possibly function as a regulatory organ that controls the timing of flower opening. In rice, however, the role of the lodicule in controlling FOT has been the primary focus (Gou et al. 2024; Hoshikawa 1993; Wang et al. 2024, 2022; Xu et al. 2022; Zhu et al. 2024). Previously identified genes, such as *DFOT1* (Wang et al. 2022) and *OsMYB8* (Gou et al. 2024), change FOT by promoting osmotic adjustment and cell‐wall remodelling in the lodicule at flowering. IR64 possesses an *indica* allele of *OsMYB8*, which confers earlier FOT than the *japonica* allele (Gou et al. 2024), but the *emf3‐1D* allele can further advance FOT in the *indica* IR64 genetic background (Figure 1f).

The FOT comparison among *emf3‐1D dfot1*, *emf3‐1D* and *dfot1* (Figure 1j) indicated that *emf3‐1D* is epistatic to *dfot1*, suggesting that *EMF3* functions genetically upstream of *DFOT1* in FOT regulation. These results suggest that both lodicule‐expressed factors and anther‐expressed *EMF3* contribute to FOT control in rice, operating within a coordinated regulatory framework. In this context, *EMF3* is the first gene identified in the anther‐specific Cluster 2 to control FOT, complementing previously identified lodicule‐expressed genes and expanding the regulatory framework of flower opening. In rice, *EMF3* is expressed in anthers specifically on the day of flowering. On the basis of this spatiotemporal expression pattern, we propose that *EMF3* contributes to FOT regulation through anther‐associated physiological signalling that may influence lodicule water uptake, without requiring changes in lodicule anatomical structure. However, the physio‐molecular mechanism underlying *EMF3*‐mediated communication between anthers and lodicules remains unresolved. Structural and functional analyses of *EMF3*, including identification of its interacting factors, will provide important insights into how anther‐derived signals modulate lodicule‐mediated flower opening.

Previous studies reported that an increase in the JA content in the spikelet on the day of flowering advances FOT in rice (Wang et al. 2024). The JA content was higher in the *emf3‐1D* allele than the wild‐type (Figure 4e), in association with an earlier FOT in *emf3‐1D*. This result was consistent with previous studies (Wang et al. 2024). Notably, the higher JA content in the *emf3‐1D* allele was observed particularly during the night on the day of flowering, temporally corresponding to a large number of Cluster 2 differentially expressed genes (DEGs) between the wild‐type and *emf3‐1D* allele during the night (Figure 4b,e). The Cluster 2 DEGs observed during the night contained genes involved in cell osmolality and cell‐wall remodelling (Figure 4c). So far, dynamics of spikelets during nighttime have not been documented to generate genetic variation in FOT. DEGs and JA content were also different between the wild‐type and *emf3‐1D* allele at the beginning of the light period (6:30) (Figure 4b,e). The initiation of the light period in the early morning may make a minor difference in FOT between the wild‐type and *emf3‐1D* allele. Regulatory mechanisms that link *EMF3* alleles with JA content and physio‐molecular dynamics (i.e., changes in protein function) during the night (dark period) and early morning (beginning of light period) need to be further elucidated.

Finally, we demonstrated a breeding strategy for the genetic improvement of hybrid rice seed production and heat stress adaptation by introducing effective *EMF3* alleles into diverse rice cultivars. The *EMF3* alleles, *emf3‐1D* and *emf3‐2D*, which show FOT group (i) with EMF trait (Figures 5b,c and 6b) without yield penalty (Figure S17), will be extremely useful in launching new rice‐breeding programs improving the seed production and adaptation to heat stress of inter‐specific high‐yielding hybrid rice. Originally, the *O. glaberrima* cultivar and the *indica* and *japonica O. sativa* cultivars have different FOTs (Figure 5a). This study demonstrates that the FOT of *Oryza* species and subspecies can be adjusted using *EMF3* alleles (Figure 5b,c). The *emf3‐8D* allele, which shows FOT group (ii) with later FOT (Figure 2b) and comparable seed setting rate with their wild‐types (Figure S8b), is also useful for intra‐specific hybridisation because there is a 1.5 h difference in FOT among popular tropical and subtropical cultivars (Bheemanahalli et al. 2017; Hirabayashi et al. 2015). These three alleles can facilitate a higher chance of fertilisation between parental varieties with wide genetic diversity and different FOTs to exploit heterosis. The use of *EMF3* alleles in hybrid rice will be a breakthrough that reduces the cost of seed production. Because of a semi‐dominant effect, F1 hybrid plants will also possess a partial EMF trait that allows efficient fertilisation under heat stress. Consequently, exploring diverse gene pools in hybrid rice could potentially contribute to breaking the yield barrier. Furthermore, we demonstrate that the *emf3‐1D* and *emf3‐2D* alleles are promising for advancing FOT to escape from heat stress during flowering (Figure 6a,b) and advancement of FOT with the *emf3‐1D* allele is effective in various regional rice cultivars worldwide (Figures 5d and 6c,d). It should be noted, however, that the effectiveness of the *emf3‐1D* and *emf3‐2D* alleles for heat escape will depend on the cultivar. Swarna is an early FOT cultivar with high heat tolerance, whereas Caiapo is a late FOT cultivar with high heat susceptibility, rendering them representative of popular tropical cultivars with contrasting heat adaptations (Hirabayashi et al. 2015; Ishimaru, Hirabayashi, et al. 2016; Shi et al. 2015). The *emf3‐1D* and *emf3‐2D* alleles would drastically improve heat adaptation of genotypes with heat susceptibility and late FOT. In addition, it is known that FOT is determined not only by a genetic factor but also by environmental factors in rice cultivars (Julia and Dingkuhn 2012; Kobayasi et al. 2010). Genetic and environmental interactions need to be clarified to understand how FOT is determined in *EMF3* alleles. Modifying the flower opening time is a unique opportunity to facilitate fertilisation, leading to improved grain and seed production. *EMF3* is a crucial gene in ensuring the high seed setting rate amidst the challenges posed by progressive global warming and feeding the rapidly growing global population.

## 4 Materials and Methods

### 4.1 High Resolution Mapping of the *emf3‐1D* Allele

For high‐resolution mapping, a total of 6110 F_2_ plants were selected from a population derived from a cross between Nanjing11 and the *emf3‐1D* allele (Hirabayashi et al. 2015) as the initial mapping population. An additional 1248 F_2_ plants were selected from a population derived from a cross between IR64 and the *emf3‐1D* allele. The genotype of the *emf3‐1D* allele in each recombinant plant was estimated on the basis of the FOT of the F_3_ or F_4_ progeny.

### 4.2 Identification of Transcription Units Around the Polymorphism

Two transcription units, *Os03t0145300* and *Os03t0145400*, were annotated at the location of the polymorphism that presumably confers the early morning flowering trait according to RAP‐DB (Sakai et al. 2013) (Figure S2b). To identify which transcription units are expressed in spikelets, reverse transcription polymerase chain reaction (RT‐PCR) was performed using primer pairs designed to amplify *Os03t0145300* or *Os03t0145400*. Additionally, a primer pair amplifying a homologue of maize ( *Zea mays* L.) *BT065989*, neighbouring *Os03t0145400*, was also used (Figure S2b,d and Table S11). The RT‐PCR cycling conditions were as follows: denaturation, 98°C; annealing, 58°C; and extension, 72°C for 30 cycles.

### 4.3 Determination of the cDNA Sequence

To determine the full‐length cDNA sequence (Figure S3), rapid amplification of cDNA ends (RACE) was conducted using the SMARTer RACE 5′/3′ Kit (Takara, Shiga, Japan) according to the manufacturer's instructions.

### 4.4 Sequencing of the *EMF3* Gene From *O. officinalis* and Popular Cultivars

Seeds of *O. officinalis* (Accession no: IRGC100947) were obtained from the International Rice Genebank at the International Rice Research Institute (IRRI) in the Philippines. Genomic DNA was extracted from seedling leaves, and PCR was conducted to amplify the full coding sequence of the *EMF3* gene (3′RACE F and 5′RACE R in Table S11). The PCR products were sequenced using the ABI3730XL DNA analyser at Macrogen (Seoul, South Korea). The *EMF3* sequences from the tropics‐adapted temperate *japonica* varieties (MS11, Japonica 1, Cordillera) and popular *indica* varieties (IR8, IR64, Minghui 63, SambaMahsuri) were obtained from the publication (Pacleb et al. 2025) and the NCBI database (https://www.ncbi.nlm.nih.gov/), respectively. The *EMF3* sequences of the other plant materials (IR24, IR56, IR58025B, IR68897B) were extracted from the local nucleotide database (contigs pools generated by using Illumina NGS with de novo sequence assembly) established in the Gene Identification and Validation (GIV) Lab, at IRRI headquarter.

### 4.5 Construction of Plasmids

For GUS expression analyses, the upstream (−3047 to −1) and downstream (+2173 to +4961) regions of *Os03g0145400* were cloned into the binary vector pBIN40 (Yagi et al. 2021), with the *gusA* gene inserted between them, to generate the construct pBIN5400::GUS, which was used for the histochemical GUS assays. For the generation of an over‐expressor (pACT::EMF3), a DNA fragment of the coding region of *EMF3* was inserted into the binary vector pBIAct1‐nos containing the rice *actin 1* promoter (Kamiya et al. 2003). CRISPR/Cas9 vectors were constructed to disrupt *Os03g0145400* using the binary vector pRGEB31 (Xie et al. 2014) (Addgene plasmid 51295). The sgRNA sequence 5′‐ACGGCCGGCTGTACTCCCTCAGG‐3′ was designed to target an exon of *EMF3*. To disrupt *DFOT1* (*Os01g0611000*), a gene known to determine FOT in rice (Wang et al. 2022), the sgRNA sequence 5′‐GTTCAAGGCGAAGATGGACGAGG‐3′ was used. To construct the cytosine base editor targeting *EMF3*, the sgRNA sequences 5′‐CGCCGTCCTCGAGAAGGCGG‐3′, 5′‐CACCACCAGCAGCACGGTGA‐3′ and 5′‐GTGCACCTACGCCGGCGACG‐3′—which were designed with the intention of inducing C‐to‐T substitutions near positions 186, 315 and 1689, respectively—were cloned into the *Bbs*I site of the sgRNA expression vector (Mikami et al. 2015). The SpCas9 in the Target‐AID vector was modified with an amino acid substitution from R1335A to R1335V, and named pZH_ZmUbi_nSpCas9‐NG_AID_UGI (Mikami et al. 2015). The sgRNA cassette was transferred into the AscI/PacI site in pZH_ZmUbi_nSpCas9‐NG_AID_UGI.

### 4.6 Transformation and Generation of Rice Mutants

The binary vector plasmids were introduced into *Agrobacterium tumefaciens* strain LBA4404 by electroporation using an Eppendorf Eporator (Eppendorf, Hamburg, Germany). Transgenic rice plants were generated via *Agrobacterium*‐mediated transformation using either immature embryos (Ishizaki 2016) or calli (Ozawa 2009). The presence of transgenes was confirmed by PCR using the primer pair HPT F and HPT R (Table S11), which amplifies a fragment of the hygromycin phosphotransferase (HPT) gene carried by each binary vector in its T‐DNA regions. The target regions of transgenic *T*_0_ plants generated using the CRISPR/Cas9 vectors were analysed by DNA sequencing. *T*_0_ plants with mutations at the target sites were grown in pots under natural light conditions in a greenhouse at the Japan International Research Center for Agricultural Sciences, Tropical Agriculture Research Front (TARF, JIRCAS) on Ishigaki Island (Okinawa, Japan; 124°1′ E, 24°2′ N). The temperature was controlled at 30°C/25°C (12 h day/12 h night), hereafter referred to as Condition 1, to obtain *T*_1_ seeds. Homozygous *T*_1_ plants with the desired mutations were selected and grown in a greenhouse, and *T*_2_ seeds harvested from the *T*_1_ homozygous mutants were used for further analysis.

### 4.7 Characterisation of Mutant Lines Generated by Genome Editing for Flowering Traits

We characterised the *emf3‐KO#1* mutants, alongside the *emf3‐1D* allele and IR64, using pot tests. Each line was arranged in a randomised complete block design with six replicates and grown under Condition 1. Plants were grown from July 19, 2021, to October 28, 2021. On the day of flowering, pollen maturity was assessed by staining pollen grains in a 1% iodine potassium‐iodide solution for 10 min immediately after pollen shedding onto a slide glass. Stained pollen grains were observed under a microscope and photographed. FOT was monitored every 3 min by capturing images of the flowering panicles. FOT observations for the rest of the mutant lines listed in Table S3 were conducted following the same method. For the *emf3‐KO#1* mutant, *emf3‐1D* allele and IR64, basal anther dehiscence of anthers was measured using a scale loupe (PEAK, 30×) with four individual spikelets from different plants, sampling three anthers per spikelet and seed setting rate was examined by manually counting the filled and unfilled spikelets at maturity.

### 4.8 Detection of *EMF3* Variants, FOT Observation and Yield Determination Using High‐Resolution Melting‐Based TILLING

A mutant population of a *japonica* cultivar, Koshihikari, was generated using the chemical mutagens ethylnitrosourea, methylnitrosourea, sodium azide, double treatment with diepoxybutane and sequential treatment with sodium azide followed by ethyl methanesulfonate. Additionally, from a sodium azide‐treated Toyomeki (a *japonica* cultivar) mutant population, we selected a line carrying a mutation in *Os03g0145400*. *Os03g0145400* DNA fragments were amplified from genomic DNA using PrimeSTAR GXL (TaKaRa) with the primers indicated in Table S5 (TILLING method). A total of 28 mutant lines from the Koshihikari population and seven mutant lines from the Toyomeki population were identified using TILLING with High‐Resolution Melting (HRM) analysis (Dong et al. 2009; Gady et al. 2009; Kuramata et al. 2022). A total of 35 mutant lines carrying a mutation in *Os03g0145400* were used for the first screening of FOT observations (Table S4). For mutant lines with distinct FOT phenotypes, we performed two generations of backcrossing (BC_2_) to their respective background parents (Koshihikari or Toyomeki) to minimise background mutations. SNP markers were developed to identify the mutation site in *Os03g0145400* (Table S5, SNP). FOT observation was conducted in the BC_2_F_2_ generation on potted plants carrying the mutation in *Os03g0145400*. FOT was monitored every 30 min under natural conditions at the NARO Yawara Field in Japan in August of 2023 (140°02′ E, 36°01′ N, 20 m above sea level), following a previously reported method (Hirabayashi et al. 2015). At maturity, the seed setting rate was examined by manually counting the filled and unfilled spikelets at maturity, on the basis of the method of Hirabayashi et al. (2015). Note that the FOT of *O. glaberrima* (CG14, African rice cultivar), an *indica* cultivar of *O. sativa* (IR64) and a *japonica* cultivar of *O. sativa* (Toyomeki) were also monitored in August of 2024, together with that of the mutants carrying *EMF3* alleles.

Yield and yield components for the *emf3‐1D* and *emf3‐2D* alleles were determined growing rice plants under natural field conditions at the NARO Yawara Field in Japan in 2024 (140°02′ E, 36°01′ N, 20 m above sea level) with 8.0 g m^−2^ of nitrogen fertiliser application. Three independent plots per genotype were arranged in a randomised complete block design, with 12 plants in a row per plot. The average of maximum air temperatures during the flowering stage was 33.6°C for the Koshihikari background and 35.3°C for the Toyomeki background. At maturity, the centre six of 12 plants were used for measuring agronomic traits, then harvested per plot for the determination of grain yield and yield components.

### 4.9 Phylogenetic Analysis and Sequence Alignment of EMF3 Homologues

A phylogenetic tree of the *EMF3* gene was constructed using MEGA11 (Tamura et al. 2021). A neighbour‐joining tree was inferred from the amino acid sequences of EMF3 homologues obtained from the National Center for Biotechnology Information (Table S6). Bootstrap analysis was performed with 1000 replicates to assess branch support. Amino acid sequence alignments were conducted using GENETYX‐MAC Ver.19.

### 4.10 Subcellular Localisation of EMF3 in Plant Cells

The transmembrane domains of the EMF3 protein were predicted using the SOSUI membrane protein prediction programme (Hirokawa et al. 1998) (https://harrier.nagahama‐i‐bio.ac.jp/sosui/mobile/). The ORF of the *EMF3* gene without the stop codon was fused to the EGFP expression vector under the Arabidopsis *UBQ10* promoter to form an EMF3‐EGFP fusion protein. As a plasma membrane marker, a vector expressing PIP2A fused to mScarlet under the control of the Arabidopsis UBQ10 promoter was also used (Cutler et al. 2000). The plasmid and pBICp19 expressing the silencing suppressor p19 were then transformed into the *A. tumefaciens* strain EHA105 for transient expression in *N. benthamiana* leaf epidermal cells (Takeda et al. 2002; Wydro et al. 2006). The EGFP signals were observed using a fluorescence microscope (Nikon model Eclipse Ni‐U, Nikon, Japan). Histochemical assay of GUS activity in floral tissues of pBIN5400::GUS (Taichung 65) lines was performed according to the standard staining protocol after fixation in 90% acetone (Takada et al. 2001). For histological sections, stained spikelets were fixed overnight at 4°C in 50% ethanol, 5% acetic acid and 3.7% formaldehyde, embedded in Technovit 7100, and cut with a microtome.

### 4.11 qRT‐PCR Analysis

For qRT‐PCR analysis of various organs throughout the growth stages, and of organ‐specific expression in rice spikelets on the day of flowering, rice plants were grown under naturally illuminated conditions at a constant 25°C (hereafter referred to as Condition 2). For expression analysis of the various organs throughout the growth stages, each organ was immersed in liquid nitrogen and then stored at −80°C until extraction. For organ‐specific expression analysis in rice spikelets on the day of flowering, the spikelets were sampled at 08:30 for the IR64, 1.0–1.5 h prior to flower opening. Spikelets were manually dissected into the lemma and palea, pistil, anther and lodicule on dry ice using sharp forceps, and the samples were stored at −80°C until extraction.

For the time‐course expression analysis of *EMF3*, plants were grown in a chamber (1.2 × 1.4 m; Koito Kogyo, Japan) maintained at 25°C, with lighting from 06:00 to 18:00 using red‐blue Flower Plant Growth LEDs (Apollo 20, Shenzhen, China) at a photosynthetic photon flux density of 1100 μmol m^−2^ s^−1^ at panicle height (hereafter referred to as Condition 3). FOT observations were recorded manually at 30‐min intervals, following a previously reported method (Hirabayashi et al. 2015). Spikelets were sampled at 1–3‐h intervals on the day of flowering, starting at 01:00 (dark conditions) until the beginning of FOT (light conditions) for each genotype (Figure S11). Sampled spikelets were stored at −80°C until extraction.

For prepared samples from Conditions 2 and 3 above, total RNA was extracted using an ISOSPIN Plant RNA kit (Nippon Gene, Tokyo, Japan), and reverse transcription was performed using a PrimeScript Reverse Transcription reagent kit with a gDNA eraser (Takara Bio Inc., Shiga, Japan). Transcript quantification by real‐time PCR was conducted using the Thermal Cycler Dice Real Time System II (Takara Bio Inc.) with TB Green Premix Ex Taq II (Takara Bio Inc.) according to the manufacturer's instructions. The primer pairs for *Os03g0145400* (5400_P10_F and 5400_P10_R) and *DFOT1* (DFOT1_F and DFOT1_R) are listed in Table S11. Data were obtained from three to four biological replicates by being normalised with 18S rRNA (18S_F and 18S_R).

### 4.12 Statistical Analyses

Data were analysed using the Tukey–Kramer test, Welch's *t*‐test, bootstrap test for differences in the peak position of probability density functions with Bonferroni correction, all performed using R (R Core Team 2024) version 4.4.1. The significance levels considered in this study were 95% and 99%.

### 4.13 RNA‐Seq Analysis

For time‐course transcriptome analysis of whole spikelets in IR64 and the *emf3‐1D* allele with IR64 background, spikelets sampled under Condition 3 were used. For organ‐specific transcriptome analysis of rice spikelets in IR64 and the *emf3‐1D* allele, plants were grown under Condition 3, and the spikelets were sampled at 07:30 for both lines (Figure S11). The spikelets were manually dissected into the lemma and palea, pistil, anther and lodicule on dry ice using sharp forceps. RNA extraction was performed on three or four biological replicates.

Total RNA was extracted as described for the qRT‐PCR analysis. For each sample, 500 ng of total RNA was used as the input for library preparation. RNA sequencing libraries were prepared using Lasy‐Seq (Kamitani et al. 2019) version 1.1 (https://sites.google.com/view/lasy‐seq/). Sequencing was performed using the NovaSeq X Plus (Illumina, San Diego, CA, USA) at Macrogen (Seoul, South Korea) with paired‐end sequencing lengths of 150 bp. Only Read 1 was used for subsequent analysis.

The obtained reads were trimmed using fastp version 0.20.1 (Chen et al. 2018) with the following parameters: ‐t 51, ‐q 20, ‐l 50, ‐‐trim_poly_x and ‐‐adapter_fasta. The adapter sequences used for the ‐‐adapter_fasta option were 5′‐AGATCGGAAGAGCACACGTCTGAACTCCAGTCA‐3′ and 5′‐AGATCGGAAGAGCGTCGTGTAGGGAAAGAGTGT‐3′. The trimmed reads were then mapped onto the reference sequences of the IRGSP‐1.0 (2023‐09‐07) (Kawahara et al. 2013), the mitochondrial genome (NC_001320.1), the chloroplast genome (NC_011033.1) and virus reference sequences, which consisted of the complete genome sequences of 7457 viruses obtained from NCBI GenBank (Kashima et al. 2021). Mapping was performed using RSEM version 1.3.3 (Li and Deway 2011) and Bowtie2 version 2.5.1 (Langmead and Salzberg 2012) with default parameters.

All statistical analyses for the RNA‐Seq data were performed using R software version 4.4.1 (R Core Team 2024). Counts per million (cpm) were calculated using the nuclear‐encoded gene raw count data, excluding genes encoding rRNA. The effects of misassigned reads in multiplex sequencing experiments were corrected as previously described (Kashima et al. 2021), resulting in 1.7–18.1 million reads per sample (Figure S20a). A total of 19 275 genes, with average mapped reads > 10, were used for cpm calculation (Figure S20b). The cpm was calculated using TMM normalisation with edgeR (Robinson et al. 2010). Log_2_cpm values were calculated as log_2_ (cpm +1).

*K*‐means clustering analysis was conducted using the *k*‐means function with default parameters in R. DEG analysis was performed using the R package TCC, version 1.32.0 (Sun et al. 2013; Tang et al. 2015). Normalisation was conducted using iDEGES/edgeR (Robinson et al. 2010) with a false discovery rate (FDR) of 0.1, and DEG detection was conducted using edgeR, setting FDR = 0.05. Venn diagrams were drawn using the R package ggvenn version 0.1.10. (Yan 2023). Gene enrichment tests for Gene Ontology (GO) and Kyoto Encyclopedia of Genes and Genomes (KEGG) (Kanehisa and Goto 2000) pathway analyses were performed using the R package GO.db version 3.19.1 (Carlson 2024) and KEGGREST version 1.32.0 (Tenenbaum and Bioconductor Package Maintainer 2024), respectively, following previously described methods (Nagano et al. 2019). FDR was controlled using the Benjamini and Hochberg method (Benjamini and Hochberg 1995), with FDR = 0.05.

### 4.14 Jasmonic Acid Quantification

Spikelets of IR64 and the *emf3‐1D* allele sampled under Condition 3 were used. Three or four spikelets per time point were harvested and freeze‐dried under a vacuum for 5 days. After measuring the dry weight, the freeze‐dried samples were pulverised using a TissueLyser II (QIAGEN). Extraction and quantification of phytohormones were conducted following previously described methods (Kojima et al. 2009; Kojima and Sakakibara 2012). Endogenous JA and JA‐Ile were quantified using ultra‐high‐performance liquid chromatography with an ODS column (ACQUITY Premier HSS T3 with VanGuard FIT, 1.8 μm, 2.1 × 100 mm; Waters), coupled with a Q‐Exactive mass spectrometer (ThermoFisher Scientific, USA), without MS probe modification. The resulting data were processed using XCALIBUR 4.5 software (Thermo Fisher Scientific, USA).

### 4.15 Time Course of Lodicule Weights

Lodicules of IR64 and the *emf3‐1D* allele with the IR64 background sampled under Condition 2 were used. Five lodicules were collected per panicle, and the fresh weight of the lodicules was measured with a precision balance.

### 4.16 Development of Near‐Isogenic Lines (NILs) With the *emf3‐1D* Allele in Regional Popular Rice Cultivars

The *emf3‐1D* allele NILs with Nanjinn 11 and IR64 background were derived from Hirabayashi et al. (2015), whereas the *emf3‐1D* allele NILs with Koshihikari and Toyomeki background were derived from Hirabayashi et al. (2023). The *emf3‐1D* allele with IR64 background (Hirabayashi et al. 2015) was used as the donor parent for the EMF trait. Backcrossing was continued until BC_5_F_1_ plants were obtained for TDK1 and Swarna, and BC_4_F_1_ plants for Sahel 329, Caiapo and Pusa Basmati. Candidate NILs carrying donor segments containing the *emf3‐1D* allele were selected through genotyping of BC_5_F_2_ or BC_4_F_2_ using 384 SNP genotyping (Thomson et al. 2012). The flanking markers used to identify the segments containing the *emf3‐1D* allele were 2534594 and 2538347 for TDK1, Swarna, Sahel 329 and Pusa Basmati, but 2532869 and 2544876 for Caiapo. Graphical genotypes of each NIL carrying the *emf3‐1D* allele are shown in Figure S18. FOT was monitored every 30 min in the flooded paddy fields at the headquarters of the IRRI (Los Baños, Laguna, Philippines, 121°2′ E, 14°1′ N, 22 m above sea level), which has a tropical climate.

### 4.17 Heat Escape Test Under Elevated High Temperatures

The *emf3‐1D* allele, *emf3‐2D* allele and their wild‐type (Toyomeki) were grown outdoors in six pots per genotype, and half the number of pots were transferred to the naturally‐illuminated temperature‐elevated chamber at flowering. The temperature inside the chamber was set to linearly increase from 28.0°C at 6:00 up to 38°C at noon (Figure S19). FOT was monitored every 30 min under the outdoors and the temperature‐elevated chamber. After the temperature treatment, all pots were placed outdoors. Seed setting rate was examined by manually counting the filled and unfilled spikelets at maturity.

Swarna, Caiapo and their NILs with the *emf3‐1D* allele were tested under light‐controlled elevated high temperatures at flowering. On the day of flowering, the temperature was set to 25°C until 06:00, after which it increased linearly to reach 30.0°C (control) and 37.0°C (high temperature) at noon (Figure S19). The maximum temperature in each treatment was maintained until 14:00, then decreased linearly to reach 25.0°C by 20:00. The FOTs were visually monitored at 30‐min intervals to check the start, peak and end of FOT. After temperature treatment, the pots were returned to a constant temperature of 25°C. Seed setting rate was examined by manually counting the filled and unfilled spikelets at maturity.

## Author Contributions

T. Ishimaru conceived and supervised the project and performed the expression analysis and physiological observations. T. Ishizaki and H.T. conducted the genetic analysis using transgenic and CRISPR/Cas9‐mutated materials. Y.H. and A.J.N. carried out the transcriptome analysis. K. Sasaki and H.H. conducted fine‐mapping. K. Sugimoto selected the *EMF3* mutant lines, and H.H. and M.W. observed their FOT. K.‐I.H. constructed the vectors, conducted protein subcellular localisation experiments with R.M. and conducted the over‐expression experiment. K. Sasaki, K.‐I.H. and S.‐R.K. performed sequence analysis. H.S., M.K. and Y.T. measured the hormone content. M.J.T. selected NILs carrying the *emf3‐1D* allele, and E.V.M.S.‐A., T.T. and H.S. observed the flowering pattern. K.‐I.H. supervised the genetic experiments. T. Ishimaru, K.‐I.H., T. Ishizaki, H.H., Y.H., K. Sasaki and H.T. wrote the manuscript with feedback from all authors.

## Funding

This work was supported by the Japan Science and Technology–Mirai Program, Japan (grant number: JPMJMI12212), JSPS KAKENHI (grant numbers: JP15K18625 and JP18H02191) and MOFA and MAFF, Japan—Climate Change Adaptation for Rainfed lowland Area (CCARA).

## Conflicts of Interest

The authors declare no conflicts of interest.

## Supporting information


**Figure S1:** Quantitative comparison of fresh lodicule weights between genotypes at different time points. Each large symbol represents the mean value from three biological replicates, whereas each small symbol represents the observed raw value in each replicate. Sampling time points were at 05:00 (30 min before sunrise), 06:30 (1 h after sunrise), 08:00 (2.5 and 1 h before peak flower opening time (FOT) in IR64 and the *emf3‐1D* allele, respectively), 09:00 (1.5 h before peak FOT in IR64 and at peak FOT in the *emf3‐1D* allele), 09:30 (30 min after peak of FOT in the *emf3‐1D* allele), 10:30 (peak FOT in IR64), 11:00 (30 min after peak FOT in IR64).
**Figure S2:** Identification of transcription units around the polymorphism conferring the early morning flowering trait. (a) Graphical genotype of recombinant plants near the *emf3‐1D* allele. White bars represent the background cultivar genotypes and black bars represent the *emf3‐1D* allele genotypes. DNA markers with physical positions on chromosome 3 are shown in the column, whereas genotypes and flower opening time (FOT) phenotype are shown in the left and right rows, respectively. (b) Annotations surrounding the polymorphism responsible for the early morning flowering trait. Arrows with numbers (e.g., 5300 F1) indicate the primer binding sites used in RT‐PCR, with the sequence of each primer provided in Table S11. The location of the polymorphic site is also indicated. (c) RT‐PCR detection of transcripts for candidate genes around the polymorphic site. Genes include 5898 (a homologue of maize gene *BT065989*), 5300 (*Os03t0145300*), 5400 (*Os03t0145400*). *Actin 1* (*Os03g0718100*) was included as a control. Genomic DNA and cDNA synthesised from RNA extracted from the panicle were used as templates. (d) Identified transcription units near the polymorphic site and the structure of *Os03t0145400*. Arrows represent the binding sites of primers used for RT‐PCR.
**Figure S3:** Nucleotide and amino acid sequences of *Os03g0145400*. (a) The nucleotide sequence of *Os03g0145400* cDNA is shown, with the CDS region highlighted in red. (b) The predicted amino acid sequence of *Os03g0145400*. Full‐length cDNA was obtained by RACE using gene‐specific primers 3′RACE F and 5′RACE R (Table S11).
**Figure S4:** Nucleotide sequence comparison of the *EMF3* coding region. IRGC100947 is the accession of *O. officinalis* (the donor of the early morning flowering trait). Only *emf3‐1D* shows the single C‐to‐T nucleotide substitution at the 181st position of the *EMF3* gene (highlighted in red), which is responsible for the early morning flowering trait. Note that the genome sequence of two B lines (maintainer line: IR58025B & IR68897B) is the same as that of A lines (male sterile lines; IR58025A & IR68897A) except for the mitochondrial genome in the three‐line hybrid system. The coding region of *EMF3* was amplified using gene‐specific primers 5400_F0_i, 5400_1111R_i, 5400_923F_i and 5400_11R_i (Table S11).
**Figure S5:** Amino acid sequence comparison of the *EMF3* gene. IRGC100947 is the accession of *O. officinalis* (the donor of the early morning flowering trait). Only *emf3‐1D* shows the single L‐to‐F amino acid substitution at the 61st position in *EMF3* (highlighted in red), which is responsible for the early morning flowering trait.
**Figure S6:** FOT and plant appearance in *EMF3* knockout (KO) mutants. (a) DNA sequences of mutated *EMF3* in *emf3‐KO#1* and *emf3‐KO#2* mutants generated by CRISPR/Cas9. (b) Observation of FOT for *emf3‐KO#2* mutants (the *emf3‐1D* allele in an IR64 background), IR64, and *emf3‐1D*. FOTs of *emf3‐KO#1* are shown in Fig. 1f. (c) DNA sequences of mutated *EMF3* in *emf3‐KO#3* mutants, generated by CRISPR/Cas9. (d) Observation of FOT for the *emf3‐KO#3* mutant (IR64 background), IR64, and *emf3‐1D*. (e) DNA sequences of mutated *EMF3* in *emf3‐KO#4* and *emf3‐KO#5* mutants, generated by CRISPR/Cas9. (f) Observation of FOT of the *emf3‐KO#4* mutant (NERICA1 background) and NERICA1. (g) Observation of FOT of the *emf3‐KO#5* mutant (Nipponbare background) and Nipponbare. In each panel, observation of FOT was conducted on the same days among genotypes. (h) Appearances of IR64 (left), *emf3‐1D* (center), and *emf3‐KO#1* (right) plant at maturity. In (a) (c) and (e) the underlined sequences indicate the PAM regions. Red letters indicate inserted nucleotides and red dashes indicate deleted nucleotides. In (b) (d) (f) and (g) the histograms represent the raw data of number of opened spikelets at each time point based on observations. The shaded areas indicate the estimated probability density. The times indicated in each panel represent the peak FOTs, estimated from the probability density. Different letters indicate statistically significant differences in peak FOT between the genotypes, on the basis of Bonferroni‐adjusted 95% confidence intervals calculated using the bootstrap method.
**Figure S7:** Expression analysis of *DFOT1* and DNA sequences of mutated *DFOT1*. (a) Quantification of *DFOT1* expression levels in various organs of IR64 throughout the growth stages. (b) Quantification of *DFOT1* expression in spikelet organs on the day of flowering. In (a), (b), each large symbol represents the mean value from three biological replicates, whereas each small symbol represents the observed raw value in each replicate. (c) DNA sequences of mutated *EMF3* generated by CRISPR/Cas9 in the *dfot1* and *emf3‐1D doft1* mutants. The underlined sequences indicate PAM regions and the red dashes mark deleted nucleotides.
**Figure S8:** Seed setting rate in TILLING mutant lines. (a) Seed setting rate of mutants which belong to group (i) exhibiting shift to earlier FOT with synchrony. (b) Seed setting rate of mutants which belong to group (ii) exhibiting shift to later FOT with synchrony. (c) Seed setting rate of mutants which belong to group (iii) exhibiting low FOT synchrony. Different letters indicate significance at the 1% level according to Tukey's method. Each boxplot consists of 4–6 biological replicates, whereas each small symbol represents the observed raw value in each replicate.
**Figure S9:** Phylogenetic tree and sequence alignment analysis of EMF3. (a) Phylogenetic tree of the *EMF3* gene constructed using MEGA (Tamura et al. 2021). A neighbour‐joining tree was inferred from the amino acid sequences of *EMF3* homologues obtained from NCBI (Table S6). Bootstrap values were calculated on the basis of 1000 replicates. The scale bar indicates genetic distance on the basis of branch length. (b) Amino acid sequence alignment of EMF3 and its homologues from rice and Arabidopsis. Fully conserved residues and those matching the majority consensus are highlighted in black and grey boxes, respectively. Predicted transmembrane domains and ARM repeat domain are indicated above the alignment with green and pink lines, respectively. Red triangles denote the sites of mutation identified in the nine *emf3* alleles.
**Figure S10:** Effects of *EMF3* overexpression on plant development in transgenic lines. (a) Relative expression levels of *EMF3* in pACT::empty (negative control) and pACT::EMF3 lines (#1 and #2). Expression levels were determined by real‐time RT‐PCR, with 18S rRNA used for normalisation. Data represent means ± SD. (b–d) Seedling phenotypes at 9 days after germination in pACT::empty (b), pACT::EMF3 #1 (c) and pACT::EMF3 #2 (d). pACT::EMF3 #2 (d) showed slow growth. Scale bars: 1 cm. (e) Morphology of plants at 51 days after germination. Arrowheads indicate individuals who have died. Although the majority of overexpression lines exhibited lethality, a small number of individuals survived until maturity. Scale bars: 5 cm. (f) Appearance of rice plants at 2–3 weeks after heading. Scale bars: 12 cm. (g) FOT observed in pACT::empty and pACT::EMF3 #1 lines that survived until maturity.
**Figure S11:** Sampling scheme for transcriptome analysis of spikelets of IR64 and *emf3‐1D* on the day of flowering. The sampling times for each genotype and the types of samples collected are illustrated. Grey and white bars indicate dark and light conditions, respectively. Flower opening begins at 11:30 for IR64 and 8:00 for *emf3‐1D*. Time during 18:00–0:00 is the day before the day of flowering.
**Figure S12:** *K*‐means clustering of genes expressed in spikelet organs, including the lemma and palea, pistil, anther and lodicule, of IR64 at 07:30 (light conditions). The genes were grouped into 16 clusters. The normalised expression (*z*‐value) of each gene in each cluster is shown as coloured lines, whereas the mean normalised expression level of the genes in each cluster is represented by the black line.
**Figure S13:** *K*‐means clustering of genes in Cluster 2. (a) *K*‐means clustering of 2260 genes in Cluster 2 (from Figure S12) on the basis of expression patterns in spikelets of IR64 from 01:00 to 11:30. The genes were classified into six groups. The normalised expression (*z*‐value) of each gene in each group is shown by the coloured lines, whereas the mean normalised expression level of the genes in each group is shown by the black line. (b) Classification of differentially expressed genes (DEGs) in spikelets between IR64 and the *emf3‐1D* allele into six groups, on the basis of the clustering in (a). The normalised expression of each gene is represented by the red and blue lines for IR64 and the *emf3‐1D* allele, respectively.
**Figure S14:** GO and KEGG pathways significantly enriched in DEGs between genotypes at 07:30 in the anther and lodicule. The size of the plots represents the number of DEGs, and the colour represents the *q*‐value.
**Figure S15:** Expression of JA‐related genes in spikelets and spikelet organs of IR64 and *emf3‐1D*. Asterisks indicate significant differences in expression (fold change > 1.5, FDR = 0.05) between the genotypes. *n* = 3–4 (biological replicates). Genes with significant expression differences between the wild‐type and *emf3‐1D* are indicated in red.
**Figure S16:** Expression of JA‐related genes belonging to Cluster 2 in spikelets and spikelet organs of genotypes. The expression of *JAR1*, a member of Cluster 2, is not shown in this figure but is provided in Figure S15c. Asterisks denote significant differences in expression (fold change > 1.5, FDR = 0.05) between the genotypes. *n* = 3–4 (biological replicates). Genes with significant expression differences between the wild‐type and *emf3‐1D* are indicated in red.
**Figure S17:** Evaluation of yield, yield components and agronomic traits in *emf3‐1D* and *emf3‐2D* of Toyomeki background in the normal temperature field condition. (a) view of the demonstration plot and panicle phenotype at maturity. (b) yield, yield components and agronomic traits, *n* = 3 plots (six plants were harvested per plot). Error bars indicate SD. Different letters indicate significant differences at *p* < 0.05 according to Tukey–Kramer's method.
**Figure S18:** Graphical genotype of near‐isogenic lines (NILs) with the *emf3‐1D* allele against the genetic background of widely grown tropical rice cultivars. White bars represent cultivar background segments, whereas black bars represent segments containing the *emf3‐1D* allele on chromosome 3. Grey bars in the *emf3‐1D* allele with the Caiapo background represent segments from the IR64 donor parent with the *emf3‐1D* allele.
**Figure S19:**. Actual temperature at the heat escape tests. (a) Test for *emf3‐1D* and *emf3‐2D* alleles in Toyomeki genetic background. (b) Test for *emf3‐1D* allele in Swarna or Caiapo genetic background.
**Figure S20:** RNA‐Seq data preprocessing. (a) Histogram of the total read counts for each sample, coloured by sample type. (b) Histogram of the mean read count for each gene. The red line represents the mean read count = 10, the threshold for genes used in the analyses, with those above it considered expressed. After filtering, 19 275 genes were used for the analyses.

**Table S1:** Peak FOT estimated from the probability density of FOT.
**Table S2:** Differences by genotypes in peak FOT estimated from the probability density of FOT.
**Table S3:** Mutant lines generated by genome editing.
**Table S4:** Single amino acid mutation lines and FOT by each genotype in BC_2_F_2_ generation of the TILLING mutant lines.
**Table S5:** Primer list for selecting single nucleotide substitution lines in TILLING mutant panels.
**Table S6:** List of *EMF3* and *EMF3* ‐like gene family members in different plants.
**Table S7:** Enriched gene ontology and KEGG pathway in each clusters.
**Table S8:** Clustering of jasmonate‐related genes.
**Table S9:** Enriched gene ontology and KEGG pathway in DEGs between IR64 and *emf3‐1D* in each spikelet's organ at 7:30.
**Table S10:** Flower opening time of NILs carrying *emf3‐1D* allele in the heat escape test.
**Table S11:** Sequences of primers used in this study.

## Data Availability

The RNA‐Seq datasets generated and/or used in this study are available from the Sequence Read Archive (SRA) under accession number PRJNA1158204. *Code Availability*: The scripts used in this study are available from Zenode (Hashida 2026).
