# Supplementary material for: Rice EMF3 Alleles Adjust Flower Opening Time to Enhance the Seed Setting Rate Under High Temperature Stress
Source: Plant Biotechnol J. 2026 Apr 9;24(8):4780–98. doi: 10.1111/pbi.70653 (PMC13387886; doi:10.1111/pbi.70653)
Supplement: Supplementary file 1 — Figure S1: Quantitative comparison of fresh lodicule weights between genotypes at different time points. Each large symbol represents the mean value from three biological replicates, whereas each small symbol represents the observed raw value in each replicate. Sampling time points were at 05:00 (30 min before sunrise), 06:30 (1 h after sunrise), 08:00 (2.5 and 1 h before peak flower opening time (FOT) in IR64 and the emf3‐1D allele, respectively), 09:00 (1.5 h before peak FOT in IR64 and at peak FOT in the emf3‐1D allele), 09:30 (30 min after peak of FOT in the emf3‐1D allele), 10:30 (peak FOT in IR64), 11:00 (30 min after peak FOT in IR64). Figure S2: Identification of transcription units around the polymorphism conferring the early morning flowering trait. (a) Graphical genotype of recombinant plants near the emf3‐1D allele. White bars represent the background cultivar genotypes and black bars represent the emf3‐1D allele genotypes. DNA markers with physical positions on chromosome 3 are shown in the column, whereas genotypes and flower opening time (FOT) phenotype are shown in the left and right rows, respectively. (b) Annotations surrounding the polymorphism responsible for the early morning flowering trait. Arrows with numbers (e.g., 5300 F1) indicate the primer binding sites used in RT‐PCR, with the sequence of each primer provided in Table S11. The location of the polymorphic site is also indicated. (c) RT‐PCR detection of transcripts for candidate genes around the polymorphic site. Genes include 5898 (a homologue of maize gene BT065989), 5300 (Os03t0145300), 5400 (Os03t0145400). Actin 1 (Os03g0718100) was included as a control. Genomic DNA and cDNA synthesised from RNA extracted from the panicle were used as templates. (d) Identified transcription units near the polymorphic site and the structure of Os03t0145400. Arrows represent the binding sites of primers used for RT‐PCR. Figure S3: Nucleotide and amino acid sequences of Os03g0145400. (a) The nucleotide [file PBI-24-4780-s001.docx]

**Supporting Information for**

Rice *EMF3* alleles adjust flower opening time to enhance seed setting rate under high temperature stress.

Takuma Ishizaki, Yoichi Hashida, Hideyuki Hirabayashi, Kazuhiro Sasaki, Hiroki Tokunaga, Eliza Vie M. Simon-Ada, Masataka Wakayama, Toshiyuki Takai, Hiroki Saito, Atsushi J. Nagano, Hitoshi Sakakibara, Mikiko Kojima, Yumiko Takebayashi, Sung-Ryul Kim, Ryo Matsushima, Michael J. Thomson, Kazuhiko Sugimoto, Ken-ichiro Hibara*, Tsutomu Ishimaru*

* To whom correspondence may be addressed: Ken-ichiro Hibara or Tsutomu Ishimaru

Email: [ishimaru.tsutomu869@naro.go.jp](mailto:ishimaru.tsutomu869@naro.go.jp) (K.H.) or [hibara@kiui.ac.jp](mailto:hibara@kiui.ac.jp) (T.I.)

**This PDF file includes:**

Figures S1 to S20

Tables S1 to S11


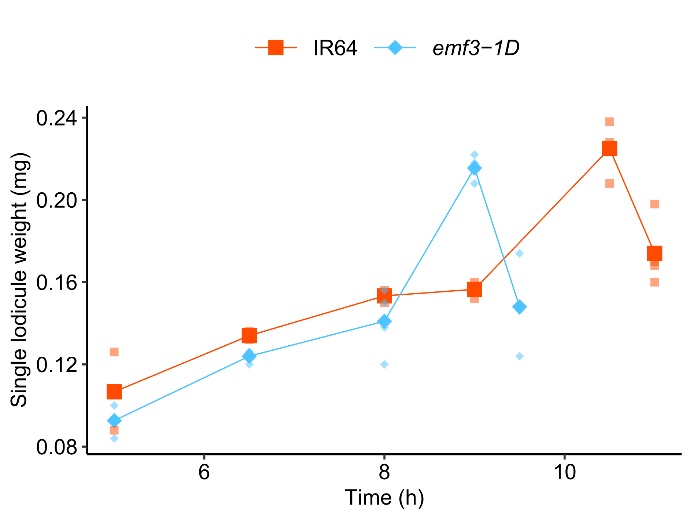


**Figure S1.** Quantitative comparison of fresh lodicule weights between genotypes at different time points. Each large symbol represents the mean value from three biological replicates, whereas each small symbol represents the observed raw value in each replicate. Sampling time points were at 05:00 (30 min before sunrise), 06:30 (1 h after sunrise), 08:00 (2.5 h and 1 h before peak flower opening time (FOT) in IR64 and the *emf3-1D* allele, respectively), 09:00 (1.5 h before peak FOT in IR64 and at peak FOT in the *emf3-1D* allele), 09:30 (30 min after peak of FOT in the *emf3-1D* allele), 10:30 (peak FOT in IR64), 11:00 (30 min after peak FOT in IR64).


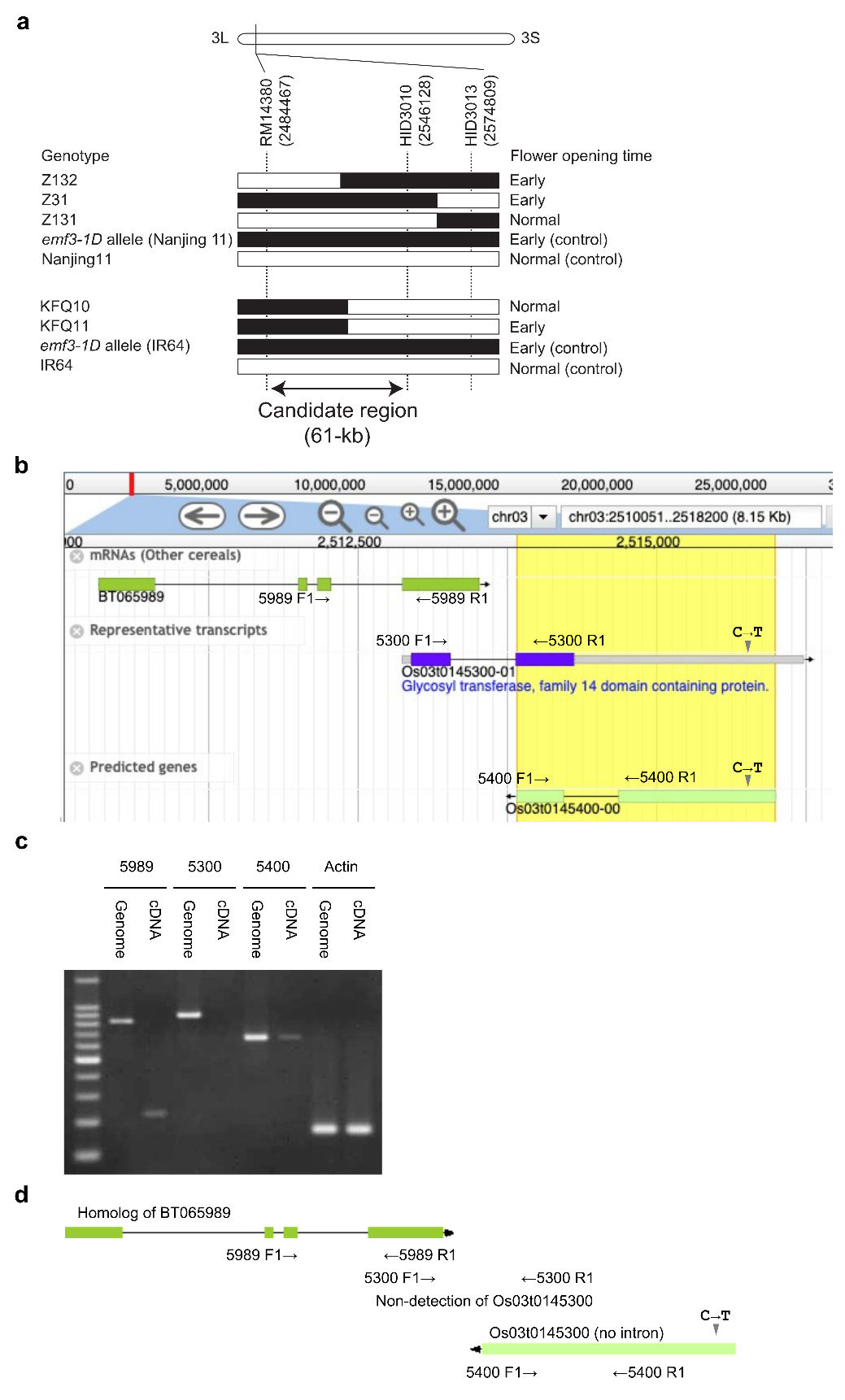
**Figure S2.** Identification of transcription units around the polymorphism conferring the early morning flowering trait. **(a)** Graphical genotype of recombinant plants near the *emf3-1D* allele. White bars represent the background cultivar genotypes, and black bars represent the *emf3-1D* allele genotypes. DNA markers with physical positions on chromosome 3 are shown in the column, whereas genotypes and flower opening time (FOT) phenotype are shown in the left and right row, respectively. **(b)** Annotations surrounding the polymorphism responsible for the early morning flowering trait. Arrows with numbers (e.g. 5300 F1) indicate the primer binding sites used in RT-PCR, with the sequence of each primer provided in Table S11. The location of the polymorphic site is also indicated. **(c)** RT-PCR detection of transcripts for candidate genes around the polymorphic site. Genes include 5898 (a homolog of maize gene *BT065989*), 5300 (*Os03t0145300*), 5400 (*Os03t0145400*). *Actin 1* (*Os03g0718100*) was included as a control. Genomic DNA and cDNA synthesised from RNA extracted from the panicle were used as templates. **(d)** Identified transcription units near the polymorphic site and the structure of *Os03t0145400*. Arrows represent the binding sites of primers used for RT-PCR.

**Figure S3.** Nucleotide and amino acid sequences of *Os03g0145400.* **(a)** The nucleotide sequence of *Os03g0145400* cDNA is shown, with the CDS region highlighted in red. **(b)** The predicted amino acid sequence of *Os03g0145400*. Full-length cDNA was obtained by RACE using gene-specific primers 3'RACE F and 5'RACE R (Table S11).

**Figure S4.** Nucleotide sequence comparison of the *EMF3* coding region. IRGC100947 is the accession of *O. officinalis* (the donor of the early morning flowering trait). Only *emf3-1D* shows the single C-to-T nucleotide substitution at the 181^st^ position of the *EMF3* gene (highlighted in red), which is responsible for the early morning flowering trait. Note that genome sequence of two B lines (maintainer line: IR58025B & IR68897B) is the same as that of A lines (male sterile lines; IR58025A & IR68897A) except for mitochondrial genome in the three-line hybrid system. The coding region of *EMF3* was amplified using gene-specific primers 5400_F0_i, 5400_1111R_i, 5400_923F_i, and 5400_11R_i (Table S11).

**Figure S5.** Amino acid sequence comparison of the *EMF3* gene. IRGC100947 is the accession of *O. officinalis* (the donor of the early morning flowering trait). Only *emf3-1D* shows the single L-to-F amino acid substitution at the 61^st^ position in *EMF3* (highlighted in red), which is responsible for the early morning flowering trait.

**
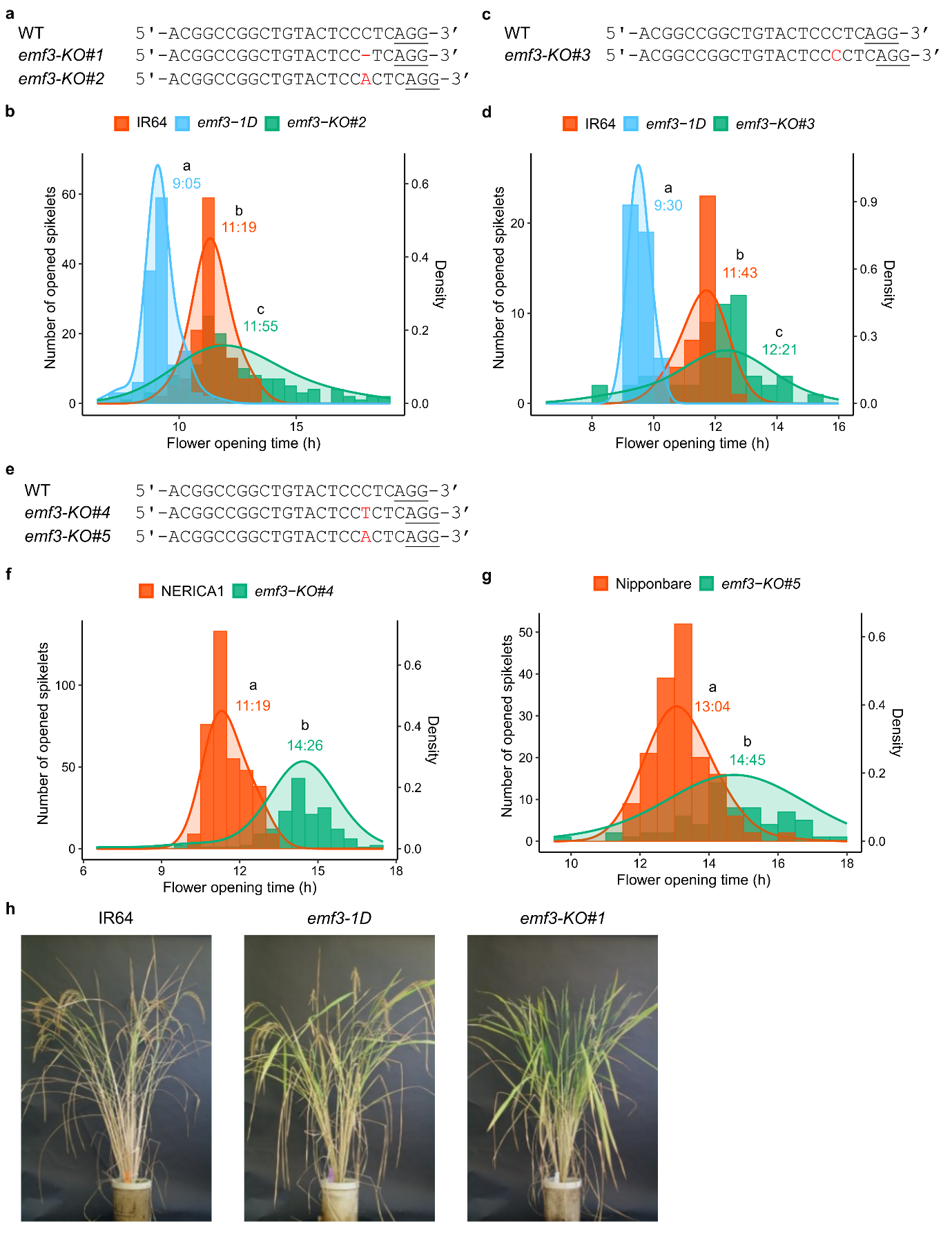
Figure S6.**  FOT and plant appearance in *EMF3* knockout (KO) mutants. **(a)** DNA sequences of mutated *EMF3* in *emf3-KO#1* and *emf3-KO#2* mutants generated by CRISPR/Cas9. **(b)** Observation of FOT for *emf3-KO#2* mutants (the *emf3-1D* allele in an IR64 background), IR64, and *emf3-1D*. FOTs of *emf3-KO#1* are shown in Figure. 1e. **(c)** DNA sequences of mutated *EMF3* in *emf3-KO#3* mutants, generated by CRISPR/Cas9. **(d)** Observation of FOT for the *emf3-KO#3* mutant (IR64 background), IR64, and *emf3-1D*. **(e)** DNA sequences of mutated *EMF3* in *emf3-KO#4* and *emf3-KO#5* mutants, generated by CRISPR/Cas9. **(f)** Observation of FOT of the *emf3-KO#4* mutant (NERICA1 background) and NERICA1. **(g)** Observation of FOT of the *emf3-KO#5* mutant (Nipponbare background) and Nipponbare. In each panel, observation of FOT was conducted on the same days among genotypes. **(h)** Appearances of IR64 (left), *emf3-1D* (center), and *emf3-KO#1* (right) plant at maturity.

In **(a)**, **(c)**, and **(e)**, the underlined sequences indicate the PAM regions. Red letters indicate inserted nucleotides and red dashes indicate deleted nucleotides. In **(b)**, **(d)**, **(f)**, and **(g)**, the histograms represent the raw data of number of opened spikelets at each time point on the basis of observations. The shaded areas indicate the estimated probability density. The times indicated in each panel represent the peak FOTs, estimated from the probability density. Different letters indicate statistically significant differences in peak FOT between the genotypes, on the basis of Bonferroni-adjusted 95% confidence intervals calculated using the bootstrap method.


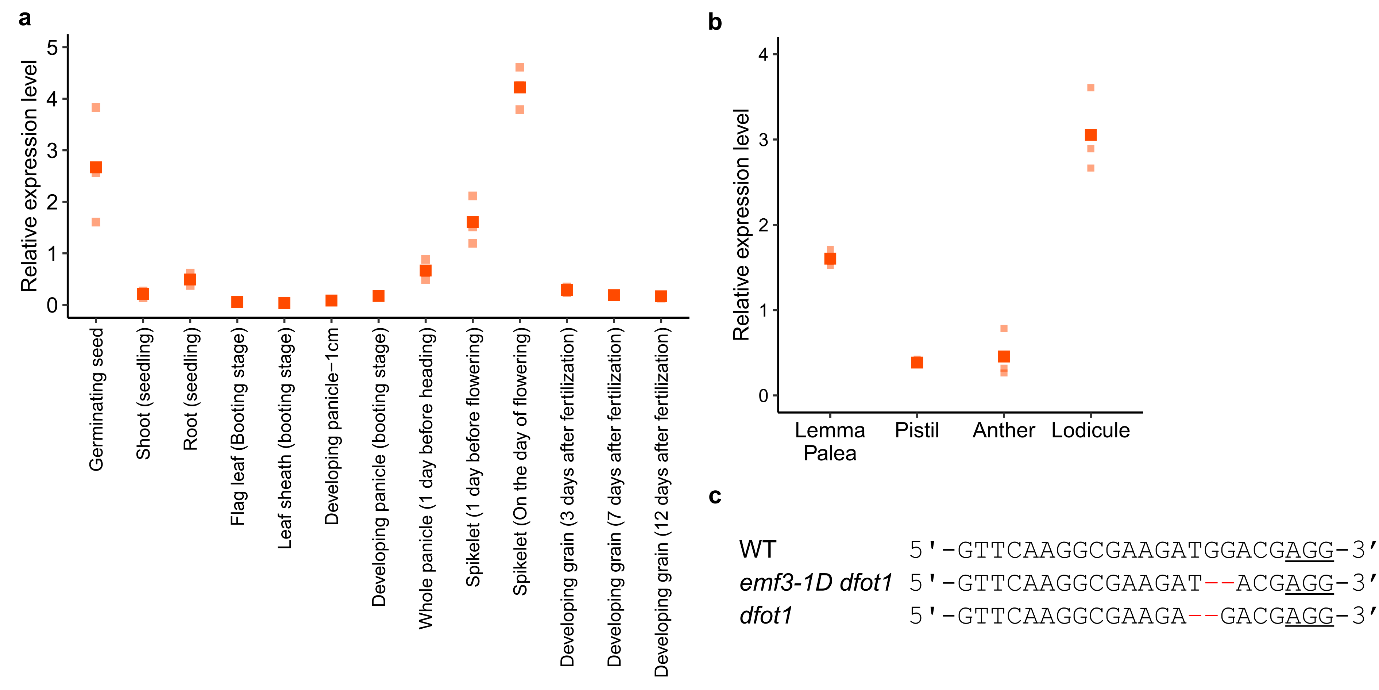


**Figure S7.** Expression analysis of *DFOT1* and DNA sequences of mutated *DFOT1*. **(a)** Quantification of *DFOT1* expression levels in various organs of IR64 throughout the growth stages. **(b)** Quantification of *DFOT1* expression in spikelet organs on the day of flowering. In **(a), (b),** each large symbol represents the mean value from three biological replicates, whereas each small symbol represents the observed raw value in each replicate. **(c)** DNA sequences of mutated *EMF3* generated by CRISPR/Cas9 in the *dfot1* and *emf3-1D doft1* mutants. The underlined sequences indicate PAM regions, and the red dashes mark deleted nucleotides.

**
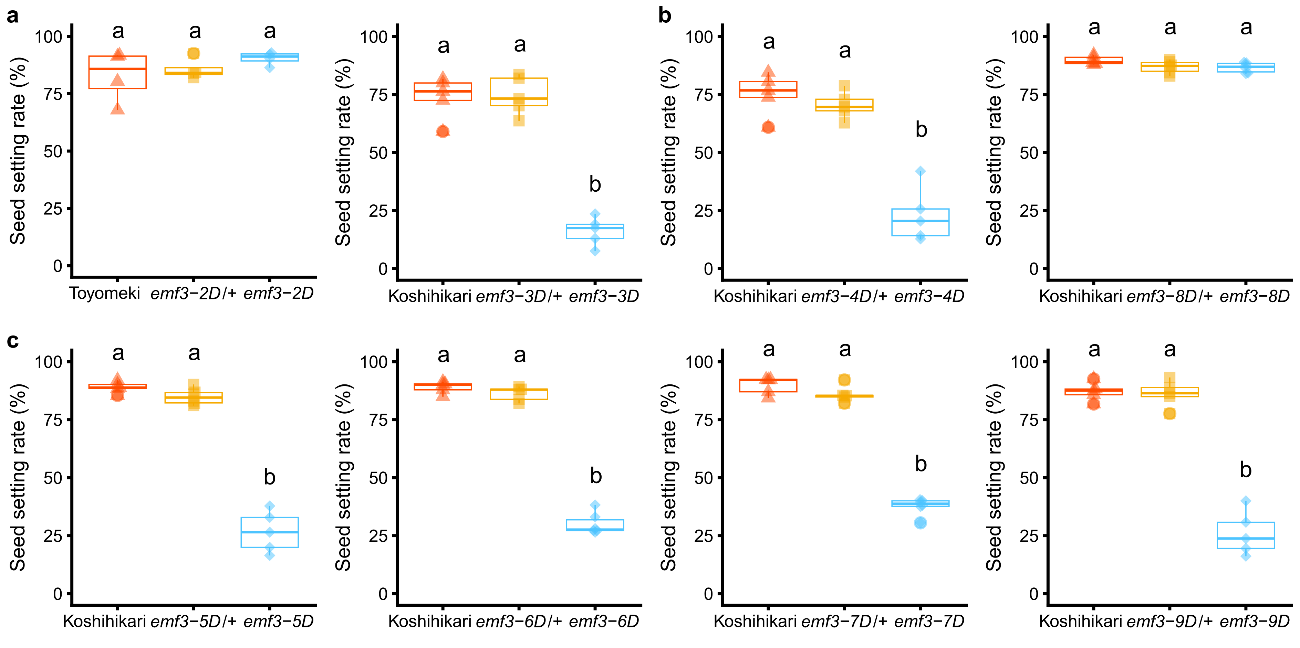
**

**Figure S8.** Seed setting rate in TILLING mutant lines. **(a)** Seed setting rate of mutants which belong to group (i) exhibiting shift to earlier FOT with synchrony. **(b)** Seed setting rate of mutants which belong to group (ii) exhibiting shift to later FOT with synchrony. **(c)** Seed setting rate of mutants which belong to group (iii) exhibiting low FOT synchrony. Different letters indicate significance at the 1% level according to Tukey's method. Each boxplot consists of 4–6 biological replicates, whereas each small symbol represents the observed raw value in each replicate.

**
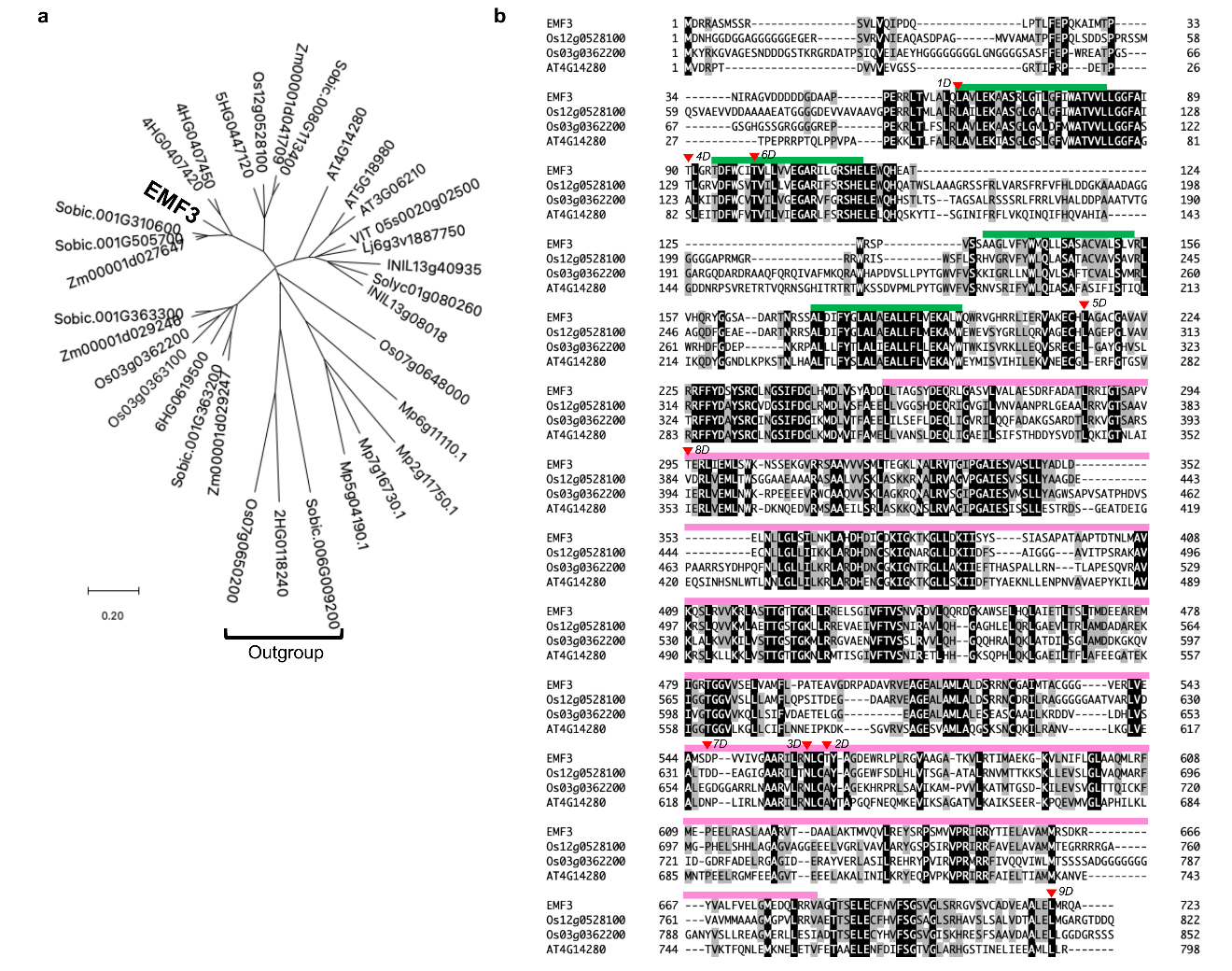
**

**Figure S9.** Phylogenetic tree and sequence alignment analysis of EMF3. **(a)** Phylogenetic tree of the *EMF3* gene constructed using MEGA11 (Tamura et al. 2021). A neighbour-joining tree was inferred from the amino acid sequences of *EMF3* homologs obtained from NCBI (Table S6). Bootstrap values were calculated on the basis of 1,000 replicates. The scale bar indicates genetic distance on the basis of branch length. **(b)** Amino acid sequence alignment of EMF3 and its homologs from rice and Arabidopsis. Fully conserved residues and those matching the majority consensus are highlighted in black and grey boxes, respectively. Predicted transmembrane domains and ARM repeat domain are indicated above the alignment with green and pink lines, respectively. Red triangles denote the sites of mutation identified in the nine *emf3* alleles.

**
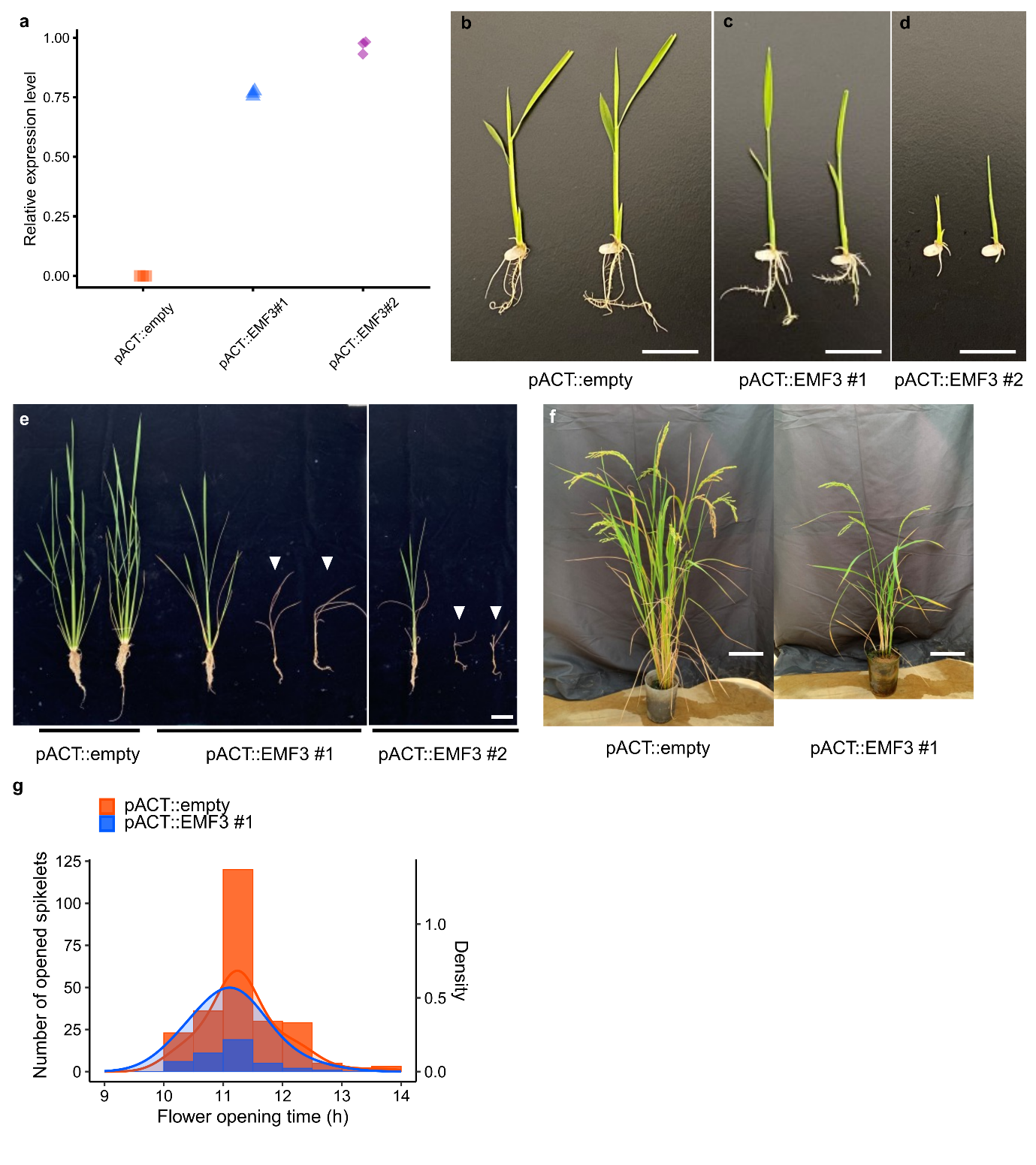
Figure S10.** Effects of *EMF3* overexpression on plant development in transgenic lines. **(a)** Relative expression levels of *EMF3* in pACT::empty (negative control) and pACT::EMF3 lines (#1 and #2). Expression levels were determined by real-time RT-PCR, with 18S rRNA used for normalization. Data represent means ± SD. (**b**-**d**) Seedling phenotypes at 9 days after germination in pACT::empty (**b**), pACT::EMF3 #1 (**c**) and pACT::EMF3 #2 (**d**). pACT::EMF3 #2 (**d**) showed slow growth. Scale bars: 1 cm. **(e)** Morphology of plants at 51 days after germination. Arrowheads indicate individuals that have died. Although the majority of overexpression lines exhibited lethality, a small number of individuals survived until maturity. Scale bars: 5 cm. **(f)** Appearance of rice plants at 2–3 weeks after heading. Scale bars: 12 cm. **(g)** FOT observed in pACT::empty and pACT::EMF3 #1 lines that survived until maturity.


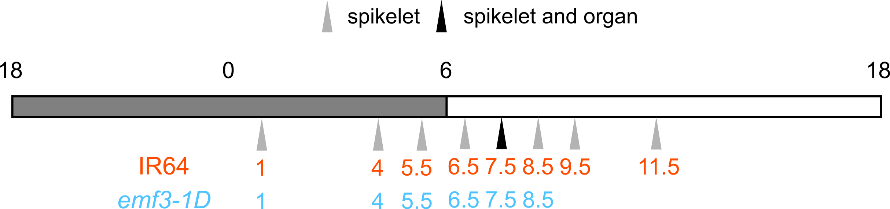


**Figure S11.** Sampling scheme for transcriptome analysis of spikelets of IR64 and *emf3-1D* on the day of flowering*.* The sampling times for each genotype and the types of samples collected are illustrated. Grey and white bars indicate dark and light conditions, respectively. Flower opening begins at 11:30 for IR64 and 8:00 for *emf3-1D*. Time during 18:00-0:00 is the day before the day of flowering.


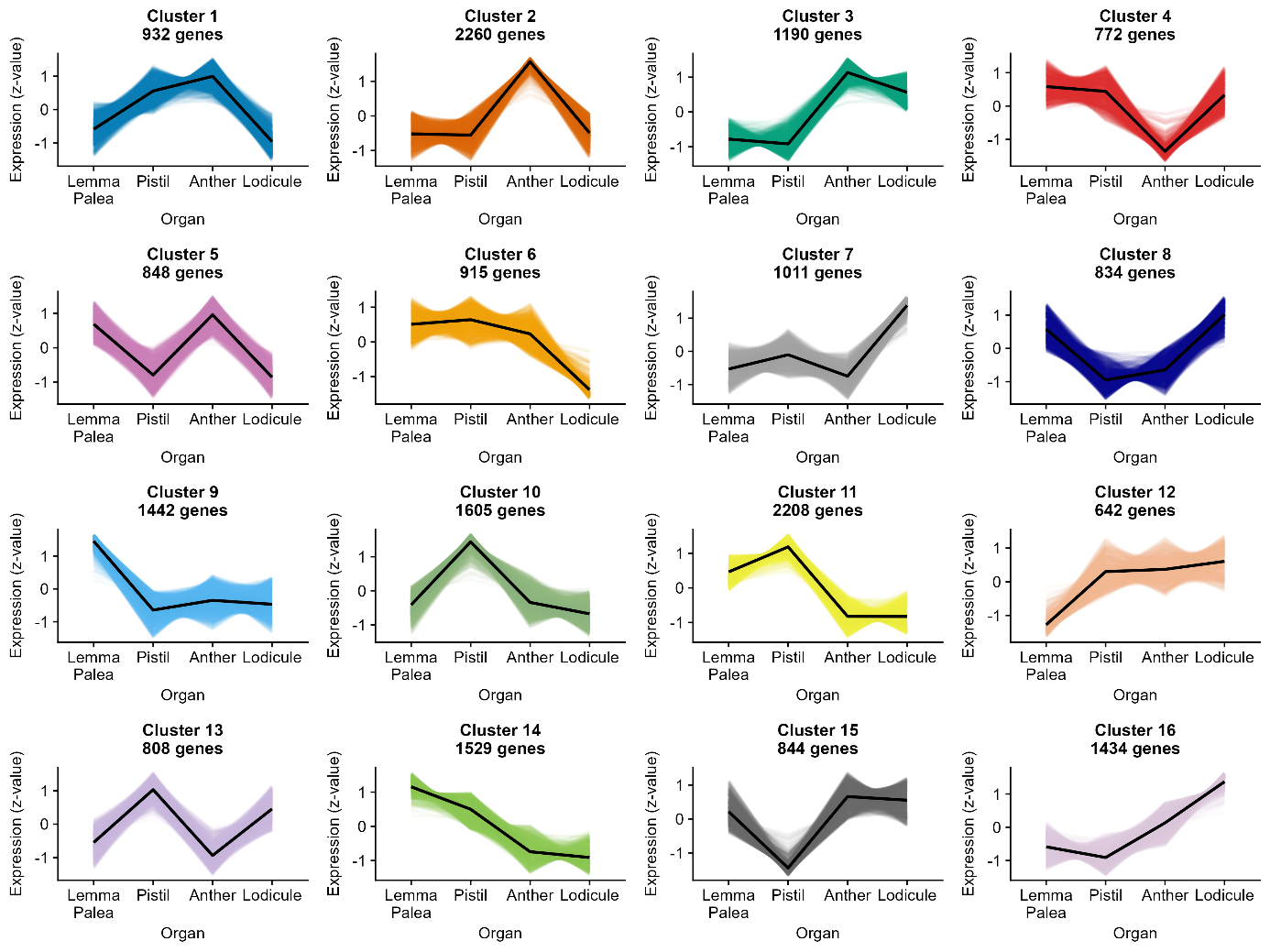


**Figure S12.** K-means clustering of genes expressed in spikelet organs, including the lemma and palea, pistil, anther, and lodicule, of IR64 at 07:30 (light conditions). The genes were grouped into 16 clusters. The normalised expression (z-value) of each gene in each cluster is shown as coloured lines, whereas the mean normalised expression level of the genes in each cluster is represented by the black line.


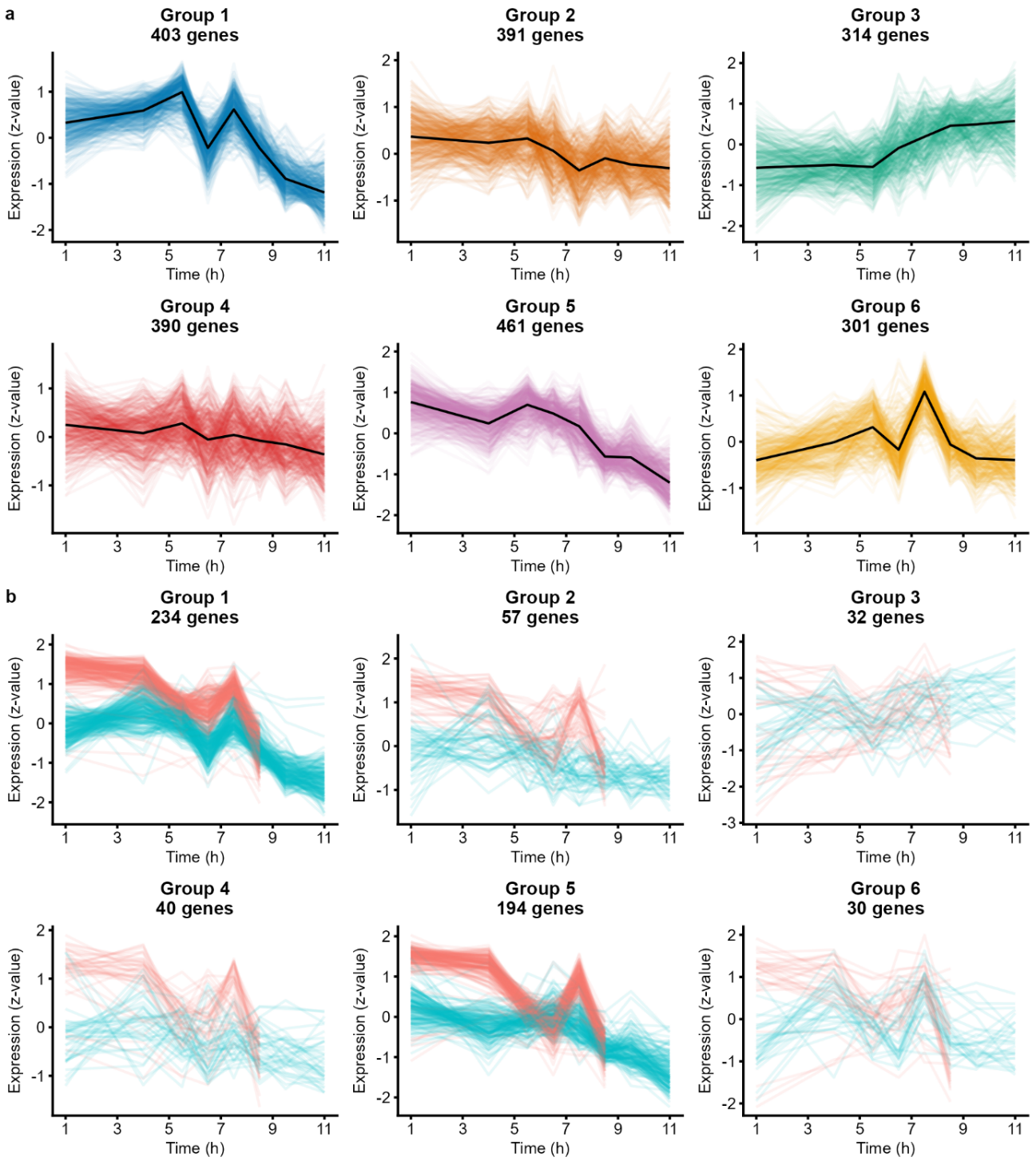


**Figure S13.** K-means clustering of genes in Cluster 2. **(a)** K-means clustering of 2,260 genes in Cluster 2 (from Figure. S12) on the basis of expression patterns in spikelets of IR64 from 01:00 to 11:30. The genes were classified into six groups. The normalised expression (z-value) of each gene in each group is shown by the coloured lines, whereas the mean normalised expression level of the genes in each group is shown by the black line. **(b)** Classification of differentially expressed genes (DEGs) in spikelets between IR64 and the *emf3-1D* allele into six groups, on the basis of the clustering in **(a)**. The normalised expression of each gene is represented by the red and blue lines for IR64 and the *emf3-1D* allele, respectively.


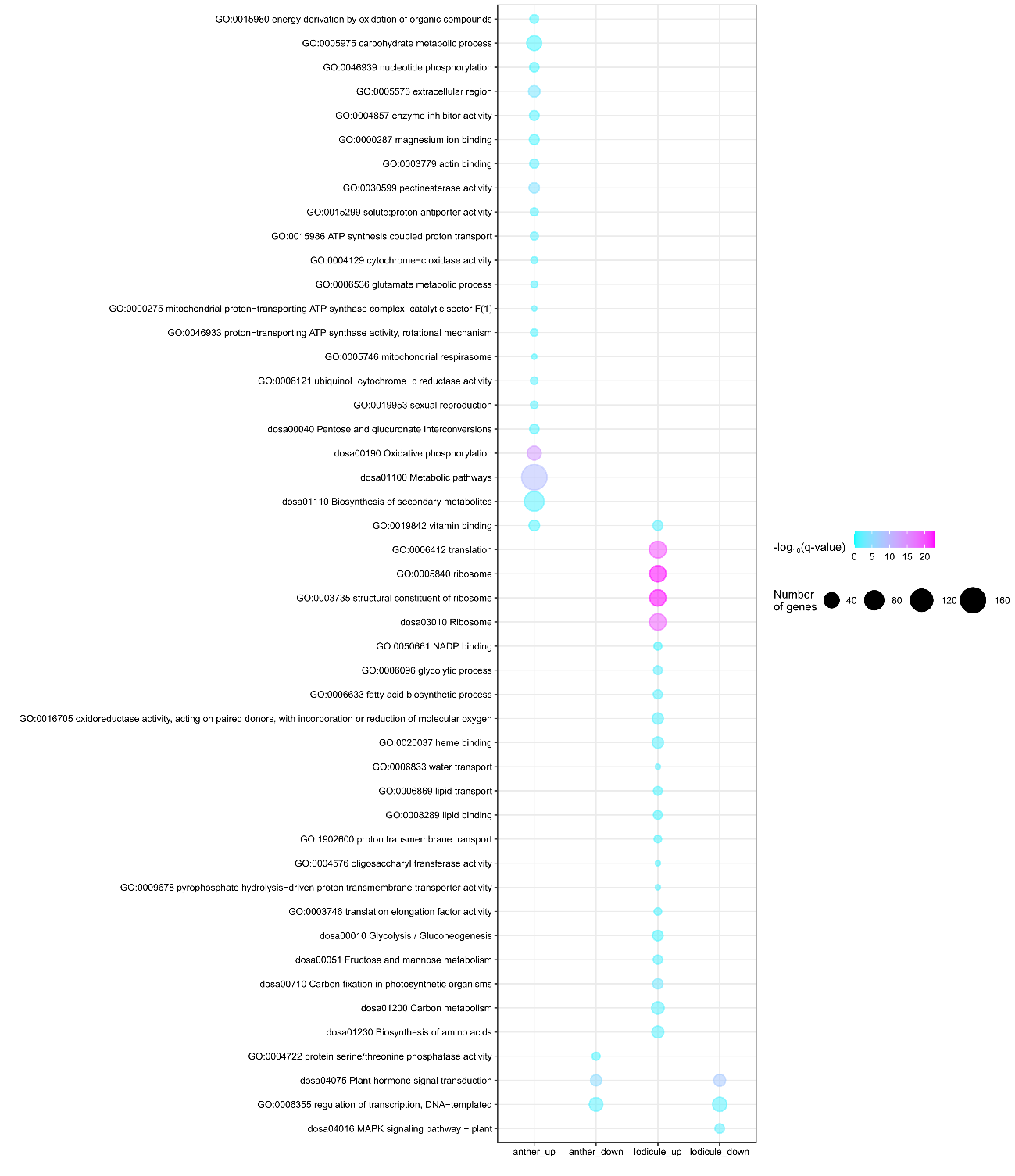


**Figure S14.** GO and KEGG pathways significantly enriched in DEGs between genotypes at 07:30 in the anther and lodicule. The size of the plots represents the number of DEGs, and the colour represents the q-value.

**
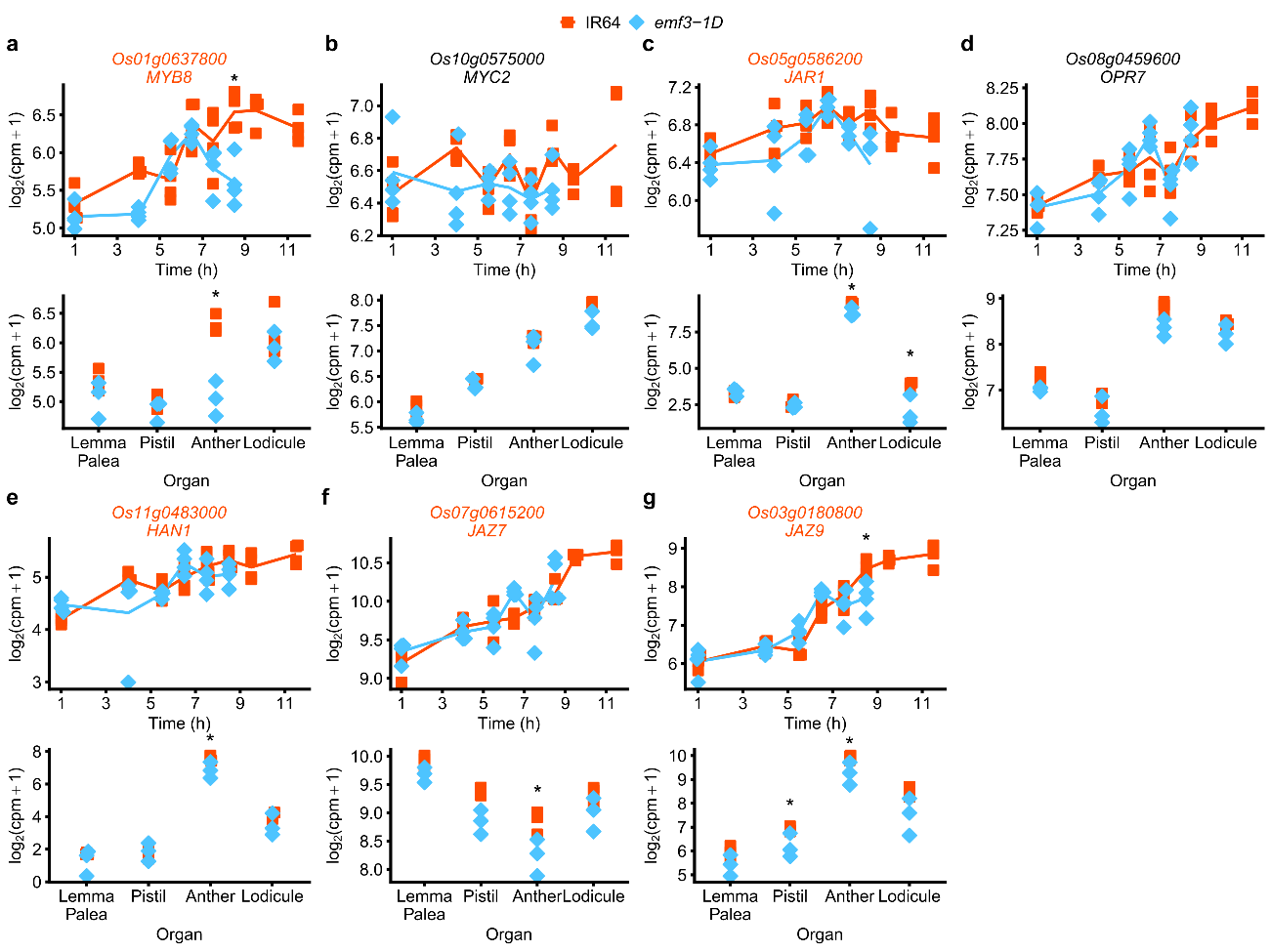
**

**Figure S15.** Expression of JA-related genes in spikelets and spikelet organs of IR64 and *emf3-1D.* Asterisks indicate significant differences in expression (fold change > 1.5, FDR = 0.05) between the genotypes. n = 3–4 (biological replicates). Genes with significant expression differences between the wild-type and *emf3-1D* are indicated in red letter.

**
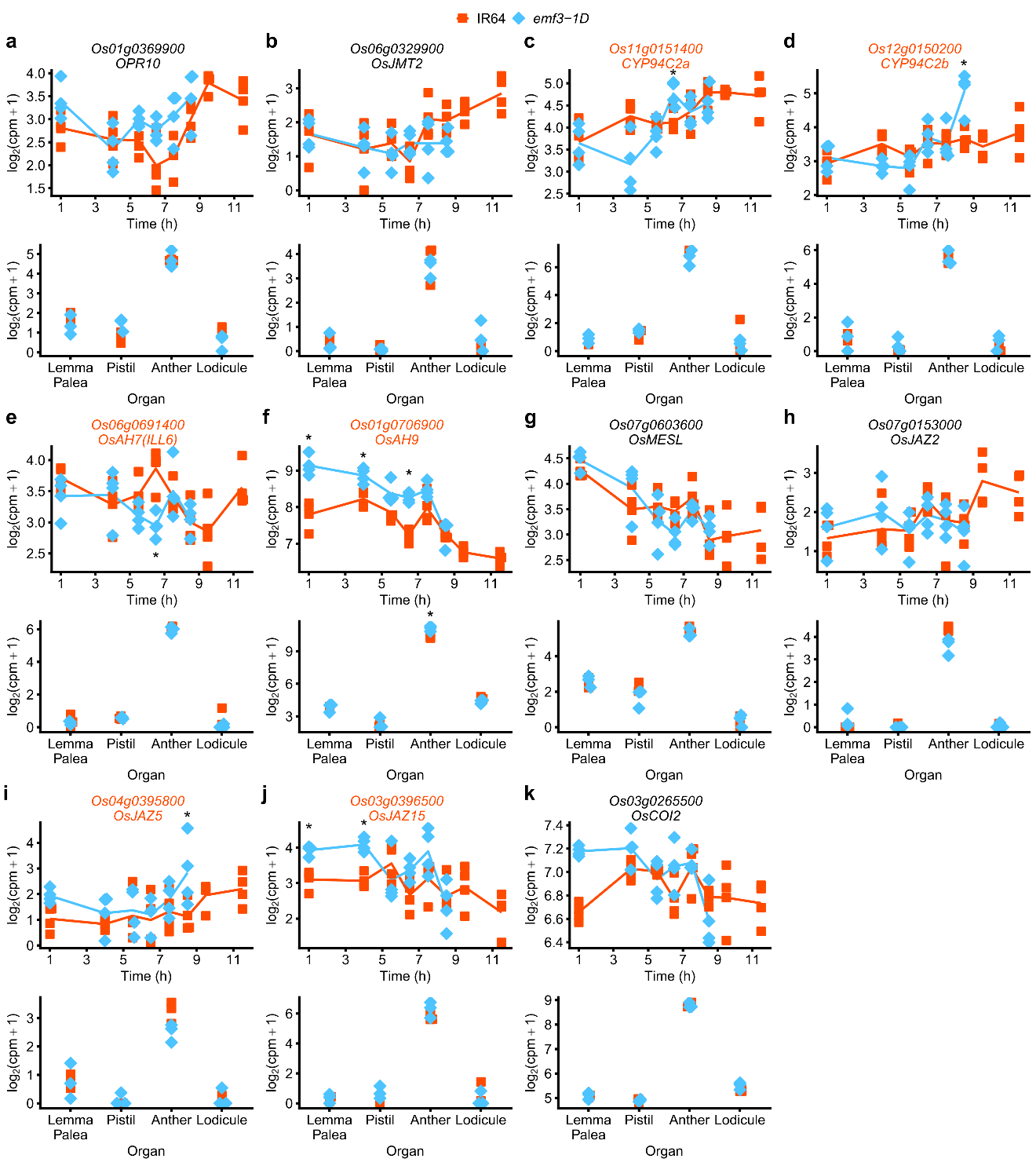
**

**Figure S16.** Expression of JA-related genes belonging to Cluster 2 in spikelets and spikelet organs of genotypes. The expression of *JAR1*, a member of Cluster 2, is not shown in this figure but is provided in Figure. S15c. Asterisks denote significant differences in expression (fold change > 1.5, FDR = 0.05) between the genotypes. n = 3–4 (biological replicates). Genes with significant expression differences between the wild-type and *emf3-1D* are indicated in red letter.

**
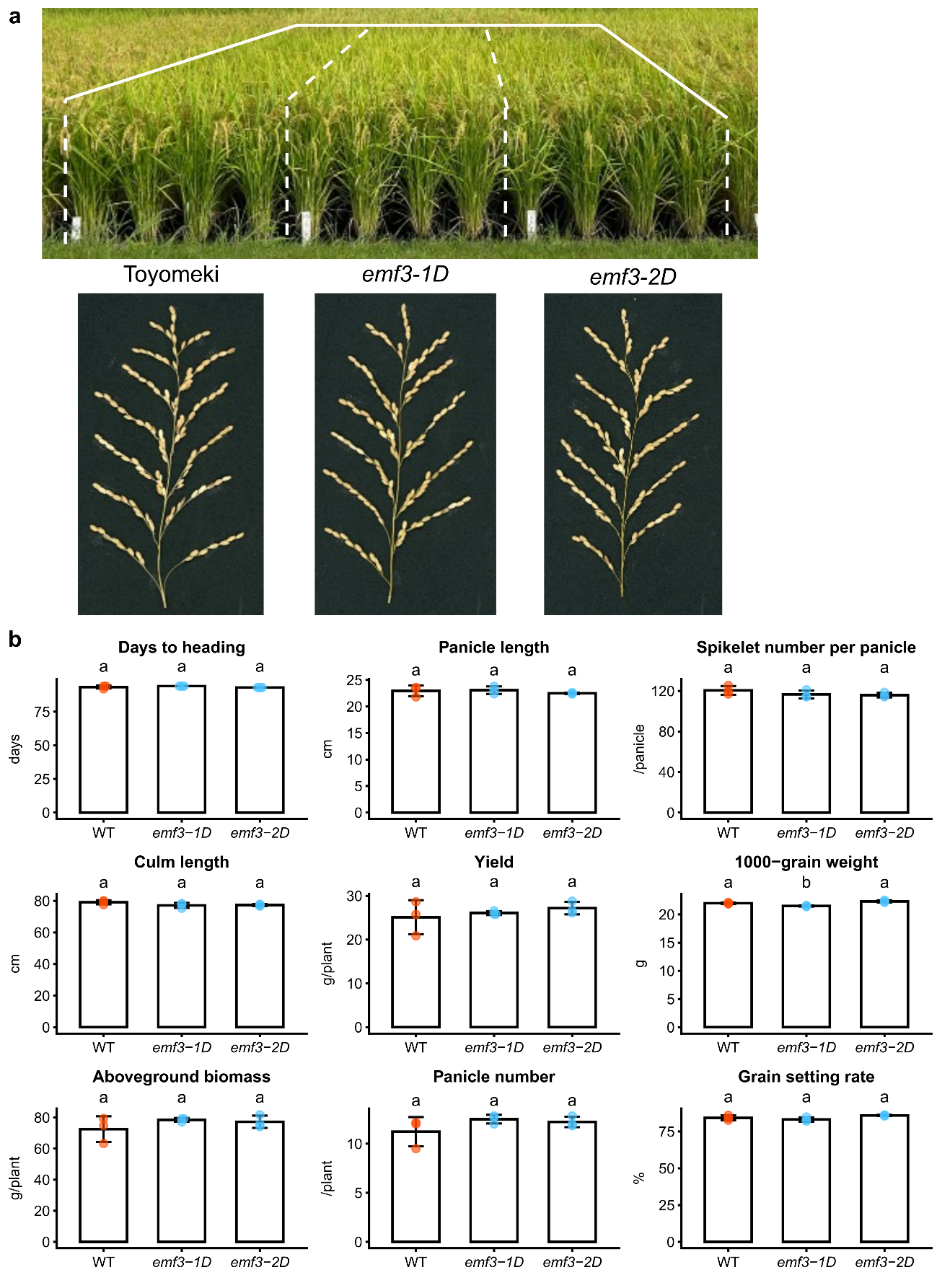
**

**Figure S17.** Evaluation of yield, yield components, and agronomic traits in *emf3-1D* and *emf3-2D* of Toyomeki background in the normal temperature field condition. **(a)** view of demonstration plot and panicle phenotype at maturity. **(b)** yield, yield components, and agronomic traits, n = 3 plots (six plants were harvested per plot). Error bars indicate SD. Different letters indicate significant differences at p < 0.05 according to Tukey–Kramer’s method.


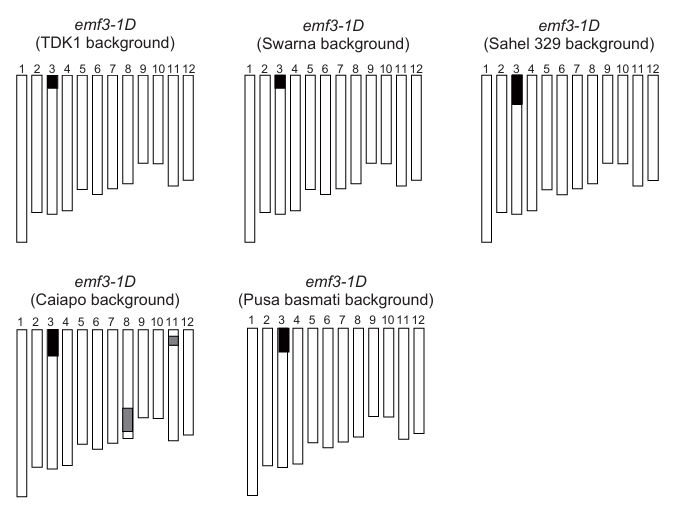


**Figure S18.** Graphical genotype of near-isogenic lines (NILs) with the *emf3-1D* allele against the genetic background of widely grown tropical rice cultivars. White bars represent cultivar background segments, whereas black bars represent segments containing the *emf3-1D* allele on chromosome 3. Grey bars in the *emf3-1D* allele with the Caiapo background represent segments from the IR64 donor parent with the *emf3-1D* allele.


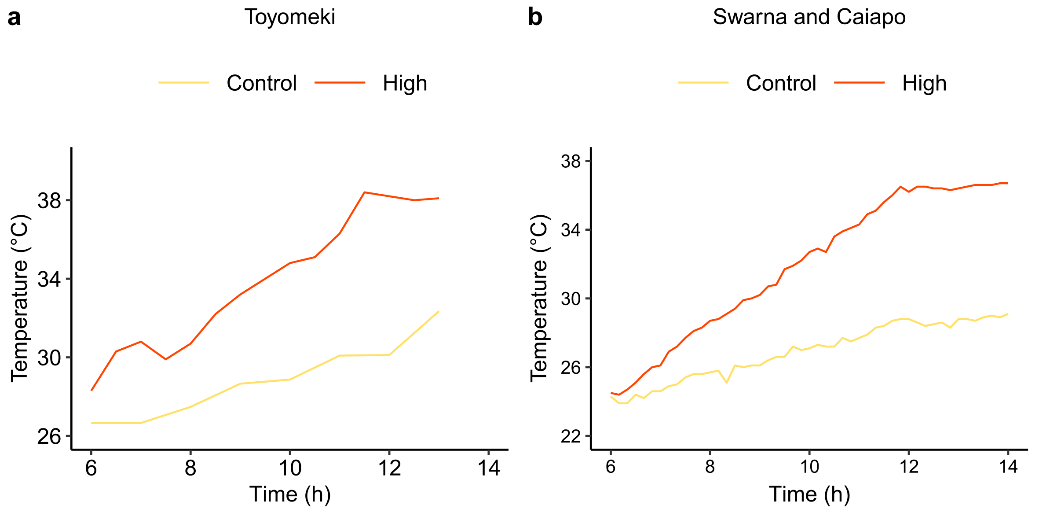
**Figure S19.** Actual temperature at the heat escape tests. **(a)** Test for *emf3-1D* and *emf3-2D* alleles in Toyomeki genetic background. (**b)** Test for *emf3-1D* allele in Swarna or Caiapo genetic background.


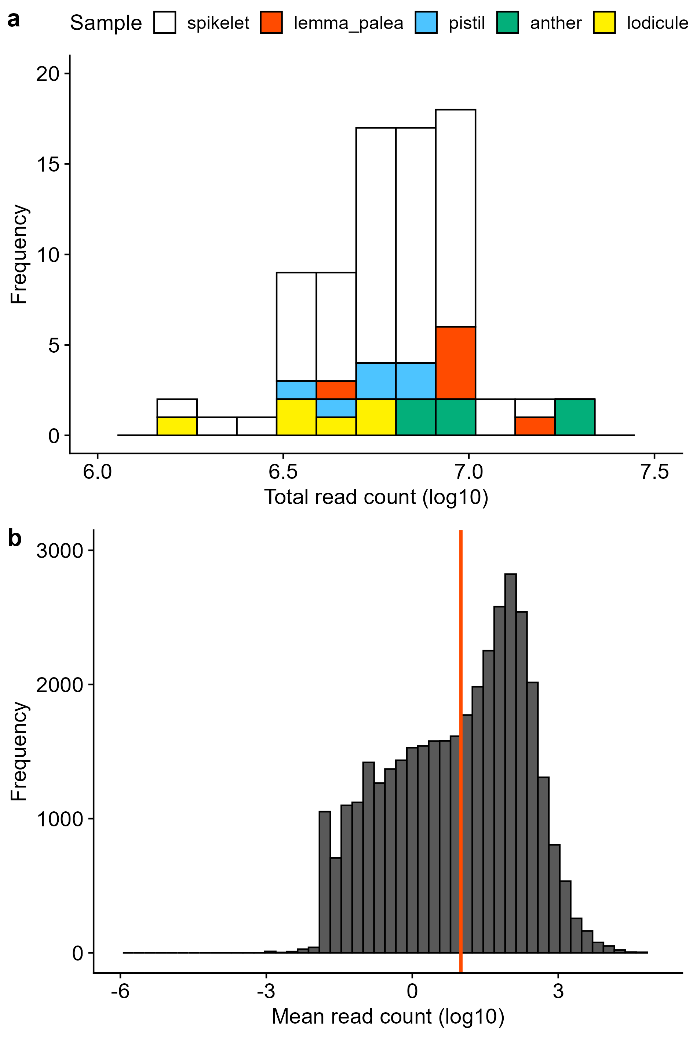


**Figure S20.** RNA-Seq data preprocessing. **(a)** Histogram of the total read counts for each sample, coloured by sample type. **(b)** Histogram of the mean read count for each gene. The red line represents mean read count = 10, the threshold for genes used in the analyses, with those above it considered expressed. After filtering, 19,275 genes were used for the analyses.
