# Supplementary material for: Rice EMF3 Alleles Adjust Flower Opening Time to Enhance the Seed Setting Rate Under High Temperature Stress
Source: Plant Biotechnol J. 2026 Apr 9;24(8):4780–98. doi: 10.1111/pbi.70653 (PMC13387886; doi:10.1111/pbi.70653)
Supplement: Supplementary file 2 — Table S1: Peak FOT estimated from the probability density of FOT. Table S2: Differences by genotypes in peak FOT estimated from the probability density of FOT. Table S3: Mutant lines generated by genome editing. Table S4: Single amino acid mutation lines and FOT by each genotype in BC2F2 generation of the TILLING mutant lines. Table S5: Primer list for selecting single nucleotide substitution lines in TILLING mutant panels. Table S6: List of EMF3 and EMF3 ‐like gene family members in different plants. Table S7: Enriched gene ontology and KEGG pathway in each clusters. Table S8: Clustering of jasmonate‐related genes. Table S9: Enriched gene ontology and KEGG pathway in DEGs between IR64 and emf3‐1D in each spikelet's organ at 7:30. Table S10: Flower opening time of NILs carrying emf3‐1D allele in the heat escape test. Table S11: Sequences of primers used in this study. [file PBI-24-4780-s002.pdf]

Table S1. Peak FOT estimated from the probability density of FOT.

Figure number	Genetic background	Genotype	Peak of DFOT	Maximum value of the Probability density	Observation date
Fig.1b	Nanjing11	WT	10:25	0.924	20090813
	Nanjing11	*emf3-1D* /+	09:31	0.734	20090813
	Nanjing11	*emf3-1D*	09:03	0.549	20090813
Fig.1f	IR64	WT	11:16	0.447	20190822–20190825
	IR64	*emf3-1D*	09:14	0.737	20190822–20190825
	IR64 *emf3-1D*	*emf3-KO#1*	12:50	0.152	20190822–20190825
Fig.S6c	IR64	WT	11:19	0.451	20190817–20190821
	IR64	*emf3-1D*	9:05	0.651	20190817–20190821
	IR64 *emf3-1D*	*emf3-KO#2*	11:55	0.159	20190817–20190821
Fig.S6e	IR64	WT	11:43	0.505	20230328–20230330
	IR64	*emf3-1D*	9:30	1.065	20230328–20230330
	IR64	*emf3-KO#3*	12:21	0.237	20230328–20230329
Fig.S6g	NERICA1	WT	11:19	0.450	20220815–20220822
	NERICA1	*emf3-KO#4*	14:26	0.285	20220815–20220822
Fig.S6h	Nipponbare	WT	13:04	0.396	20220426–20220510
	Nipponbare	*emf3-KO#5*	14:45	0.195	20220426–20220510
Fig.1j	IR64	WT	11:25	0.500	20240306–20240318
	IR64	*emf3-1D*	09:26	0.624	20240305–20240309
	IR64	*dfot1*	11:09	0.305	20240304–20240313
	IR64	*emf3-1D dfot1*	09:46	0.699	20240303–20240308
Fig.2a	Toyomeki	WT	10:28	0.466	20230818
	Toyomeki	*emf3-2D* /+	10:13	0.879	20230818
	Toyomeki	*emf3-2D*	8:49	0.783	20230818
Fig.2a	Koshihikari	WT	10:42	0.561	20230803
	Koshihikari	*emf3-3D* /+	8:05	0.642	20230803
	Koshihikari	*emf3-3D*	11:40	0.126	20230803
Fig.2b	Koshihikari	WT	10:49	0.826	20230803
	Koshihikari	*emf3-4D* /+	11:17	0.497	20230803
	Koshihikari	*emf3-4D*	13:06	0.279	20230803
Fig.2b	Koshihikari	WT	10:54	0.411	20230805
	Koshihikari	*emf3-8D* /+	11:05	0.576	20230805
	Koshihikari	*emf3-8D*	11:49	0.262	20230805
Fig.2c	Koshihikari	WT	9:22	0.659	20230802
	Koshihikari	*emf3-5D* /+	9:54	0.397	20230802
	Koshihikari	*emf3-5D*	10:16	0.179	20230802
Fig.2c	Koshihikari	WT	11:12	0.748	20230804
	Koshihikari	*emf3-6D* /+	11:50	0.392	20230804
	Koshihikari	*emf3-6D*	11:24	0.156	20230804
Fig.2c	Koshihikari	WT	10:29	0.632	20230804
	Koshihikari	*emf3-7D* /+	11:19	0.652	20230804
	Koshihikari	*emf3-7D*	11:43	0.153	20230804
Fig.2c	Koshihikari	WT	10:40	0.336	20230805
	Koshihikari	*emf3-9D* /+	12:50	0.175	20230805
	Koshihikari	*emf3-9D*	11:04	0.119	20230805
Fig.2d	Koshihikari	WT	12:20	0.589	20240517–20240524
	Koshihikari	*emf3-#T563I/+*	10:54	0.297	20240519–20240524
	Koshihikari	*emf3-#T563I*	10:20	0.420	20240517–20240524
Fig.2d	Koshihikari	WT	12:20	0.589	20240517–20240524
	Koshihikari	*#T563I*	10:20	0.420	20240517–20240524
	Koshihikari	*#C562W/T563I*	14:10	0.188	20240522–20240524
Fig.2d	Koshihikari	WT	11:54	0.494	20250610-20250615
	Koshihikari	*#A62V*	14:07	0.411	20250610-20250615
Fig.2d	Koshihikari	WT	12:03	0.345	20250808-20250815
	Koshihikari	*#V105I*	12:30	0.477	20250804-20250811
	Koshihikari	*#V104I/V105I*	14:19	0.301	20250806-20250811
	Koshihikari	*#V104I/V105I/E106K*	14:07	0.233	20250803-20250810
Fig.5a	CG14	WT	08:00	0.368	20230818
	IR64	WT	08:58	0.750	20230818
	Toyomeki	WT	10:18	0.926	20230818
Fig.5b	CG14	WT	08:00	0.368	20230818
	IR64	*emf3-1D*	07:23	0.343	20230818
	IR64	WT	08:58	0.750	20230818
Fig.5c	IR64	WT	08:58	0.750	20230818
	Toyomelki	*emf3-2D*	08:49	0.783	20230818
	Toyomeki	WT	10:18	0.926	20230818
Fig.5d	IR64	WT	10:15	2.190	20240731
	IR64	*emf3-1D*	08:42	0.389	20240731
	Caiapo	WT	11:44	0.767	20240801
	Caiapo	*emf3-1D*	10:00	0.615	20240801
	Swarna	WT	09:48	0.756	20240724
	Swarna	*emf3-1D*	08:49	0.586	20240724
	Sahel 329	WT	10:17	0.790	20240724
	Sahel 329	*emf3-1D*	09:20	0.689	20240724
	Pusa Basmati	WT	10:09	0.629	20240730
	Pusa Basmati	*emf3-1D*	08:23	0.280	20240730
	TDK1	WT	10:35	0.527	20240731
	TDK1	*emf3-1D*	09:22	0.450	20240731
Fig. 6a	Toyomeki	WT	10:21	0.645	240802
	Toyomeki	*emf3-1D*	09:03	0.379	240802
	Toyomeki	*emf3-2D*	09:24	0.801	240802
Fig. 6a	Toyomeki	WT	10:39	0.370	240802
	Toyomeki	*emf3-1D*	09:17	0.697	240802
	Toyomeki	*emf3-2D*	09:39	0.775	240802

Table S2. Differences by genotypes in peak FOT estimated from the probability density of FOT.

Figure number	Delta in Peak of FOT		Bonferroni-adjusted 95% confidence interval	Statistical significance in peak of FOT
Fig.1b	WT - *emf3-1D/+*	00:54	00:50, 00:59	*
	WT - *emf3-1D*	01:22	01:17, 01:28	*
	emf3-1D/+ - emf3-1D	00:28	00:24, 00:33	*
Fig.1f	WT - *emf3-1D*	02:02	01:50, 02:15	*
	WT - *emf3-KO#1*	-01:34	-02:10, -01:02	*
	emf3-1D - emf3-KO#1	-03:36	-04:10, -03:04	*
Fig.S6c	WT - *emf3-1D*	02:14	02:05, 02:22	*
	WT - *emf3-KO#2*	-00:35	-01:10, -00:14	*
	emf3-1D - emf3-KO#2	-02:49	-03:23, -02:25	*
Fig.S6e	WT - *emf3-1D*	02:13	01:54, 02:23	*
	WT - *emf3-KO#3*	-00:38	-01:07, -00:07	*
	emf3-1D - emf3-KO#3	-02:51	-03:13, -02:21	*
Fig.S6g	WT - *emf3-KO#4*	-03:07	-03:17, -02:59	*
Fig.S6h	WT - *emf3-KO#5*	-01:41	-02:04, -01:13	*
Fig.1j	WT - *emf3-1D*	01:59	01:49, 02:14	*
	WT - *dfot1*	00:16	00:00, 00:36	*
	WT - *emf3-1D dfot1*	01:30	01:19, 01:39	*
	emf3-1D - dfot1	-01:43	-02:02, -01:26	*
	emf3-1D - emf3-1D dfot1	-00:30	-00:45, -00:22	*
	dfot1 - emf3-1D dfot1	01:14	00:56, 01:29	*
Fig.2a	WT - *emf3-2D/+*	00:15	00:05, 00:22	*
	WT - *emf3-2D*	01:40	01:28, 01:46	*
	emf3-2D/+ - emf3-2D	01:24	01:16, 01:26	*
Fig.2a	WT - *emf3-3D/+*	02:37	02:22, 02:51	*
	WT - *emf3-3D*	-00:58	-01:41 , -00:01	*
	emf3-3D/+ - emf3-3D	-03:34	-04:16, -02:39	*
Fig.2b	WT - *emf3-4D/+*	-00:29	-00:33, -00:23	*
	WT - *emf3-4D*	-02:17	-02:34, -02:03	*
	emf3-4D/+ - emf3-4D	-01:48	-02:06, -01:34	*
Fig.2b	WT - *emf3-8D/+*	-00:11	-00:20, 00:04	ns
	WT - *emf3-8D*	-00:55	-01:08, -00:38	*
	emf3-8D/+ - emf3-8D	-00:44	-01:01, -00:29	*
Fig.2c	WT - *emf3-5D/+*	-00:32	-00:43, -00:21	*
	WT - *emf3-5D*	-00:54	-01:24, -00:28	*
	emf3-5D/+ - emf3-5D	-00:22	-00:50, 00:06	ns
Fig.2c	WT - *emf3-6D/+*	-00:37	-00:43, -00:29	*
	WT - *emf3-6D*	-00:12	-00:34, 00:14	ns
	emf3-6D/+ - emf3-6D	00:25	00:02, 00:52	*
Fig.2c	WT - *emf3-7D/+*	-00:50	-01:00, -00:38	*
	WT - *emf3-7D*	-01:14	-01:38, -00:46	*
	emf3-7D/+ - emf3-7D	-00:24	-00:45, 00:01	ns
Fig.2c	WT - *emf3-9D/+*	-02:10	-02:26, -01:32	*
	WT - *emf3-9D*	-00:24	-01:33, 00:33	ns
	emf3-9D/+ - emf3-9D	01:46	00:24, 02:36	*
Fig.2d	WT - *#T563I/+*	01:26	01:08, 01:43	*
	WT - *#T563I*	02:00	01:49, 02:13	*
	#T563I/+ - #T563I	00:34	00:18, 00:53	*
Fig.2d	WT - *#T563I*	02:00	01:49, 02:13	*
	WT - #C562W/*T563I*	-01:50	-02:43, -00:52	*
	#T563I - #C562W/T563I	-03:51	-04:45, -02:51	*
Fig.2d	WT - *#A62V*	-02:13	-02:30, -01:52	*
Fig.2d	WT - *#V105I*	-00:26	-00:41, -00:10	*
	WT - *#V104I/V105I*	-02:16	-02:37, -01:54	*
	WT - *#V104I/V105I/E106K*	-02:03	-02:30, -01:39	*
	#V105I - *#V104I/V105I*	-01:49	-02:07, -01:32	*
	#V105I - #V104I/V105I/E106K	-01:37	-01:59, -01:17	*
	#V104I/V105I - *#V104I/V105I/E106K*	00:12	-00:14, 00:38	ns
Fig.5a	CG14 - IR64	-00:58	-01:17, -00:41	*
	CG14 - Toyomeki	-02:18	-02:35, -02:03	*
	IR64 - Toyomeki	-01:20	-01:27, -01:12	*
Fig.5b	CG14 - *emf3-1D* (IR64)	00:36	00:13, 00:58	*
	CG14 - IR64	-00:58	-01:17, -00:41	*
	emf3-1D (IR64) - IR64	-01:34	-01:51, -01:16	*
Fig.5c	IR64 - *emf3-2D* (Toyomeki)	00:09	-00:02, 00:16	ns
	IR64 - Toyomeki	-01:20	-01:27, -01:12	*
	emf3-2D (IR64) - Toyomeki	-01:29	-01:31, -01:20	*
Fig.5d	IR64 - *emf3-1D*	01:33	01:19, 01:45	*
	Caiapo - *emf3-1D*	01:45	01:35, 01:55	*
	Swarna - *emf3-1D*	00:59	00:55, 01:15	*
	Sahel 329 - *emf3-1D*	00:57	00:52, 01:01	*
	Pusa Basmati - *emf3-1D*	01:46	01:22, 02:03	*
	TDK1 - *emf3-1D*	01:14	01:03, 01:29	*
Fig.6a	Toyomeki - *emf3-1D*	01:18	01:01, 01:36	*
	Toyomeki - *emf3-2D*	00:57	00:47, 01:08	*
	emf3-1D - emf3-2D	-00:21	-00:40, -00:05	*
Fig.6a	Toyomeki - *emf3-1D*	01:21	01:11, 01:32	*
	Toyomeki - *emf3-2D*	01:00	00:49, 01:10	*
	emf3-1D - emf3-2D	-00:21	-00:29, -00:16	*

Table S3. Mutant lines generated by genome editing.

Line	Background	Target gene	Mutated DNA sequence (5'–3')	FOT phenotype	
				Synchrony	Peak time
emf3-KO#1	IR64/*emf3-1D*	*EMF3*	ACGGCCGGCTGTACTCC–TCAGG	Low	Late
emf3-KO#2	IR64/*emf3-1D*	*EMF3*	ACGGCCGGCTGTACTCCACTCAGG	Low	Late
emf3-KO#3	IR64	*EMF3*	ACGGCCGGCTGTACTCCCCTCAGG	Low	Late
emf3-KO#4	NERICA1	*EMF3*	ACGGCCGGCTGTACTCCTCTCAGG	Moderate	Late
emf3-KO#5	Nipponbare	*EMF3*	ACGGCCGGCTGTACTCCACTCAGG	Low	Late
emf3-#T563I	Koshihikari	*EMF3*	CCTCCGCAACCTGTGTATTTACGCC	Moderate	Early
dfot1	IR64	*DFOT1*	GTTCAAGGCGAAGAT—ACGAGG	Moderate	Early
emf3-1D dfot1	IR64/*emf3-1D*	*DFOT1*	GTTCAAGGCGAAGA—GACGAGG	High	Early
emf3-#C562W/T563I	Koshihikari	*EMF3*	CCTCCGCAACCTGTGGATTTACGCC	Low	Late
emf3-#A62V	Koshihikari	*EMF3*	CTGGCCCTGCAGCTTGTTGTCCTC	Moderate	Late
emf3-#V105I	Koshihikari	*EMF3*	TCACCGTGCTGCTAGTAATAGAGGGC	Moderate	Late
emf3-#V104I/V105I	Koshihikari	*EMF3*	TCACCGTGCTGCTGATAATAGAGGGC	Moderate	Late
emf3-#V104I/V105I/E106K	Koshihikari	*EMF3*	TCACCGTGCTGCTAATAATAAAGGGC	Low	Late

Red letters or dahse denote deleted, inserted, or substituted nucleotides.

Degree of synchrony was defined based on the peak of probability density of flower opening time as follows:

Low: < 0.25

Moderate: 0.25 – 0.50

High: > 0.50

Peak time of flower opening in mutants was classified by comparison with their background cultivars.

Table S4. Single amino acid mutation lines and FOT by each genotype in BC_2F_2 generation of the TILLING mutant lines.

Line name	Background	Nucleoide substitution			Amino acid substitution			FOT phenotype			
								Peak time		Synchrony	
		Position	Wild type	Mutantion	Position	Wild type	Mutantion	Mutant homo	Hetero	Mutant homo	Hetero
emf3-1D	**Koshihikari**	**181**	**C**	**T**	**61**	**LEU**	**PHE**	**Early**	**Inter.**	**High**	**High**
emf3-4D	**Koshihikari**	**268**	**A**	**T**	**90**	**THR**	**SER**	**Late**	**Inter.**	**Moderate**	**Moderate**
emf3-6D	**Koshihikari**	**299**	**C**	**T**	**100**	**THR**	**ILE**	**Inter.**	**Inter.**	**Low**	**Moderate**
emf3-5D	**Koshihikari**	**644**	**T**	**G**	**215**	**LEU**	**ARG**	**Late**	**Inter.**	**Low**	**Moderate**
emf3-8D	**Koshihikari**	**887**	**A**	**G**	**296**	**GLU**	**GLY**	**Late**	**Inter.**	**Moderate**	**High**
emf3-7D	**Koshihikari**	**1640**	**A**	**T**	**547**	**ASP**	**VAL**	**Late**	**Inter.**	**Low**	**High**
emf3-3D	**Koshihikari**	**1679**	**A**	**T**	**560**	**ASN**	**ILE**	**Late**	**Early**	**Low**	**High**
emf3-9D	**Koshihikari**	**2156**	**T**	**A**	**719**	**LEU**	*****	**Inter.**	**Late**	**Low**	**Low**
emf3-2D	**Toyomeki**	**1688**	**C**	**T**	**563**	**THR**	**ILE**	**Early**	**Inter.**	**High**	**High**
0257F	Koshihikari	241	G	A	81	VAL	MET	Not different from wild type			
2161W	Koshihikari	505	A	T	169	THR	SER	Not different from wild type			
0274N	Koshihikari	506	C	T	169	THR	ILE	Not different from wild type			
0546U	Koshihikari	769	A	T	257	THR	SER	Not different from wild type			
0914N	Koshihikari	1051	C	T	351	LEU	PHE	Not different from wild type			
0054N	Koshihikari	1085	T	A	362	LEU	HIS	Not different from wild type			
0106W	Koshihikari	1469	T	A	490	VAL	GLU	Not different from wild type			
1043N	Koshihikari	1498	G	A	500	VAL	ILE	Not different from wild type			
1514N	Koshihikari	1559	C	T	520	ALA	VAL	Not different from wild type			
0043U	Koshihikari	1666	A	T	556	ARG	TRP	Not different from wild type			
1382W	Koshihikari	1784	T	A	595	LEU	HIS	Not different from wild type			
0292F	Koshihikari	1892	C	T	631	THR	MET	Not different from wild type			
0709U	Koshihikari	1901	A	G	634	GLN	ARG	Not different from wild type			
0116A	Koshihikari	1919	G	A	640	SER	ASN	Not different from wild type			
1435N	Koshihikari	1936	G	A	646	VAL	MET	Not different from wild type			
1902W	Koshihikari	1937	T	A	646	VAL	GLU	Not different from wild type			
0489W	Koshihikari	2021	T	A	674	LEU	GLN	Not different from wild type			
0424F	Koshihikari	2024	G	A	675	GLY	GLU	Not different from wild type			
0888N	Koshihikari	2146	G	A	716	ALA	THR	Not different from wild type			
0113U	Koshihikari	2168	C	A	723	ALA	ASP	Not different from wild type			
7991J	Toyomeki	274	G	A	92	GLY	ARG	Not different from wild type			
7103J	Toyomeki	310	G	A	104	VAL	MET	Not different from wild type			
7317J	Toyomeki	1001	C	T	334	THR	ILE	Not different from wild type			
7784J	Toyomeki	1504	G	A	502	ASP	ASN	Not different from wild type			
5792J	Toyomeki	1642	C	T	548	PRO	SER	Not different from wild type			
8060J	Toyomeki	1896	G	A	632	MET	ILE	Not different from wild type			

emf3-1D is the allele of *qEMF3*.
Peak time' is classified by the comparison among wild type, Mutant homo, and Hetero in each allele.
Definition of 'Synchrony' is indicated below;
Low: < 0.25
Moderate: 0.25 – 0.50
High: > 0.50

Table S5 . Primer list for selecting single nucleotide substitution lines in TILLING mutant panels.

Name of primer		Sequence (5' → 3')
**Tilling method**	**determination of the mutation site**	
5400_P1_F1	amplification	CGTTTCGTTGCAGATTCCCA
5400_P1_R2	amplification	GCAAGAACATCGCCACCAG
5400_P2_F1	amplification	CATCATGACGGCTTGCGG
5400_P2_R1	amplification	TCACGTTTTCCACTACCTCT
5400_P1_MR1	amplification	ACGAGGTCCATGTGCAGG
5400_P1_MF1	amplification	TGCTGTTCCTCGTCGAGAAG
5400_P2_MR1	amplification	TTCTCGGCCATGATTGTCCT
**SNP**	**Target allele**	
T90S-NIP6-F	*emf-4D*	CGAGCTCGGCGGCTTCGCCATGA
T90S-NIL6-F	*emf-4D*	CGAGCTCGGCGGCTTCGCCATGT
T90S-R	*emf-4D*	GACAGCAGCTGCATCCAGTA
T100I-NIP6-F	*emf-6D*	GCGACCGACTTCTGGTGCATCTC
T100I-NIL3-F	*emf-6D*	GCGACCGACTTCTGGTGCATGAT
T100I-R	*emf-6D*	AGGCCGTAGAAGATGTCGAG
L215R-NIP6-F	*emf-5D*	GCAGTCGCCAAGGAGTGCCACGT
L215R-NIL6-F	*emf-5D*	GCAGTCGCCAAGGAGTGCCACGG
L215R-R	*emf-5D*	AGCCGCTGCTCGTCGTAG
E296G-NIP-F	*emf-8D*	ACGTCGGCGCCGGTCACTGA
E296G-NIL-F	*emf-8D*	ACGTCGGCGCCGGTCACTGG
E296G-R	*emf-8D*	GCTTGTTGAGGATGGAGAGG
D547V-NIP7-F	*emf-7D*	GCCCTCGTCGAGGCGATGAGTCA
D547V-NIL2-F	*emf-7D*	GCCCTCGTCGAGGCGATGAGCCT
D547V-R	*emf-7D*	CCCAGGAAGATGTTGAGCAC
N560I-NIP6-F	*emf-3D*	CCGGCCGCGAGGATCCTCCGCTA
N560I-NIL2-F	*emf-3D*	CCGGCCGCGAGGATCCTCCGCTT
N560I-R	*emf-3D*	GACCTGCACCATCGTCTTC
T563I-NIP-F	*emf-2D*	ATCCTCCGCAACCTGTGTAC
T563I-NIL-F	*emf-2D*	ATCCTCCGCAACCTGTGTAT
T563I-R	*emf-2D*	AGGACCTGCACCATCGTCT
L719*-NIP-F	*emf-9D*	ACGTCGAGGCGGCTCTGGACTT
L719*-NIL-F	*emf-9D*	ACGTCGAGGCGGCTCTGGACTA
L719*-R	*emf-9D*	CCATGGACGATCCTGTTCTT

Table S6. List of *EMF3* and *EMF3*-like gene family members in different plants.

Organism	GeneID/Gene name	Transcript ID	A.A Num	TM No. from SOSUI prediction	TM region from SOSUI prediction					
Oryza sativa	EMF3	*Os03t0145400-00*	723	4	61-83	94-116	132-154	174-196		
Oryza sativa	Os12g0528100	*Os12t0528100-00*	823	2	109-131	134-156				
Oryza sativa	Os03g0363100	*Os03t0363100-00*	851	2	232-253	267-289				
Oryza sativa	Os07g0648000	*Os07t0648000-01*	813	4	104-126	183-205	211-233	246-268		
Oryza sativa	Os03g0362200	*Os03t0362200-01*	853	3	114-136	240-262	278-300			
Sorghum bicolor	Sobic.001G505700	Sobic.001G505700.1.p	731	4	60-82	93-115	137-159	174-196		
Sorghum bicolor	Sobic.001G310600	Sobic.001G310600.1.p	744	4	64-86	98-120	135-157	184-206		
Sorghum bicolor	Sobic.008G113400	Sobic.008G113400.1.p	851	2	85-107	111-133				
Sorghum bicolor	Sobic.001G363300	Sobic.001G363300.1.p	877	3	119-141	253-275	294-316			
Sorghum bicolor	Sobic.001G363200	Sobic.001G363200.1.p	880	3	102-124	266-288	300-322			
Zea mays	Zm00001d027647	A0A1D6JNI3_MAIZE	735	3	80-102	127-149	170-192			
Zea mays	Zm00001d041709	A0A3L6FM85_MAIZE	841	3	92-114	117-139	226-248			
Zea mays	Zm00001d029246	A0A1D6K3V3_MAIZE	869	3	119-141	247-269	284-306			
Zea mays	Zm00001d029247	A0A1D6K3V4_MAIZE	888	6	102-124	271-293	306-328	633-654	723-741	747-768
Hordeum vulgare	4HG0407420	HORVU.MOREX.r3.4HG0407420.1	730	4	63-85	96-118	140-162	175-197		
Hordeum vulgare	4HG0407450	HORVU.MOREX.r3.4HG0407450.1	720	4	65-87	92-114	132-154	170-192		
Hordeum vulgare	5HG0447120	HORVU.MOREX.r3.5HG0447120.1	792	2	96-118	122-144				
Hordeum vulgare	6HG0619500	HORVU.MOREX.r3.6HG0619500.1	839	2	219-240	257-279				
Marchantia polymorpha	Mp6g11110.1	Mapoly0016s0150.1	971	1	114-136					
Marchantia polymorpha	Mp7g16730.1	Mapoly0051s0011.1	999	2	334-355	376-398				
Marchantia polymorpha	Mp5g04190.1	Mapoly0141s0026.1	974	3	176-198	316-338	359-381			
Marchantia polymorpha	Mp2g11750.1	Mapoly0023s0141.1	942	0						
Arabidopsis thaliana	AT5G18980	AT5G18980.1	835	2	88-110	113-135				
Arabidopsis thaliana	AT3G06210	AT3G06210.1	840	2	79-101	112-133				
Arabidopsis thaliana	AT4G14280	AT4G14280.1	798	5	53-75	83-105	193-215	237-259	300-322	
Lotus japonicus	Lj6g3v1887750	Lj6g3v1887750.1	833	4	76-98	109-130	225-247	264-286		
Ipomoea nil	INIL13g40935	INIL13g40935.t1	814	1	79-101					
Ipomoea nil	INIL13g08018	INIL13g08018.t1	844	2	75-97	108-127				
Solanum lycopersicum	Solyc01g080260.3.1	A0A6N2BR67_SOLCI	830	4	70-92	102-124	224-246	262-284		
Vitis vinifera	VIT_05s0020g02500	VIT_05s0020g02500.t01	756	4	72-94	101-123	150-172	191-213		

Outgroup genes

Organism	GeneID/Gene name	transcripts ID	A.A Num
Oryza sativa	Os07g0650200	*Os07t0650200-00*	727
Sorghum bicolor	Sobic.006G009200	Sobic.006G009200.1.p	765
Hordeum vulgare	2HG0118240	HORVU.MOREX.r3.2HG0118240.1	874

A prediction program for membrane proteins, SOSUI was used to predict the transmembrane (TM) domain of membrane proteins.

Table S7. Enriched gene ontology and KEGG pathway in each clusters.
Statistical tests were performed using two-sided Fisher's exact test with Benjamini and Hochberg's correction.

"A & B" indicates genes contained in the window and having an annotation.
"A" indicates genes contained in the window.
"B" indicates genes having an annotation.
"U" indicates genes contained in the parental population.

Cluster ID	GO ID or KEGG PATHWAY	GO or KEGG term	Adjusted *p*-value	A & B	A	B	U
1	GO:0005634	nucleus	6.72897E-05	51	487	505	11062
1	GO:0003677	DNA binding	0.007278212	65	487	867	11062
1	GO:0006259	DNA metabolic process	0.00607044	19	487	137	11062
1	GO:0006325	chromatin organization	0.004818336	14	487	74	11062
1	GO:0008276	protein methyltransferase activity	0.039043776	6	487	19	11062
1	dosa03082	ATP-dependent chromatin remodeling	0.000343814	11	145	52	3844
1	dosa03440	Homologous recombination	0.004519229	8	145	38	3844
2	GO:0016020	membrane	0.029849948	178	1121	1378	11062
2	GO:0016758	transferase activity, transferring hexosyl groups	0.010206777	45	1121	244	11062
2	GO:0005576	extracellular region	2.7096E-07	32	1121	94	11062
2	GO:0004857	enzyme inhibitor activity	2.7096E-07	26	1121	66	11062
2	GO:0006812	cation transport	0.012834742	38	1121	197	11062
2	GO:0055085	transmembrane transport	0.029267683	67	1121	430	11062
2	GO:0008081	phosphoric diester hydrolase activity	0.01043852	13	1121	38	11062
2	GO:0042545	cell wall modification	0.000129705	12	1121	22	11062
2	GO:0005618	cell wall	0.001387079	14	1121	35	11062
2	GO:0030599	pectinesterase activity	2.96926E-14	28	1121	44	11062
2	GO:0015299	solute:proton antiporter activity	0.001433948	10	1121	19	11062
2	GO:0030570	pectate lyase activity	0.015190863	4	1121	4	11062
2	dosa00040	Pentose and glucuronate interconversions	1.94148E-09	20	302	44	3844
2	dosa00564	Glycerophospholipid metabolism	0.00380587	17	302	78	3844
2	dosa01100	Metabolic pathways	0.000448736	176	302	1771	3844
3	GO:0005515	protein binding	0.002806459	187	651	2379	11062
3	GO:0008270	zinc ion binding	0.002806459	76	651	774	11062
3	GO:0008287	protein serine/threonine phosphatase complex	0.00167249	13	651	46	11062
3	GO:0006470	protein dephosphorylation	0.001739928	15	651	62	11062
3	GO:0004722	protein serine/threonine phosphatase activity	0.00167249	12	651	37	11062
3	dosa04075	Plant hormone signal transduction	0.014502652	21	218	164	3844
3	dosa04120	Ubiquitin mediated proteolysis	0.00079885	18	218	97	3844
3	dosa04144	Endocytosis	0.002827206	20	218	132	3844
5	GO:0055085	transmembrane transport	0.031641824	40	512	430	11062
5	GO:0009055	electron transfer activity	0.042090819	26	512	237	11062
5	dosa00190	Oxidative phosphorylation	0.028426818	13	184	102	3844
5	dosa00620	Pyruvate metabolism	0.024522774	12	184	86	3844
5	dosa01100	Metabolic pathways	4.86873E-05	118	184	1771	3844
5	dosa01110	Biosynthesis of secondary metabolites	0.000143219	74	184	967	3844
5	dosa01200	Carbon metabolism	0.000143219	28	184	231	3844
6	GO:0016829	lyase activity	0.037696925	19	541	154	11062
6	GO:0008652	cellular amino acid biosynthetic process	4.61679E-05	20	541	98	11062
6	GO:1901607	alpha-amino acid biosynthetic process	0.004177628	15	541	83	11062
6	dosa00730	Thiamine metabolism	0.009187333	7	228	19	3844
6	dosa01240	Biosynthesis of cofactors	0.029772035	25	228	207	3844
7	GO:0005515	protein binding	0.001208516	167	573	2379	11062
7	GO:0005524	ATP binding	0.027515916	97	573	1352	11062
7	GO:0006468	protein phosphorylation	7.25427E-06	72	573	700	11062
7	GO:0004674	protein serine/threonine kinase activity	0.000920863	46	573	441	11062
7	GO:0006886	intracellular protein transport	0.011449339	21	573	163	11062
7	GO:0016192	vesicle-mediated transport	7.25427E-06	27	573	151	11062
7	GO:0009725	response to hormone	0.017102515	8	573	33	11062
7	GO:0030126	COPI vesicle coat	0.037888692	4	573	9	11062
7	GO:0051274	beta-glucan biosynthetic process	0.001326791	9	573	28	11062
7	GO:0035251	UDP-glucosyltransferase activity	0.002355386	10	573	38	11062
8	GO:0016021	integral component of membrane	0.042369282	46	507	543	11062
9	GO:0030170	pyridoxal phosphate binding	0.034252636	17	863	88	11062
9	GO:0016829	lyase activity	0.017741602	26	863	154	11062
9	GO:0005576	extracellular region	0.013465822	19	863	94	11062
9	GO:0005506	iron ion binding	1.46508E-13	55	863	208	11062
9	GO:0009055	electron transfer activity	1.14511E-11	56	863	237	11062
9	GO:0016667	oxidoreductase activity, acting on a sulfur group of donors	0.034252636	16	863	81	11062
9	GO:0016705	oxidoreductase activity, acting on paired donors, with incorporation or reduction of molecular oxygen	4.77131E-14	57	863	214	11062
9	GO:0051213	dioxygenase activity	0.0096442	17	863	74	11062
9	GO:0020037	heme binding	1.46508E-13	57	863	221	11062
9	GO:0048544	recognition of pollen	0.034252636	8	863	25	11062
9	GO:0030145	manganese ion binding	0.00021539	10	863	20	11062
9	GO:0045735	nutrient reservoir activity	2.29768E-05	9	863	13	11062
9	GO:0006032	chitin catabolic process	0.01053087	6	863	11	11062
9	GO:0016998	cell wall macromolecule catabolic process	0.034252636	6	863	14	11062
9	GO:0004568	chitinase activity	0.01053087	6	863	11	11062
9	GO:0008061	chitin binding	0.01053087	5	863	7	11062
9	GO:0042742	defense response to bacterium	0.034252636	3	863	3	11062
9	GO:0050832	defense response to fungus	0.034252636	3	863	3	11062
9	dosa00010	Glycolysis / Gluconeogenesis	0.020432844	18	277	121	3844
9	dosa00051	Fructose and mannose metabolism	0.020914864	10	277	50	3844
9	dosa00220	Arginine biosynthesis	0.002220139	9	277	30	3844
9	dosa00250	Alanine, aspartate and glutamate metabolism	0.006727528	10	277	42	3844
9	dosa00270	Cysteine and methionine metabolism	0.000127385	20	277	93	3844
9	dosa00350	Tyrosine metabolism	0.020432844	8	277	34	3844
9	dosa00360	Phenylalanine metabolism	0.040428431	7	277	31	3844
9	dosa00380	Tryptophan metabolism	0.040428431	8	277	39	3844
9	dosa00480	Glutathione metabolism	0.00010349	18	277	76	3844
9	dosa00592	alpha-Linolenic acid metabolism	0.006727528	9	277	35	3844
9	dosa00630	Glyoxylate and dicarboxylate metabolism	0.001302364	14	277	62	3844
9	dosa00710	Carbon fixation in photosynthetic organisms	1.02425E-05	18	277	65	3844
9	dosa00900	Terpenoid backbone biosynthesis	0.037591865	9	277	46	3844
9	dosa00904	Diterpenoid biosynthesis	3.47744E-09	16	277	32	3844
9	dosa00940	Phenylpropanoid biosynthesis	0.000510864	18	277	87	3844
9	dosa00950	Isoquinoline alkaloid biosynthesis	0.020432844	5	277	14	3844
9	dosa00999	Biosynthesis of various plant secondary metabolites	0.000247974	12	277	40	3844
9	dosa01100	Metabolic pathways	4.26453E-17	198	277	1771	3844
9	dosa01110	Biosynthesis of secondary metabolites	4.61533E-18	138	277	967	3844

9	dosa01200	Carbon metabolism	8.06462E-06	39	277	231	3844
10	GO:0005524	ATP binding	0.002338491	153	931	1352	11062
10	GO:0005634	nucleus	3.50365E-15	103	931	505	11062
10	GO:0007018	microtubule-based movement	0.016781098	11	931	41	11062
10	GO:0003777	microtubule motor activity	0.003913363	11	931	35	11062
10	GO:0006355	regulation of transcription, DNA-templated	0.002306983	81	931	624	11062
10	GO:0007275	multicellular organism development	0.028670645	7	931	20	11062
10	GO:0140110	transcription regulator activity	0.034506528	40	931	288	11062
10	GO:0000786	nucleosome	1.57858E-06	19	931	51	11062
10	GO:0006334	nucleosome assembly	1.04602E-05	18	931	52	11062
10	GO:0006298	mismatch repair	0.02548783	5	931	10	11062
10	GO:0030983	mismatched DNA binding	0.02548783	5	931	10	11062
10	GO:0006284	base-excision repair	0.02969803	6	931	15	11062
10	GO:0003918	DNA topoisomerase type II (double strand cut, ATP-hydrolyzing) activity	0.009561976	4	931	5	11062
10	GO:0004527	exonuclease activity	0.039244074	8	931	27	11062
10	GO:0006270	DNA replication initiation	0.000180351	6	931	7	11062
10	GO:0015631	tubulin binding	0.02150756	6	931	14	11062
10	GO:0006310	DNA recombination	0.000125849	10	931	20	11062
10	GO:0051103	DNA ligation involved in DNA repair	0.02150756	3	931	3	11062
10	GO:0003910	DNA ligase (ATP) activity	0.02150756	3	931	3	11062
10	GO:0003887	DNA-directed DNA polymerase activity	0.008630784	6	931	12	11062
10	GO:0008812	choline dehydrogenase activity	0.02150756	3	931	3	11062
10	dosa00073	Cutin, suberine and wax biosynthesis	2.0827E-05	10	285	21	3844
10	dosa03030	DNA replication	4.51121E-13	20	285	33	3844
10	dosa03040	Spliceosome	0.015565699	24	285	163	3844
10	dosa03082	ATP-dependent chromatin remodeling	0.001688632	13	285	52	3844
10	dosa03410	Base excision repair	4.43147E-08	16	285	35	3844
10	dosa03420	Nucleotide excision repair	0.018378496	13	285	67	3844
10	dosa03430	Mismatch repair	1.70593E-06	12	285	25	3844
10	dosa03440	Homologous recombination	1.31813E-07	16	285	38	3844
11	GO:0015935	small ribosomal subunit	1.3139E-08	15	1357	20	11062
11	GO:0003735	structural constituent of ribosome	5.34046E-81	172	1357	290	11062
11	GO:0008652	cellular amino acid biosynthetic process	0.007849903	25	1357	98	11062
11	GO:0004386	helicase activity	0.000122785	23	1357	67	11062
11	GO:0006418	tRNA aminoacylation for protein translation	0.000226842	18	1357	47	11062
11	GO:0004812	aminoacyl-tRNA ligase activity	1.08548E-05	21	1357	51	11062
11	GO:0015934	large ribosomal subunit	0.001609785	10	1357	20	11062
11	GO:0006414	translational elongation	0.000127664	15	1357	33	11062
11	GO:0003743	translation initiation factor activity	0.012215481	11	1357	29	11062
11	GO:0006189	'de novo' IMP biosynthetic process	0.031656356	4	1357	5	11062
11	GO:0019843	rRNA binding	0.000141922	11	1357	19	11062
11	GO:0006626	protein targeting to mitochondrion	0.032434342	7	1357	15	11062
11	GO:0019867	outer membrane	0.031656356	9	1357	23	11062
11	GO:0005643	nuclear pore	0.006845852	6	1357	9	11062
11	GO:0003899	DNA-directed 5'-3' RNA polymerase activity	0.000457127	17	1357	45	11062
11	GO:0008033	tRNA processing	0.039532926	11	1357	33	11062
11	GO:0008175	tRNA methyltransferase activity	0.002802251	6	1357	8	11062
11	GO:0006364	rRNA processing	2.72628E-07	16	1357	26	11062
11	GO:0003697	single-stranded DNA binding	0.005102818	8	1357	15	11062
11	GO:0032040	small-subunit processome	0.00743517	4	1357	4	11062
11	GO:0042026	protein refolding	0.033316122	5	1357	8	11062
11	GO:0001510	RNA methylation	0.000486843	7	1357	9	11062
11	GO:0001522	pseudouridine synthesis	0.000522225	10	1357	18	11062
11	GO:0009982	pseudouridine synthase activity	0.000297011	10	1357	17	11062
11	GO:0005730	nucleolus	0.000150914	7	1357	8	11062
11	GO:0005852	eukaryotic translation initiation factor 3 complex	0.00743517	4	1357	4	11062
11	dosa00261	Monobactam biosynthesis	0.023488561	6	573	10	3844
11	dosa00300	Lysine biosynthesis	0.000499413	9	573	13	3844
11	dosa00970	Aminoacyl-tRNA biosynthesis	0.001916854	18	573	47	3844
11	dosa03008	Ribosome biogenesis in eukaryotes	6.08808E-15	41	573	72	3844
11	dosa03010	Ribosome	2.92218E-74	166	573	270	3844
11	dosa03013	Nucleocytoplasmic transport	0.004953556	27	573	91	3844
11	dosa03020	RNA polymerase	0.005200837	14	573	35	3844
11	dosa03040	Spliceosome	6.26165E-06	50	573	163	3844
12	GO:0003676	nucleic acid binding	0.031044266	78	339	1647	11062
12	GO:0005515	protein binding	0.000183551	114	339	2379	11062
12	GO:0008270	zinc ion binding	0.011283758	46	339	774	11062
12	GO:0006357	regulation of transcription by RNA polymerase II	0.044487094	6	339	24	11062
13	GO:0005515	protein binding	8.97132E-05	139	424	2379	11062
13	GO:0005524	ATP binding	0.018914382	79	424	1352	11062
13	GO:0009725	response to hormone	0.001841411	9	424	33	11062
14	GO:0005840	ribosome	5.19985E-06	54	959	291	11062
14	GO:0003723	RNA binding	0.02536302	44	959	306	11062
14	GO:0003735	structural constituent of ribosome	4.7465E-06	54	959	290	11062
14	GO:0030170	pyridoxal phosphate binding	0.021661794	18	959	88	11062
14	GO:0008652	cellular amino acid biosynthetic process	0.028401425	19	959	98	11062
14	GO:1901607	alpha-amino acid biosynthetic process	0.003734176	19	959	83	11062
14	GO:0006457	protein folding	0.000316863	32	959	159	11062
14	GO:0042398	cellular modified amino acid biosynthetic process	0.036577425	8	959	25	11062
14	GO:0006418	tRNA aminoacylation for protein translation	2.84121E-06	18	959	47	11062
14	GO:0004812	aminoacyl-tRNA ligase activity	1.04034E-05	18	959	51	11062
14	GO:0033014	tetrapyrrole biosynthetic process	0.028401425	8	959	24	11062
14	GO:0046148	pigment biosynthetic process	0.028401425	8	959	24	11062
14	GO:0016765	transferase activity, transferring alkyl or aryl (other than methyl) groups	0.02523175	11	959	41	11062
14	GO:0016778	diphosphotransferase activity	0.021661794	5	959	9	11062
14	GO:0009654	photosystem II oxygen evolving complex	4.7465E-06	12	959	22	11062
14	GO:0019898	extrinsic component of membrane	0.000128913	11	959	24	11062
14	GO:0034470	ncRNA processing	0.033889057	13	959	56	11062
14	GO:0009765	photosynthesis, light harvesting	2.27774E-05	7	959	8	11062
14	GO:0042559	pteridine-containing compound biosynthetic process	0.020599496	6	959	13	11062
14	GO:0004616	phosphogluconate dehydrogenase (decarboxylating) activity	0.031165766	4	959	6	11062
14	GO:0009072	aromatic amino acid family metabolic process	0.000167165	14	959	39	11062
14	GO:0006760	folic acid-containing compound metabolic process	0.006788549	7	959	15	11062
14	GO:0009507	chloroplast	0.001753702	9	959	21	11062
14	GO:0072593	reactive oxygen species metabolic process	0.020599496	6	959	13	11062
14	GO:0004312	fatty acid synthase activity	0.028401425	3	959	3	11062
14	GO:0009538	photosystem I reaction center	0.000316611	5	959	5	11062
14	dosa00195	Photosynthesis	6.24346E-23	36	435	48	3844
14	dosa00196	Photosynthesis - antenna proteins	6.04959E-08	12	435	15	3844
14	dosa00400	Phenylalanine, tyrosine and tryptophan biosynthesis	0.000920271	15	435	44	3844

14	dosa00630	Glyoxylate and dicarboxylate metabolism	0.033371558	15	435	62	3844
14	dosa00710	Carbon fixation in photosynthetic organisms	0.008263941	17	435	65	3844
14	dosa00860	Porphyrin metabolism	0.001925612	14	435	42	3844
14	dosa00970	Aminoacyl-tRNA biosynthesis	8.67904E-06	19	435	47	3844
14	dosa01100	Metabolic pathways	2.84846E-12	274	435	1771	3844
14	dosa01110	Biosynthesis of secondary metabolites	0.000705519	145	435	967	3844
14	dosa01200	Carbon metabolism	0.007170685	43	435	231	3844
14	dosa01230	Biosynthesis of amino acids	0.000705519	42	435	200	3844
14	dosa01240	Biosynthesis of cofactors	0.016680583	38	435	207	3844
14	dosa03010	Ribosome	0.000147014	55	435	270	3844
15	GO:0005777	peroxisome	0.004847549	6	490	17	11062
15	GO:0006099	tricarboxylic acid cycle	0.004847549	7	490	24	11062
15	GO:0006096	glycolytic process	0.041814639	8	490	48	11062
15	GO:0016881	acid-amino acid ligase activity	0.01348499	9	490	48	11062
15	GO:0046961	proton-transporting ATPase activity, rotational mechanism	2.2024E-06	9	490	16	11062
15	GO:0033179	proton-transporting V-type ATPase, V0 domain	1.78168E-07	7	490	7	11062
15	GO:0015986	ATP synthesis coupled proton transport	0.016486461	5	490	15	11062
15	GO:0004129	cytochrome-c oxidase activity	0.01348499	4	490	8	11062
15	GO:0046933	proton-transporting ATP synthase activity, rotational mechanism	0.001655149	6	490	14	11062
15	GO:0033180	proton-transporting V-type ATPase, V1 domain	4.22272E-05	6	490	8	11062
15	GO:0016417	S-acyltransferase activity	0.036348351	3	490	5	11062
15	GO:0005746	mitochondrial respirasome	0.016361406	3	490	4	11062
15	GO:0006635	fatty acid beta-oxidation	0.016361406	3	490	4	11062
15	GO:0008121	ubiquinol-cytochrome-c reductase activity	0.000358491	6	490	11	11062
15	GO:0004347	glucose-6-phosphate isomerase activity	0.016361406	3	490	4	11062
15	dosa00010	Glycolysis / Gluconeogenesis	0.042244097	15	206	121	3844
15	dosa00020	Citrate cycle (TCA cycle)	0.011517648	10	206	53	3844
15	dosa00190	Oxidative phosphorylation	5.11583E-26	42	206	102	3844
15	dosa01100	Metabolic pathways	0.000510501	125	206	1771	3844
15	dosa01200	Carbon metabolism	0.006845816	26	206	231	3844
15	dosa04145	Phagosome	7.86646E-10	21	206	67	3844
16	GO:0016021	integral component of membrane	0.00104556	69	832	543	11062
16	GO:0030163	protein catabolic process	0.003061492	22	832	113	11062
16	GO:1905368	peptidase complex	0.023406089	10	832	38	11062
16	GO:0006886	intracellular protein transport	1.26031E-13	47	832	163	11062
16	GO:0030120	vesicle coat	0.008844857	8	832	22	11062
16	GO:0006888	endoplasmic reticulum to Golgi vesicle-mediated transport	0.013463242	7	832	18	11062
16	GO:0005543	phospholipid binding	0.008139827	9	832	27	11062
16	GO:0030131	clathrin adaptor complex	0.000863344	7	832	12	11062
16	GO:0000139	Golgi membrane	0.013085292	6	832	13	11062
16	GO:0005351	carbohydrate:proton symporter activity	0.02505488	4	832	6	11062
16	dosa03050	Proteasome	3.10307E-06	18	274	58	3844
16	dosa04070	Phosphatidylinositol signaling system	0.025696496	11	274	51	3844
16	dosa04075	Plant hormone signal transduction	0.032251051	23	274	164	3844
16	dosa04130	SNARE interactions in vesicular transport	0.005081224	10	274	35	3844
16	dosa04144	Endocytosis	1.66666E-07	31	274	132	3844

Table S8. Clustering of jasmonate-related genes.
The cluster ID of each gene is shown. The cluster ID of the genes not used for the clustering is shown as NA.

Locus ID	Gene name	Cluster id
Os11g0146600	*OsDAD1;1*	NA
Os08g0143600	*OsDAD1;2*	NA
Os02g0653900	*OsDAD1;3*	7
Os10g0562200	*OsDAD1;4*	NA
Os07g0520900	*OsPLA1*	16
Os08g0508800	*OsLOX2;1*	9
Os12g0559200	*OsLOX2;2*	5
Os08g0509100	*OsLOX2;3*	9
Os02g0194700	*OsLOX2;4*	NA
Os03g0179900	*OsLOX2;5*	9
Os04g0447100	*LOX*	15
Os05g0304600	*LOX*	5
Os03g0767000	*AOS1*	16
Os03g0225900	*AOS2*	NA
Os02g0218700	*AOS3*	NA
Os02g0218800	*AOS4*	NA
Os03g0438100	*AOC*	16
Os06g0216300	*OPR1*	15
Os06g0216200	*OPR2*	NA
Os06g0216000	*OPR3*	NA
Os06g0215900	*OPR4*	9
Os06g0215600	*OPR5*	9
Os06g0215500	*OPR6*	NA
Os08g0459600	*OPR7*	3
Os02g0559400	*OPR8*	14
Os01g0370000	*OPR9*	NA
Os01g0369900	*OPR10*	2
Os03g0132000	*OsOPCL1*	15
Os06g0103500	*OsACX(AOX)*	15
Os02g0274100	*OaAIM1(MFP)*	15
Os01g0159400	*AOX*	14
Os06g0354500	*AOX*	15
Os11g0605500	*AOX*	9
Os05g0163700	*AOX*	5
Os06g0346300	*AOX*	NA
Os06g0347100	*AOX*	13
Os05g0155000	*MFP*	NA
Os01g0348600	*MFP*	16
Os05g0362100	*MFP*	10
Os10g0457600	*PKT3*	8
Os02g0817700	*OsKAT(PKT3)*	16
Os01g0701700	*OsJMT1*	NA
Os06g0329900	*OsJMT2*	2
Os06g0314600	*OsJMT3*	NA
Os06g0323100	*OsJMT4*	NA
Os05g0102000	*OsJMT5*	NA
Os06g0315000	*OsJMT6*	NA
Os05g0586200	*JAR1*	2
Os01g0221100	*JAR2*	NA

Os11g0186500	*JAR3*	NA	
Os11g0483000	*HAN1(CYP94B4)*		3
Os12g0443000	*CYP94B5*		16
Os11g0151400	*CYP94C2a*		2
Os01g0858350	*CYP94C3*		8
Os05g0445100	*CYP94C4*		4
Os12g0150200	*CYP94C2b*		2
Os04g0521800	*OsAH1*		4
Os07g0249700	*OsAH2*		5
Os07g0249900	*OsAH3*		3
Os07g0249800	*OsAH4*	NA	
Os03g0836900	*OsAH5*	NA	
Os03g0836800	*OsAH6*	NA	
Os06g0691400	*OsAH7(ILL6)*		2
Os01g0560000	*OsAH8(IAR3)*		3
Os01g0706900	*OsAH9*		2
Os07g0603600	*OsMESL*		2
Os04g0653000	*OsJAZ1*		16
Os07g0153000	*OsJAZ2*		2
Os08g0428400	*OsJAZ3*		16
Os09g0401300	*OsJAZ4*		3
Os04g0395800	*OsJAZ5*		2
Os03g0402800	*OsJAZ6*		3
Os07g0615200	*OsJAZ7*		4
Os09g0439200	*OsJAZ8*		9
Os03g0180800	*OsJAZ9*		3
Os03g0181100	*OsJAZ10*		16
Os03g0180900	*OsJAZ11*		3
Os10g0392400	*OsJAZ12*		3
Os10g0391400	*OsJAZ13*		1
Os10g0391801	*OsJAZ14*	NA	
Os03g0396500	*OsJAZ15*		2
Os10g0575000	*OsMYC2*		16
Os01g0705700	*OsMYC3*		16
Os01g0865600	*OsMYC4*		11
Os01g0853400	*OsCOI1a*		8
Os05g0449500	*OsCOI1b*		7
Os03g0265500	*OsCOI2*		2
Os05g0558800	*OsNINJA1*		16
Os08g0162100	*TPL*		4
Os03g0741100	*OsbHLH148*		9
Os04g0301500	*RERJ1*		13
Os09g0306700	*MED25*		13

Table S9. Enriched gene ontology and KEGG pathway in DEGs between IR64 and *emf3-1D* in each spikelet's organ at 7:30.
Statistical tests were performed using two-sided Fisher’s exact test with Benjamini and Hochberg’s correction.

"A & B" indicates genes contained in the window and having an annotation.
"A" indicates genes contained in the window.
"B" indicates genes having an annotation.
"U" indicates genes contained in the parental population.

Organ	Up / downregulated in *emf3-1D*	GO ID or KEGG PATHWAY	GO or KEGG term	Adjusted p-value	A...B	A	B	U
lemma and palea	up	GO:0016491	oxidoreductase activity	0.039992405	37	201	975	11063
lemma and palea	up	dosa00940	Phenylpropanoid biosynthesis	0.006992325	8	66	87	3845
lemma and palea	up	dosa01110	Biosynthesis of secondary metabolites	0.001462374	33	66	967	3845
lemma and palea	down	dosa04016	MAPK signaling pathway - plant	0.025041764	6	42	96	3845
lemma and palea	down	dosa04075	Plant hormone signal transduction	0.000977413	10	42	164	3845
lemma and palea	down	dosa04141	Protein processing in endoplasmic reticulum	0.015959408	8	42	156	3845
pistil	down	GO:0006355	regulation of transcription, DNA-templated	1.78365E-08	27	109	624	11063
pistil	down	GO:0003700	DNA-binding transcription factor activity	1.33451E-06	16	109	272	11063
pistil	down	GO:0043565	sequence-specific DNA binding	0.000899651	10	109	175	11063
pistil	down	GO:0006869	lipid transport	0.025372455	5	109	62	11063
pistil	down	GO:0006470	protein dephosphorylation	0.025372455	5	109	62	11063
pistil	down	GO:0004722	protein serine/threonine phosphatase activity	0.002730845	5	109	37	11063
pistil	down	dosa04016	MAPK signaling pathway - plant	3.43017E-06	9	30	96	3845
pistil	down	dosa04075	Plant hormone signal transduction	0.000177477	9	30	164	3845
anther	up	GO:0019842	vitamin binding	0.036358151	15	563	116	11063
anther	up	GO:0015980	energy derivation by oxidation of organic compounds	0.036358151	8	563	40	11063
anther	up	GO:0005975	carbohydrate metabolic process	0.044717163	41	563	487	11063
anther	up	GO:0046939	nucleotide phosphorylation	0.041684132	10	563	61	11063
anther	up	GO:0005576	extracellular region	0.000220479	19	563	94	11063
anther	up	GO:0004857	enzyme inhibitor activity	0.024554408	11	563	66	11063
anther	up	GO:0000287	magnesium ion binding	0.027045506	12	563	78	11063
anther	up	GO:0003779	actin binding	0.007820689	9	563	35	11063
anther	up	GO:0030599	pectinesterase activity	4.28056E-05	14	563	45	11063
anther	up	GO:0015299	solute:proton antiporter activity	0.016950484	6	563	19	11063
anther	up	GO:0015986	ATP synthesis coupled proton transport	0.007820689	6	563	15	11063
anther	up	GO:0004129	cytochrome-c oxidase activity	0.023022638	4	563	8	11063
anther	up	GO:0006536	glutamate metabolic process	0.0142427	4	563	7	11063
anther	up	GO:0000275	mitochondrial proton-transporting ATP synthase complex, catalytic sector F(1)	0.010647228	3	563	3	11063
anther	up	GO:0046933	proton-transporting ATP synthase activity, rotational mechanism	0.024554408	5	563	14	11063
anther	up	GO:0005746	mitochondrial respirasome	0.026247438	3	563	4	11063
anther	up	GO:0008121	ubiquinol-cytochrome-c reductase activity	0.010235804	5	563	11	11063
anther	up	GO:0019953	sexual reproduction	0.010235804	5	563	11	11063
anther	up	dosa00040	Pentose and glucuronate interconversions	0.019198732	10	254	45	3845
anther	up	dosa00190	Oxidative phosphorylation	3.12129E-14	34	254	102	3845
anther	up	dosa01100	Metabolic pathways	6.44515E-10	169	254	1772	3845
anther	up	dosa01110	Biosynthesis of secondary metabolites	0.014271398	88	254	967	3845
anther	down	GO:0006355	regulation of transcription, DNA-templated	0.012919024	31	255	624	11063
anther	down	GO:0004722	protein serine/threonine phosphatase activity	0.031606066	6	255	37	11063
anther	down	dosa04075	Plant hormone signal transduction	6.84044E-06	17	84	164	3845
lodicule	up	GO:0006412	translation	6.33447E-21	58	340	401	11063
lodicule	up	GO:0005840	ribosome	2.07991E-23	53	340	291	11063
lodicule	up	GO:0003735	structural constituent of ribosome	2.07991E-23	53	340	290	11063

lodicule	up	GO:0019842	vitamin binding	0.012879883	12	340	116	11063
lodicule	up	GO:0050661	NADP binding	0.047026016	6	340	39	11063
lodicule	up	GO:0006096	glycolytic process	0.006836117	8	340	48	11063
lodicule	up	GO:0006633	fatty acid biosynthetic process	0.009681106	9	340	65	11063
lodicule	up	GO:0016705	oxidoreductase activity, acting on paired donors, with incorporation or reduction of molecular oxygen	0.017106892	17	340	214	11063
lodicule	up	GO:0020037	heme binding	0.009775615	18	340	221	11063
lodicule	up	GO:0006833	water transport	0.007642442	3	340	4	11063
lodicule	up	GO:0006869	lipid transport	0.026210748	8	340	62	11063
lodicule	up	GO:0008289	lipid binding	0.04254512	8	340	67	11063
lodicule	up	GO:1902600	proton transmembrane transport	0.026210748	5	340	23	11063
lodicule	up	GO:0004576	oligosaccharyl transferase activity	0.025542637	3	340	6	11063
lodicule	up	GO:0009678	pyrophosphate hydrolysis-driven proton transmembrane transporter activity	0.014755425	3	340	5	11063
lodicule	up	GO:0003746	translation elongation factor activity	0.021970389	5	340	22	11063
lodicule	up	dosa00010	Glycolysis / Gluconeogenesis	0.044055832	14	186	121	3845
lodicule	up	dosa00051	Fructose and mannose metabolism	0.015278749	9	186	50	3845
lodicule	up	dosa00710	Carbon fixation in photosynthetic organisms	0.000668664	13	186	65	3845
lodicule	up	dosa01200	Carbon metabolism	0.011569905	24	186	231	3845
lodicule	up	dosa01230	Biosynthesis of amino acids	0.015278749	21	186	200	3845
lodicule	up	dosa03010	Ribosome	7.08373E-20	55	186	270	3845
lodicule	down	GO:0006355	regulation of transcription, DNA-templated	0.013702908	36	307	624	11063
lodicule	down	dosa04016	MAPK signaling pathway - plant	0.00669683	10	94	96	3845
lodicule	down	dosa04075	Plant hormone signal transduction	2.21526E-08	21	94	164	3845

Table S10. Flower opening time of NILs carrying *emf3-1D* allele in the heat escape test.

Recurrent parent	*EMF3* allele	Temperature from 12:00 to14:00 (°C)	FOT	Estimated temperature during flower opening (°C)
Caiapo	WT	30.0	12:30-14:00	30.0
Caiapo	*emf3-1D*	30.0	9:00-11:00	27.5-29.2
Caiapo	WT	37.0	11:00-12:30	35.0-37.0
Caiapo	*emf3-1D*	37.0	8:30-10:30	30.0-34.0
Swarna	WT	30.0	11:00-12:30	29.2-30.0
Swarna	*emf3-1D*	30.0	9:00-11:00	27.5-29.2
Swarna	WT	37.0	10:00-11:30	33.0-36.0
Swarna	*emf3-1D*	37.0	9:00-10:30	31.0-34.0

Table S11. Sequences of primers used in this study.

Name of primer	Sequence (5'-3')
5989_F1	AGACCGATTGTTGTTGATCCT
5989_R1	ATACTTCCTGTGTGGCAAGG
5300_F1	CCCTTGCCACACAGGAAGT
5300_R1	AGGCGGCTCTGGAGTTGAT
5400_F1	CTCGACAAGATCATCTCCTACTCG
5400_R1	CCCAGGAAGATGTTGAGCAC
Actin_F	TCTTGGCATCTCTCAGCACA
Actin_R	CTTAGCATTCTTGGGTCCGA
HPT_F	TCGTGCTTTCAGCTTCGATG
HPT_R	TCCATCACAGTTTGCCAGTG
5400_F2	AAGGTGCTCAACATCTTCCTG
5400_R2	AGGCCTGCCTCATCAACTC
DFOT1_F	GTGCCGTACGAGTCCAAGG
DFOT1_R	TGGTAGAACGTGCTCTGGAAC
3'RACE F	GTGGTGAAGAGGCTGGCTA
5'RACE R	CTAGCCAGCCTCTTCACCAC
5400_F0_i	CACACCCGTACATCGTACAA
5400_1111R_i	CTTGCCGATCTTGTCGCAGA
5400_923F_i	TCAGCTGGAAGAACTCGTC
5400_11R_i	TTAGGAGTCCATGTATAATCAACA
5400_P10_F	GCACGTGCCTGATCTATCCT
5400_P10_R	GTTTCAGGCAACTAAGAATGAACTC
18S_F	ATGATAACTCGACGGATCGC
18S_R	CTTGGATGTGGTAGCCGTTT
